# Molecular Calcium Phosphides and Mixed Phosphide Hydrides

**DOI:** 10.1021/acs.inorgchem.5c05335

**Published:** 2025-12-24

**Authors:** Kyle G. Pearce, Luke Teh, Andrew S. S. Wilson, Michael S. Hill

**Affiliations:** Department of Chemistry, 1555 University of Bath, Claverton Down, Bath BA2 7AY, U.K.

## Abstract

[(BDI)­Ca-μ-H]_2_ (BDI = HC­{(Me)­CNDipp}_2_, Dipp = 2,6-*i*-Pr_2_C_6_H_3_), has been employed to synthesize diorganophosphido- and bis­(trimethylsilyl)­phosphidocalcium derivatives. Reaction with two equivalents of Ph_2_PH yielded [(BDI)­Ca-μ-PPh_2_]_2_, which displays two differentiated (μ_2_–Ca–P-Ca versus Ca–P–C_6_H_5_···Ca) phosphide bridging modes in the solid state. Although this compound may be employed to synthesize the calcium phosphaguanidinate, [(BDI)­Ca­{CyNC­(PPh_2_)­NCy}], by reaction with CyN=C=NCy and is a likely intermediate for the catalytic addition of Ph_2_PH to unsaturated substrates, any enhancement in reactivity is only marginal in comparison to previous reports of previous calcium precatalysts. [(BDI)­Ca-μ-H]_2_ reacts incompletely with less acidic or more sterically encumbered phosphines. Selective replacement of a single hydride function and the generation of [(BDI)­Ca­(μ_2_–H)­(μ_2_-PR_2_)]_2_ was, however, achieved by transmetalation with Hg­(PR_2_)_2_ (R = *t*-Bu and SiMe_3_). While the remaining hydride in these compounds was resistant to further reactions, a μ_2_-H_2_–Zn_2_-bridged calcium zincate was identified as a minor product from treatment of [(BDI)­Ca­(μ_2_–H)­(μ_2_-P*t*-Bu_2_)]_2_ with ZnMe_2_. In contrast, heating (40 °C) of [(BDI)­Ca-μ-H]_2_ with two equivalents of P­(SiMe_3_)_3_ induced Me_3_SiH elimination to provide [(BDI)­Ca-μ-P­(SiMe_3_)_2_]_2_. This highly labile compound also presents two differentiated modes of phosphide bridging, via direct engagement of a single phosphide center with both calcium atoms as well as a combination of terminal Ca–P and Ca′···H_3_CSi interactions.

## Graphical abstract

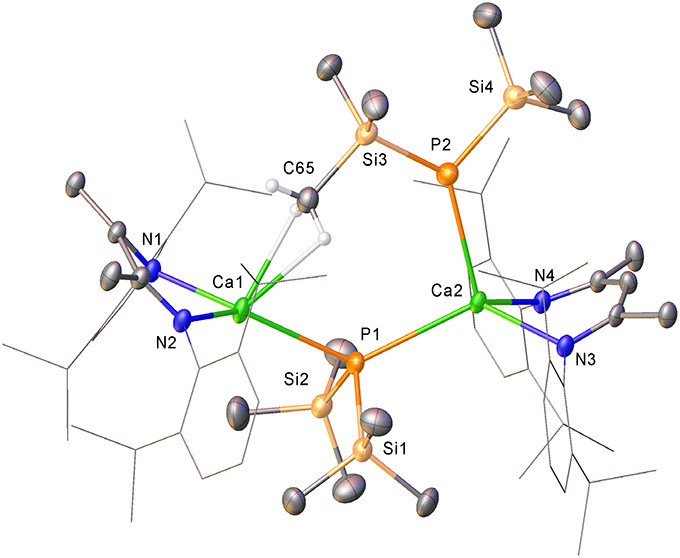

## Introduction

Catalytic hydrophosphination of C-E multiple bonds (E = e.g., CR, CR_2_, O, NR, Figure 1a) provides an atom-efficient method of P–C bond formation.^1–5^ Since an initial focus on the exploitation of less terrestrially available precious metals,^6,7^ a rich transition metal-based chemistry has emerged with more Earth-abundant candidates of lower toxicity proving particularly attractive.^8–20^ Somewhat inspired by redox-inactive 4f-element-based catalysis,^21–27^ systems derived from the similarly electropositive elements of the periodic groups 1 and 2 have also emerged as basic reagents that fulfill these advantageous criteria.^28,29^ Although precedents remain limited, a very recent report by the groups of Krämer and Mulvey has signposted the dramatic enhancements in rate and reaction scope resulting from the catalytic action of the heaviest group 1 (Rb, Cs) phosphides for the hydrophosphination of alkenes and alkynes.^30^ With regard to group 2, a focus has similarly been directed to the heavier elements of the series (Ca, Sr, Ba).^31^ Carpentier and Sarazin, for example, have highlighted the beneficial attributes of β-diketiminato- and iminoanilido-barium species for the hydrophosphination of styrene with Ph_2_PH and Cy_2_PH.^32^ Our own initial contribution to this area demonstrated that the THF-adducted β-diketiminato calcium silazide, [(BDI)­Ca­{N­(SiMe_3_)_2_}­(THF)] (**1**; BDI = HC­{(Me)­CNDipp}_2_ where Dipp = 2,6-*i*-Pr_2_C_6_H_3_), is a capable precatalyst for the intermolecular (*anti*-Markovnikov) addition of Ph_2_PH to styrene and conjugated dienes, while catalytic P–C bond formation could also be achieved with carbodiimide substrates.^33,34^ This early work suggested that the reactivity summarized in Figure 1b invokes a C–P bond forming step based on polarized C=C insertion into the Ca-PPh_2_ bond of the isolable calcium phosphide (**2**). This deduction was later contradicted by a theoretical study that favored the operation of an outer sphere conjugate addition process with no direct interaction between calcium and the vinyl functionality.^35^ Subsequent reports of related calcium- and limited examples of magnesium-derived catalysis,^36–41^ however, for example Westerhausen’s use of [Ca­(PPh_2_)_2_(THF)_4_] to catalyze the reaction of Ph_2_PH with both diphenylethyne and diphenylbutadiyne and Waterman’s very recent study of the effects of blue light irradiation on the catalytic addition of Ph_2_PH to conjugated alkenes with **1** and **2**,^42–45^ indicate that further development of this reactivity is merited.

**Figure 1:**
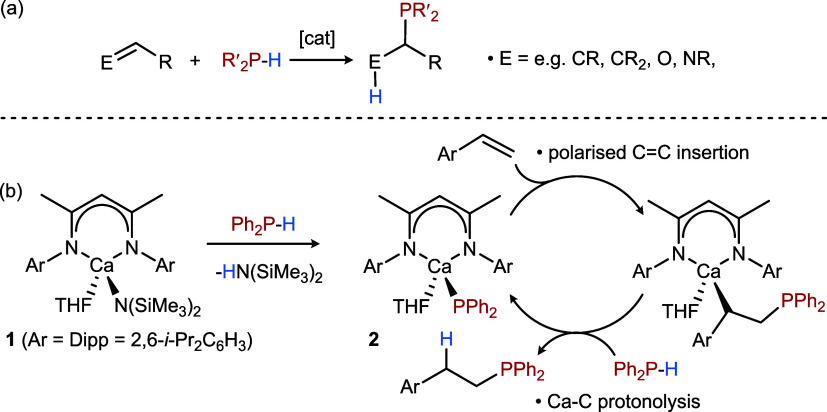
(a) Markovnikov-directed catalytic hydrophosphination of C=E multiple bonds; (b) proposed mechanism of *anti*-Markovnikov hydrophosphination of styrenes catalyzed by **1** and through the intermediacy of **2**.^34^

While a variety of other molecular P–Ca bonded species have been described,^46–52^ the scope of β-diketiminate-based calcium phosphides, notwithstanding two related phosphidoborane derivatives,^53^ remains limited to the THF adduct, compound **2**. In this contribution, therefore, we describe our efforts to address this lacuna and our initial observations of the reactivity of the resultant species.

## Results and Discussion

The molecular calcium hydride, [(BDI)­Ca-μ-H]_2_ (**3**), acts as a highly basic calcium β-diketiminate derivative that is free of coordinated solvent.^54–56^ Our study commenced, therefore, with the synthesis of a THF-free variant of compound **2** through the reaction of **3** with two molar equivalents of Ph_2_PH in benzene (Scheme 1). Addition of the phosphine induced an immediate effervescence and the development of a red coloration to the solution. Analysis by NMR spectroscopy at this point indicated the complete consumption of the calcium hydride starting material and the formation of a single new BDI-containing species (**4**). Compound **4** was most clearly characterized by its ^31^P­{^1^H} spectrum, which comprised a resonance at δ −16.9 ppm that is only marginally downfield of that observed for compound **2** (−21.3 ppm),^34^ and which implies the provision of a similar electronic environment to the phosphorus centers across both compounds. Extended monitoring of the corresponding ^1^H and ^13^C NMR spectra of compound **4** revealed no evidence of Schlenk-like equilibration to [(BDI)_2_Ca] or other phosphidocalcium species, behavior that provides a notable contrast to our earlier observations of the solution stability of compound **2**.

**Scheme 1:**
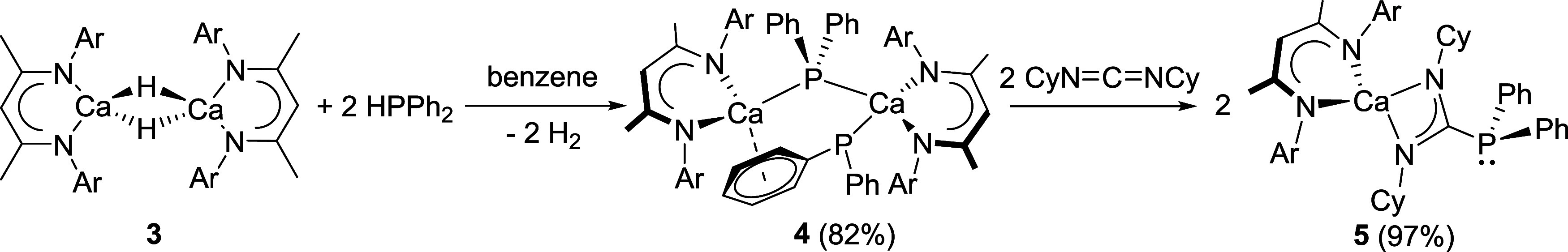
Synthesis of Compounds **4** and **5** (Values in Parentheses Indicate the Isolated Yields)

Plausible insight into the apparently more robust nature of **4** was provided by its crystallization from hexane and a single crystal X-ray diffraction experiment. This analysis (Figure 2a, with selected bond length and angle data presented in Table 1) confirmed that this solvent-free variant adopts a dimeric structure propagated by the diphenylphosphide ligands. Although both phosphide anions connect the [(BDI)­Ca] units, the bridging modes and resultant impact on the coordination geometries of the calcium centers contrast significantly. Whereas the P1-containing anion presents a conventional and quite symmetrically disposed μ_2_–Ca–P-Ca bridging interaction [Ca1–P1 2.9619(6), Ca2–P1 2.9773(7) Å; Ca1–P1–Ca2 124.27(2)°], the remaining (P2) diphenylphosphide center is coordinated solely through a terminal Ca–P interaction to Ca2 with a bond length [Ca2–P2 2.9472(7) Å] that is somewhat elongated in comparison to that previously observed in **2** [2.872(4) Å].^34^ Irrespective of the resultant lower nuclearity of P2, this anion maintains its role as a bridging ligand through η^6^-engagement with Ca1 via the π system of its C77-containing phenyl substituent [Ca1···centroid 2.597 Å]. Presumably due to the steric requirements of this interaction, Ca1 is displaced some 1.61 Å from the least-squares plane defined by its coordinated β-diketiminate ligand, albeit this adjustment impacts only marginally on the relevant Ca–N bonds across both [(BDI)­Ca] moieties [Ca1–N1 2.3344(15), Ca1–N2 2.3150(16), Ca2–N3 2.3427(18), Ca2–N4 2.3947(17) Å]. Although we have previously commented on the role and persistence of such arene interactions in a variety of molecular calcium derivatives,^53,57–61^ the apparent C_2_ symmetry indicated by the simplicity of the room temperature NMR spectra argues that this η^6^–P-C_6_H_5_···Ca interaction does not persist in arene solvents. This evident fluxionality was, therefore, investigated by variable temperature NMR experiments performed in *d*_8_-toluene. Although the ^31^P­{^1^H} NMR spectrum was unchanged down to the low temperature limit (208 K), the corresponding ^1^H NMR spectra indeed resolved as two discriminated BDI ligand environments at temperatures below 228 K. On the assumption that this asymmetry arises from the structural differentiation of the [(BDI)­Ca] units identified in the solid-state analysis of **4**, these data allowed the upper limit on the barrier (Δ*G* ^‡^) toward interconversion of the phosphide bridging modes to be estimated as 40.9 kJ mol^–1^.

**Figure 2:**
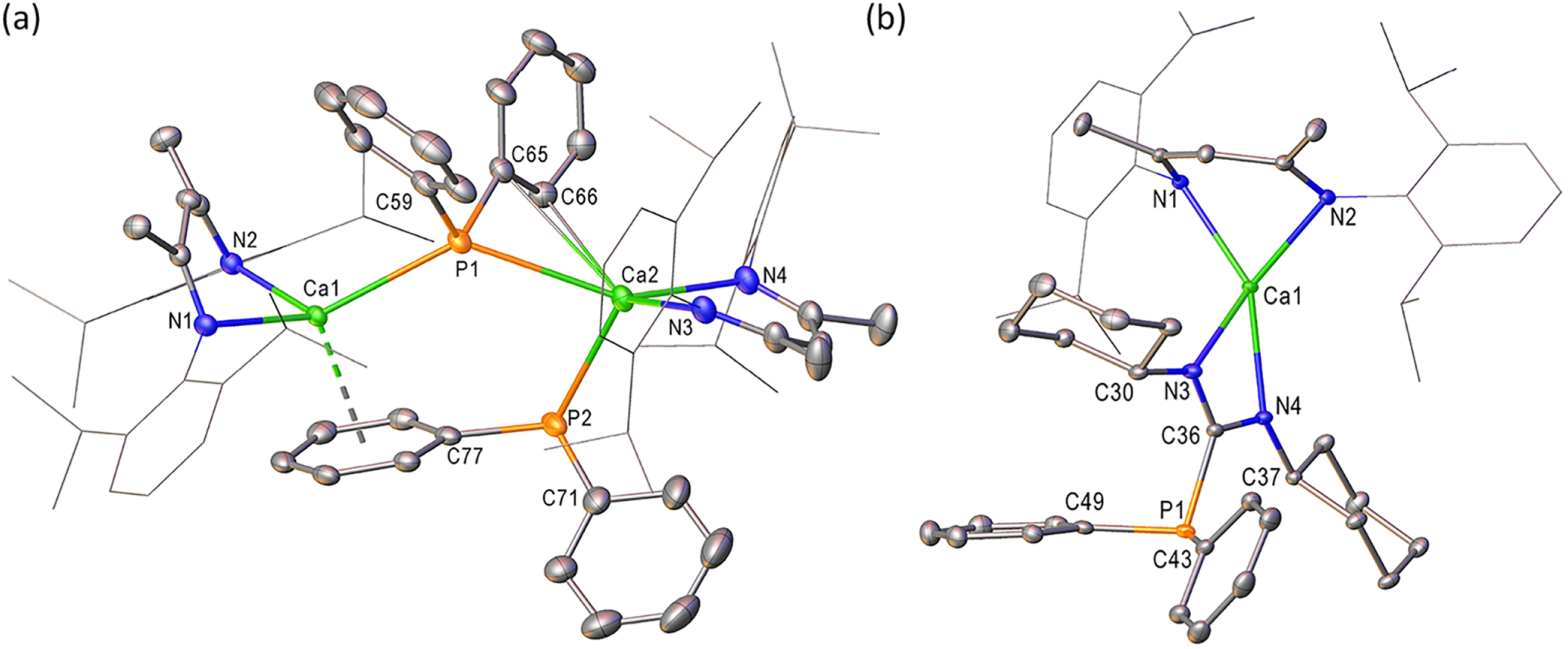
Displacement ellipsoid plots (30% probability) of (a) compound **4**, (b) the Ca1-containing molecule of compound **5**. Hydrogen atoms have been removed for clarity and wireframe view has been employed for the Dipp substituents of both compounds, also for visual ease.

**Table 1:** Selected Bond Lengths (Å) and Angles (°) for Compounds **4**–**7** and **9**

	4	5	6	7	9
Ca1–N1	2.3344(15)	2.3487(10)	2.331(2)	2.374(2)	2.310(4)
Ca1–N2	2.3150(16)	2.3451(10)	2.365(2)	2.335(2)	2.329(4)
Ca1–P1	2.9619(6)	2.3405(11)^a^	2.9791(8)	2.9676(9)	2.9399(15)
Ca2–P1	2.9773(7)	2.3799(10)^b^	2.9559(8)	2.9245(9)	3.0106(16)
Ca2–P2	2.9472(7)	1.9101(12)^c^	-	-	2.8320(17)
Ca2–N3	2.3427(18)	1.3283(16)^d^	2.346(2)	2.351(2)	2.364(3)
Ca2–N4	2.3947(17)	1.3356(16)^e^	2.350(2)	2.352(2)	2.350(4)
N1–Ca1–N2	85.16(5)	82.72(3)	80.62(7)	79.94(7)	81.90(13)
N3–Ca2–N4	81.26(6)	57.25(4)^f^	81.01(7)	81.75(8)	81.09(12)
Ca1–P1–Ca2	124.27(2)	126.82(9)^g^	78.52(2)	80.40(2)	128.96(6)

a Ca1–N3.

b Ca1–N4.

c P1–C36;

d C36–N3.

e C36–N4.

f N3–Ca1–N4.

g N3–C36–P1.

We, and others, have previously reported on the kinetic competence of calcium amides and phosphides such as **1** and **2** to effect the catalytic hydrophosphination of *N*,*N*-diorganocarbodiimides.^33,62,63^ In such cases, the likely catalytic resting states have been identified as calcium phosphaguanidinate species formed prior to protonolysis and resulting from polarized insertion of RN=C=CNR into a phosphidocalcium Ca–P bond. As an initial assessment of its proclivity for similar behavior, therefore, compound **4** was treated with two molar equivalents of dicyclohexylcarbodiimide. Combination of both reagents in benzene solution induced an instantaneous color change from red to colorless, whereupon removal of volatiles and crystallization from hexane provided compound **5** in effective stoichiometric (97%) yield (Scheme 1). The ^31^P­{^1^H} NMR spectroscopic data (δ −19.1 ppm) provided by **5** were consistent with its formulation as a phosphaguanidinate product and bore close comparison to the analogous chemical shifts provided by both the similarly BDI-supported [(BDI)­Ca­{Ph_2_PC­(N*i*-Pr)_2_}­(THF)] (δ −21.1 ppm)^34^ and Westerhausen and co-workers’ bis-phosphaguanidinate product, [*trans*-Ca­{Ph_2_PC­(NCy)_2_}_2_(THF)_2_] (δ −21.5 ppm), derived by reaction of the identical cyclohexyl-susbstituted heterocumulene and calcium bis-diphenylphosphide in THF solution.^63^ This supposition was again confirmed by X-ray difffraction analysis performed on single crystals of **5** grown from hexane, the results of which are shown in Figure 2b with selected bond length and angle data presented in Table 1.

Although the various Ca–N interactions [Ca1–N1 2.3487(10), Ca1–N2 2.3451(10), Ca1–N3 2.3405(11), Ca1–N4 2.3799(10); Ca2–N5 2.3443(10), Ca2–N6 2.3714(10), Ca2–N7 2.3475(10), Ca2–N8 2.4053(10) Å] of both independent molecules within the unit cell of **5** lie within the normal expected range,^43^ the respective Ca1 and Ca2 centers reside some 1.60 and 1.66 Å out of the least-squares planes defined by their coordinated BDI ligands. The mean planes comprising, for example, [Ca1, N1, N2] and [N1, N2, C1, C2, C3, C4, C5] consequently subtend an angle of ca. 64° and leave the calcium centers with an apparently vacant coordination site. In a similar manner to our previous observations of the formamidinate species, [(BDI)­Ca­(*t*-BuNC­(H)*t*-Bu)], which presented a similarly exposed geometry at calcium,^64^ however, no asymmetry of the phosphaguanidinate or BDI environments could be identified in the solution NMR spectra. We are satisfied, therefore, to ascribe this feature of the structure of **5** to a solid state packing effect.

With the viability of both phosphide formation and Ca–P carbodiimide insertion established, a brief assessment of the aptitude of **3** for the catalytic addition of Ph_2_PH to both CyN=C=CNCy and several representative alkenes was undertaken. Although a stoichiometric reaction of **5** and Ph_2_PH provided only limited evidence for the regeneration of **4** (Figures S13 and S14), monitoring of a reaction by ^31^P­{^1^H} NMR spectroscopy with a 5 mol % precatalytic loading of **3** in benzene at 40 °C successfully yielded the phosphaguanidine product over a period of 16 h. The results of this study (Table 2, entry 1) indicated that conversion to CyN­(H)­C­(PPh_2_)=CNCy occurs with a comparable efficacy as that previously achieved for this transformation with compound **1**.^33^ The similarly high conversion of styrene to PhCH_2_CH_2_PPh_2_ achieved under these conditions (entry 2), provides a marginal enhancement in catalytic aptitude in comparison to our earlier study of the use of **1** (95% conversion, 10 mol %, 75 °C for 20 h).^34^ This result, however, continues to evidence a lower activity than that provided by Sarazin’s use of 2 mol % of an iminoanilidobarium species for the same transformation (96% conversion, 15 min), albeit this latter reaction was performed at a mildly elevated temperature (60 °C).^32^ Similarly, although **3** provided high levels of *anti*-Markovnikov P–H addition to 1,1-diphenylethene (entry 3), this activity compares unfavorably with Mulvey and co-workers’ recent analogous use of 5 mol % [Ph_2_PCs­(18-crown-6)] (>99% conversion at room temperature, 10 min).^30^ In common with this latter species, compound **3** was ineffective for the hydrophosphination of less activated substates such as the internal alkene, *trans*-stilbene (entry 4) and 1-hexene (entry 5), which provided no evidence of reaction under the applied conditions.

**Table 2:**
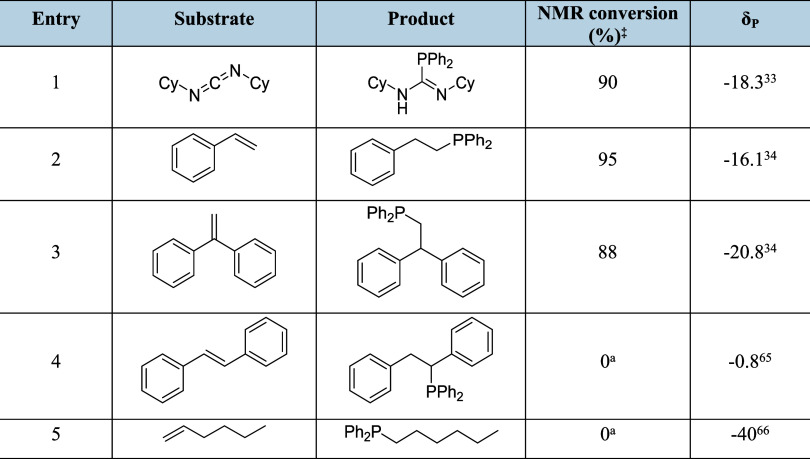
Intermolecular Hydrophosphination of CyN=C=NCy and Various Alkenes with Ph_2_PH and Catalyzed by 3^a^,[Table-fn t2fn2]

a benzene solution; reactions performed at a 0.13 mmol scale with 5 mol % **3**; 40 °C, 16 h.

‡ determined by ^31^P NMR spectroscopy versus a PPh_3_ standard.

In an initial attempt to broaden the scope of BDI-supported phosphidocalcium reagents beyond the diphenylphosphide derivatives, **2** and **4**, compound **3** was reacted with *t*-Bu_2_PH. Although monitoring at room temperature evidenced limited conversion (ca. 25% after 2 weeks, see Figures S25–S27) to the species subsequently identified as compound **6** (*vide infra*), attempts enhance its formation at even mildly elevated temperature (40 °C) resulted in solution redistribution of the calcium hydride reagent to [(BDI)_2_Ca] and other intractable products.^67^ We have recently reported that **3** may also be employed as a partner in transmetalation reactions with a variety of diarylmercury and organo- and amidozinc reagents.^60,61,68–70^ Although the mercury-based systems allow direct access to the corresponding arylcalcium species, presumably due to the instability of the hydridomercury byproducts, intermetallic ligand transfer from zinc occurs via the intermediacy of identifiable hydridoalkyl- and hydridoamidozincate intermediates. We, thus, assessed the use of an analogous transmetallative protocol for the synthesis of further calcium phosphide derivatives.

Reminiscent of our earlier obervations of diarylmercurials, reactions were performed between **3** and half molar equivalents of Hg­(PR_2_)_2_ (where R = *t*-Bu and SiMe_3_). In both cases a gradual effervescence and the deposition of a gray metallic precipitate, assumed to be elemental mercury, was observed (Scheme 2). Filtration and crystallization of the respective reaction products from benzene and hexane provided good yields (*ca*. 65%) of [(BDI)­Ca­(μ_2_–H)­(μ_2_-PR_2_)]_2_, compounds **6** (R = *t*-Bu) and **7** (R = SiMe_3_), as colorless crystals. While **6** presented a single ^31^P­{^1^H} resonance at δ 63.5 ppm that was at significantly lower field than the comparable data provided by either of the diphenylphosphide derivatives, compounds **2** or **4**, the ^31^P nucleus of the bis-trimethylsilyl-substituted phosphide (**7**) resonated at significantly higher field (δ −250.1 ppm). Despite these contrasting data, both of the corresponding ^1^H NMR spectra displayed resonances consistent with single unique BDI ligand environments characterized by singlet γ-methine signals at δ 4.78 (**6**) and 4.76 ppm (**7**). The spectra of both compounds were further characterized by doublet signals at 50% of this relative intensity at δ 4.92 (**6**, ^2^ *J*_HP_ = 48.1 Hz) and 4.77 ppm (**7**, ^2^ *J*_HP_ = 46.4 Hz) that were, thus, assigned as μ_2_–Ca–H-Ca bridging hydride environments. In further mitigation of these assignments, the phosphorus-bound *tert*-butyl and SiMe_3_ protons were respectively observed to resonate as upfield doublet signals (**6**; δ 0.76 ppm, ^3^ *J*_HP_ = 12.5 Hz: **7**; δ 0.53 ppm (6H),^3^ *J*_HP_ = 4.1 Hz and −0.22 ppm (12H), ^3^ *J*_HP_ = 4.2 Hz), each with a total relative integration of 18H.

**Scheme 2:**
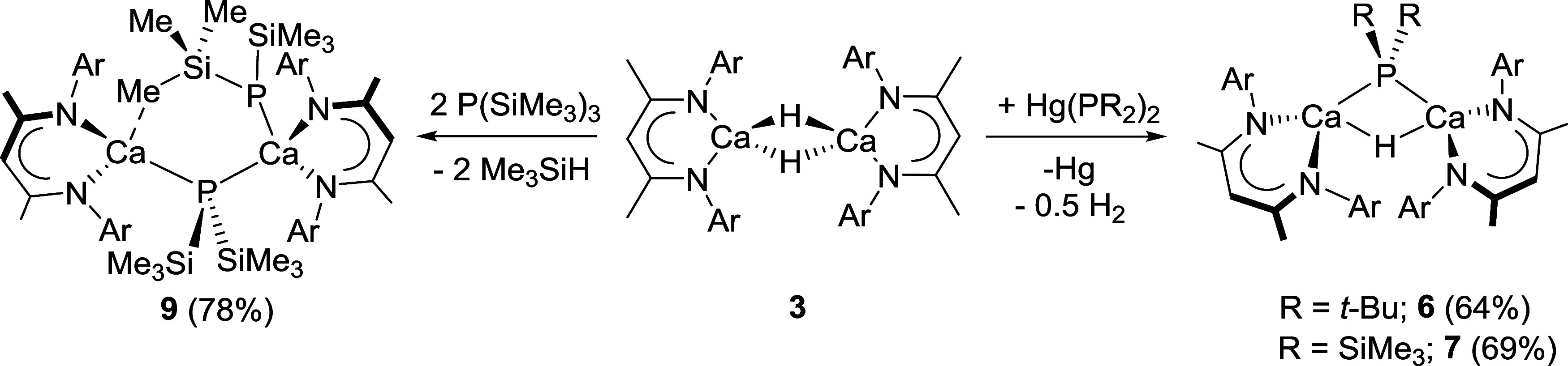
Synthesis of Compounds **6**, **7** and **9** (Values in Parentheses Indicate the Isolated Yields)

This latter deduction was again confirmed by recourse to X-ray diffraction experiments that established both **6** and **7** as dimeric phosphidocalcium species, each with two [(BDI)­Ca] units connected through a combination of μ_2_–Ca–P-Ca and μ_2_–Ca–H-Ca bridging interactions (Figure 3 with selected bond lengths and angles presented in Table 1). Despite the contrasting electronic environments of the phosphorus nuclei implied by their ^31^P NMR spectra, the structures of **6** and **7** are closely comparable with negligible apparent impact on the observed Ca–P bond lengths, either across both hydride-bridged compounds [Ca1–P1 2.9791(8) (**6**), 2.9676(9) (**7**); Ca2–P1 2.9559(8) (**6**), 2.9245(9) Å (**7**)] or in comparison to the corresponding metrics provided by the μ_2_–P–Ca-P unit of compound **4** (Table 1). In contrast, the small size of the bridging hydride ligand imposes a much more acute Ca1–P1–Ca2 angle [78.52(2) (**6**), 80.40(2)° (**7**)] than was observed in **4** [124.27(2)°] where the secondary bridging interaction was enabled by a combination of Ca–P and Ca-η^6^-C_6_H_5_P π-arene interactions (*vide supra*). Although the structures of both compounds display a high degree of commonality, the relative disposition of the bridging [P*t*-Bu_2_]^−^ and [P­(SiMe_3_)_2_]^−^ anions provide a notable point of contrast. The individual *tert*-butyl groups of **6** are similarly situated to either side of the approximate plane of symmetry defined by Ca1, P1 and Ca2, such that the calculated τ’_4_ value of 0.80 is indicative of a geometry at phosphorus that approaches tetrahedral.^71^ In contrast, the corresponding metric applied to the phosphorus center of **7** (τ′_4_ = 0.63) is reflective of a geometry that is better described as a trigonal pyramid such that a basal plane is defined by Ca1, Ca2 and Si2 with the Si1-containing silyl substituent occupying the apical position. This asymmetry, imposed by a close agostic-type interaction between the C32 methyl substituent (Figure 2b), was also apparent in the discriminated SiMe_3_ doublet signals in the ^1^H NMR data reported above. Although the instability of compound **7** mitigated against further solution analysis at more elevated temperatures, we ascribe this observation to the likely maintenance of such interactions relative to the NMR time scale, and the onset of a dynamic diastereotopicity resulting from alternating C–H···Ca1/Ca2 engagements, in the manner of the clapper of a bell, of both SiMe_3_ substituents to either side of the Ca1–P1–Ca2 plane.

**Figure 3:**
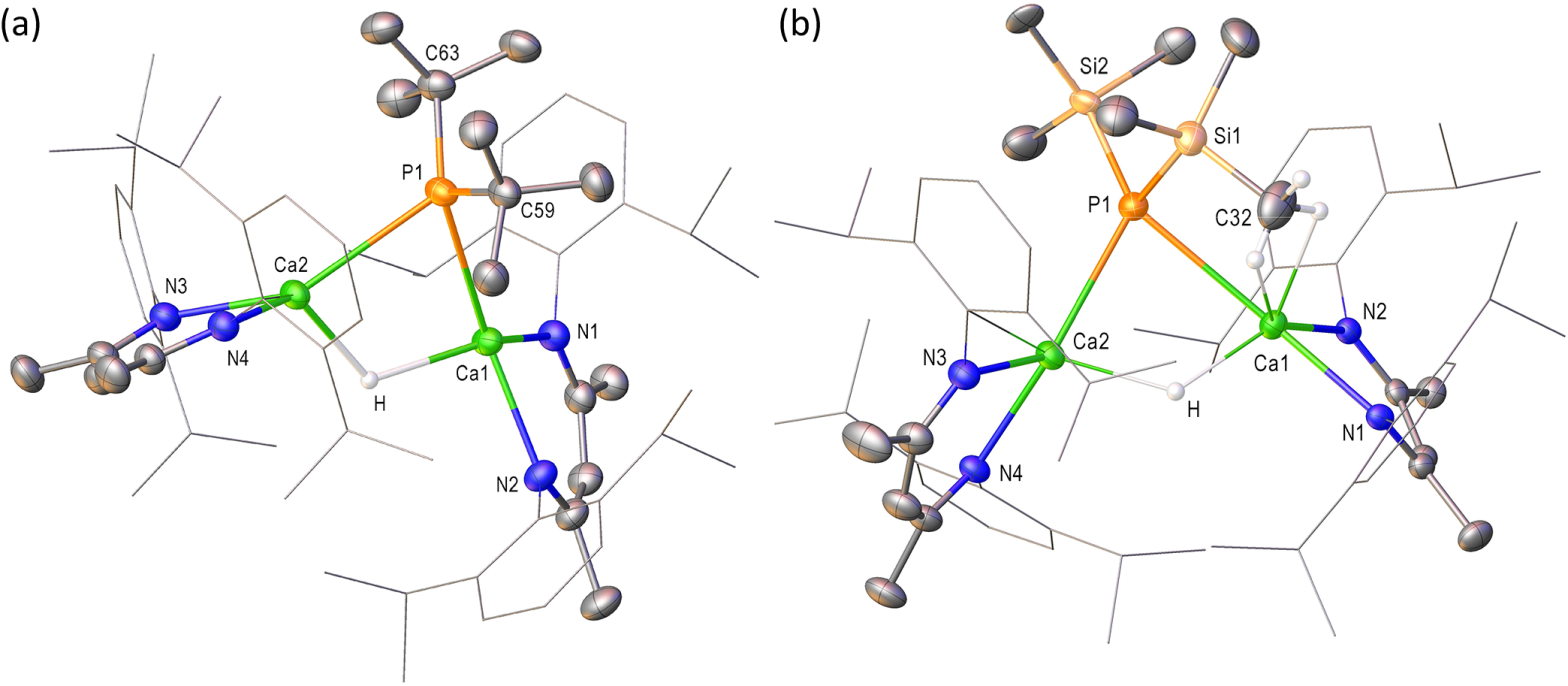
Displacement ellipsoid plots (30% probability) of (a) compound **6**, (b) compound **7**. Occluded molecules of benzene (**6**) or hexane (**7**) and disordered atoms have been removed for clarity as have hydrogen atoms, apart from metal-bound hydrides. Wireframe view has been employed for the Dipp substituents of both compounds and the Si-bound methyl groups of **7**, also for visual ease.

The apparently more compact structures of **6** and **7** also impact on the reactivity of the remaining hydride functionalities. Attempts to enforce reaction of **6** and **7** with further equivalents of Hg­(PR_2_)_2_ (R = *t*-Bu, SiMe_3_) were unsuccessful, albeit compound **9** ([(BDI)­CaP­(SiMe_3_)_2_]_2_, δ_P_ = – 265 ppm, *vide infra*) could be identified by ^31^P NMR spectroscopy as a minor component of the complex mix of products formed from **7** and Hg­{P­(SiMe_3_)_2_}_2_. Although reactions of both compounds with single molar equivalents of protic reagents such as terminal alkynes were similarly unsuccessful, monitoring of a reaction of **6** with ZnMe_2_ by ^1^H NMR spectroscopy did allow for the identification of the previously reported [(BDI)­CaMe]_2_,^68^ which was formed as a component of a mixture of otherwise unidentifiable species. Some further insight into the nature of this reaction was provided by the isolation of several single crystals of the dimeric and centrosymmetric zincate species, [(BDI)­Ca­(μ-CH_3_)­(μ-P*t*-Bu_2_)­Zn­(μ-H)]_2_ (**8**), which was identified solely by a single crystal X-ray diffraction analysis. Although the resultant structure, which is presented in Figure 4a with selected bond length and angle data in the figure caption, bears a topological resemblance to previously reported species resulting from reactions of **3** with organo- and amidozinc reagents,^68–70^ it will not be discussed further due to the arbitrary nature of its synthesis.

**Figure 4:**
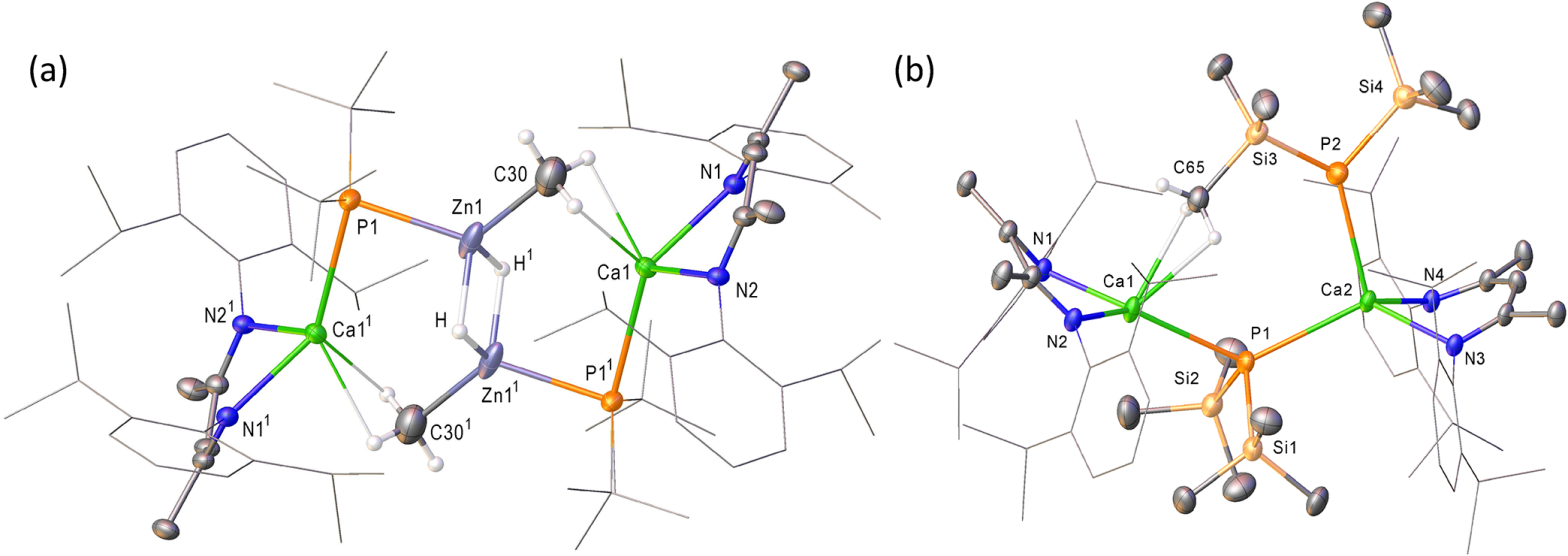
Displacement ellipsoid plots (30% probability) of (a) the Ca1-containing molecule of compound **8**, (b) compound **9**. Occluded molecules of benzene (**8**) and hexane (**9**) solvent and disordered atoms have been removed for clarity as have hydrogen atoms, apart from metal-bound hydrides and those attached to C30 in **8** and C65 in **9**. Also, for visual ease, wireframe view has been employed for the Dipp substituents of both compounds and the *t*-butyl groups of **8**. Symmetry operations to generate equivalent atoms (**8**); ^1^1–*x*, 1–*y*, 1–*z*; ^2^2–*x*, 1*–y*, 2*–z*; ^3^1–*x*, 1– *y*, 2–*z*. Selected bond lengths (Å) and angles (°) (**8**): Zn1–P1 2.3578(6), Zn1–C30 2.194(4), Ca1–P1^1^ 2.8842(7), Ca1–N1 2.3530(17), Ca1–N2 2.3333(17), Zn1–P1–Ca1^1^ 85.83(2), N2–Ca1–N1 83.70(6).

In an attempt to circumvent the lack of discriminating reactivity of **7** toward Hg­{P­(SiMe_3_)_2_}_2_, a sample of compound **3** was heated at 40 °C for 16 h with 2 mol equiv P­(SiMe_3_)_3_ (Scheme 2). Monitoring of the reaction by ^31^P and ^1^H NMR spectroscopy evidenced the complete disappearance of the hydride environments of **3**, the formation of Me_3_SiH and the generation of a predominant β-diketiminato species (**9**) characterized by a single ^31^P NMR environment which resonated at δ_P_ −265.2 ppm. Removal of volatiles, crystallization from hexane and subsequent X-ray diffraction analysis confirmed the origin of these observations (Figure 4b and Table 1).

Like **4**, compound **9** is a further example of a dimeric calcium phosphide. The primary mode of dimer propagation in **9** results from a single bridging μ_2_–Ca1–P1-Ca2 interaction, the angle at which [128.96(6)°] is more reminsicent of that observed in **4**. Also in a similar manner to **4** the P2 center of the remaining phosphide anion interacts as a terminal ligand with Ca2 [P2–Ca2 2.8320(17) Å] and any engagement with Ca1 is limited to a further agostic-type contact with the C65-containing silylmethyl substituent. Although further studies by low temperature ^1^H NMR spectroscopy allowed the identification of at least two dynamic processes (Δ*G* ^‡^ = *ca*. 48 and 37 kJ mol^–1^) providing evidence that this interaction is also maintained in solution, any process of molecular rearrangement is evidently very labile and more definitive assignment is, thus, imprudent.

Although, like **4**, compound **9** also holds potential as a source of the P­(SiMe_3_)_2_ anion, all attempts to study its reactivity were unsuccessful. In behavior which we suggest as most likely due to the competitive lability of the P–Si bonds, all reactions of **9** with reducible substrates such as styrene and carbodiimides only resulted in the formation of intractable mixtures of unidentifiable species.

## Conclusion

Whereas the β-diketiminate derivative, [(BDI)­Ca-μ-H]_2_, reacts with Ph_2_PH to provide the dimeric calcium phosphide, [(BDI)­CaPPh_2_]_2_, access to derivatives of less acidic or more sterically encumbered phosphines requires the adoption of alternative synthetic methods. Transmetalation with mercury phosphides results in the generation of dimeric hydridocalcium phosphides, [(BDI)­Ca­(μ_2_–H)­(μ_2_-PR_2_)]_2_ (R = *t*-Bu, SiMe_3_), in which the remaining hydride functions are resistant to further exchange. In contrast, the bis-trimethylsilylphosphide, [(BDI)­CaP­(SiMe_3_)_2_]_2_, forms selectively by elimination of Me_3_SiH and through the reaction of [(BDI)­Ca-μ-H]_2_ and P­(SiMe_3_)_3_ at 40 °C, albeit the lability of the resultant compound appears to limit its further utility as a reactive source of the phosphide anion.

## Experimental Section

### General Considerations

All manipulations were carried out using standard Schlenk line and glovebox techniques under an inert atmosphere of argon. NMR experiments were conducted in J-Young’s NMR tubes and prepared in a glovebox. NMR spectra were recorded on a Bruker BioSpin GmbH spectrometer operating at 400.13 MHz (^1^H), 100.62 MHz (^13^C) and 161.98 MHz (^31^P). Elemental analyses were performed at Elemental Microanalysis Ltd., Okehampton, Devon, U.K. or by the Elemental Analysis Services Team at London Metropolitan University. Solvents were dried by passage through a commercially available solvent purification system and stored under argon in ampules over 4 Å molecular sieves. C_6_D_6_ and C_7_D_8_ were purchased from Merck, dried over potassium, distilled and stored over molecular sieves. HgCl_2_ was purchased from Merck and sublimed prior to use. ZnMe_2_ was purchased from Merck as a standard solution, transferred to a J-Young’s ampule and used without further purification. CyNCNCy was purchased from Merck and dried under reduced pressure overnight prior to use. Ph_2_PH,^72^ HP­(^*t*^Bu)_2_,^73^ HP­(SiMe_3_)_2_,^74^ P­(SiMe_3_)_3_,^75^ Hg­(PR_2_)_2_ R = *t*-Bu,^76^ SiMe_3_,^77^ and [(BDI)­CaH]_2_ (BDI = HC­{(Me)­CN-2,6-*i-*Pr_2_C_6_H_3_}_2_) were prepared via literature procedures.^54^ Note: All mercury reagents should be treated as highly toxic and residues resulting from their use must be disposed of to a dedicated mercury waste container.

### Synthesis of [(BDI)­Ca­(PPh_2_)]_2_ (**4**)

[(BDI)­CaH]_2_ (30 mg, 0.033 mmol) was dissolved in C_6_D_6_ (0.6 cm^3^) inside a J-Young’s NMR tube and Ph_2_PH (11.4 μL, 0.065 mmol) was added via Eppendorf pipet. A red coloration was observed alongside vigorous effervescence, which quickly subsided, marking complete conversion. The volatiles were removed *in vacuo* and the product was crystallized from hexane. Yield: 34.6 mg, 82%. Anal. Calc. for C_82_H_102_N_4_P_2_Ca_2_: C, 76.60; H, 8.00; N, 4.36. Found: C, 74.72; H, 7.84; N, 3.93. ^1^H NMR (C_6_D_6_): δ = 7.22 (br s, Ar–H, 3H), 7.11–7.06 (m, Ar–H, 7H), 6.89 (br t, Ca–P-*meta*–Ar-H, ^3^ *J*_HH_ = 6.87 Hz, 4H), 6.79 (br t, CaP-*para*–Ar-H, 2H), 4.87 (s, HCCN, 1H), 2.99 (hept, HC­(CH_3_)_2_, ^3^ *J*_HH_ = 6.70 Hz, 4H), 1.55 (s, NCCH_3_,6H), 1.04 (d, HC­(CH_3_)_2_, ^3^ *J*_HH_ = 6.68 Hz, 12H), 0.95 (br s, HC­(CH_3_)_2_, 12H). ^13^C­{^1^H} NMR (C_6_D_6_): δ = 166.9 (NCCH_3_), 146.8 (*i-* ^Dipp^Ar–C), 141.6 (^Dipp^Ar–C), 132.9 (Ar–C), 129.1 (Ca–P-*m*eta–Ar-C), 124.6 (Ca–P-*para*–Ar-C), 123.9 (*p-* ^Dipp^Ar–C), 93.8 (HCCN), 28.6 (HC­(CH_3_)_2_), 25.0 (NCCH_3_), 24.8 (HC­(CH_3_)_2_), 23.8 (HC­(CH_3_)_2_). ^31^P­{^1^H} NMR (C_6_D_6_): δ = −16.93 (s).

### Synthesis of [(BDI)­Ca­{NCy}_2_CPPh_2_)] (**5**)

Dicyclohexylcarbodiimide (27 mg,0.13 mmol) was added to a benzene solution of [(BDI)­CaPPh_2_]_2_ (84 mg, 0.065 mmol), resulting in an instant color change from red to colorless. The volatiles were removed under reduced pressure and the crude reaction mixture was dissolved in hexane and colorless crystals of **5** were grown at −35 °C. Yield: 54 mg, 97%. Anal. Calc. for C_54_H_73_N_4_P_1_Ca_1_: C, 76.37; H, 8.66; N, 6.60. Found: C, 75.90; H, 8.63; N, 6.46. ^1^H NMR (C_6_D_6_): δ = 7.66 (t, NPPh_2_-*ortho*–Ar-H, ^3^ *J*_HH_ = 7.36 Hz, 4H), 7.18 (br s, Ar–H, 2H), 7.13–7.10 (m, Ar–H, 8H), 7.02 (t, ^3^ *J*_HH_ = 7.34 Hz, 2H), 4.85 (s, HCCN, 1H), 3.73 (br s, NCH­(CH_2_CH_2_}_2_CH_2_, 2H), 3.27 (hept., HC­(CH_3_)_2_, ^3^ *J*_HH_ = 6.86 Hz, 4H), 1.73 (s, NCCH_3_, 6H), 1.41 (m, NCH­(CH_a_ H_b_CH_a_ H_b_}_2_CH_2_, 8H), 1.36 (d, HC­(CH_3_)_2_, ^3^ *J*_HH_ = 6.86 Hz, 12H), 1.27 (d, HC­(CH_3_)_2_, ^3^ *J*_HH_ = 6.86 Hz, 12H), 0.98 (m, NCH­(CH_a_H_b_CH_a_H_b_}_2_CH_2_, 6H), 0.82 (NCH­(CH_a_H_b_CH_a_H_b_}_2_CH_2_, 4H). ^13^C­{^1^H} NMR (C_6_D_6_): δ = 168.8 (d, *Cy*NC­(PPh_2_)­NCy, ^1^ *J*_CP_ = 56.6 Hz), 165.9 (NCCH_3_), 146.7 (*i-* ^Dipp^Ar–C), 141.0 (^Dipp^Ar–C), 136.9 (d, NPPh_2_-*ipso*–Ar-C, ^1^ *J*_CP_ = 19.5 Hz), 132.7 (d, NPPh_2_-*ortho*–Ar-C, ^2^ *J*_CP_ = 19.1 Hz), 128.1 (d, NPPh_2_-*meta*–Ar-C, *J*_CP_ = 5.84 Hz), 124.2 (NPPh_2_-*para*–Ar-C), 123.5 (^Dipp^Ar–C), 90.8 (HCCN), 56.2 (d, NCH­(CH_2_CH_2_}_2_CH_2_, *J*_CP_ = 20.5 Hz), 37.0 (NCH­(CH_a_H_b_CH_a_H_b_}_2_CH_2_), 28.5 (HC­(CH_3_)_2_), 25.8 (NCH­(CH_a_H_b_ CH_a_H_b_}_2_CH_2_), 25.5 (HC­(CH_3_)_2_), 24.6 (NCCH_3_), 24.0 (HC­(CH_3_)_2_). ^31^P­{^1^H} NMR (C_6_D_6_): δ = −19.07 (s).

### General Procedure for Catalytic Hydrophosphination

Ph_2_PH (22.62 μL, 0.13 mmol) and the alkene substrate (0.13 mmol) were introduced into a J-Young’s NMR tube followed by C_6_D_6_ (0.6 cm^3^) and a catalytic amount of [(BDI)­CaH]_2_ (5 mol %, 6 mg, 0.0065 mmol). The sample was set heating at 40 °C and left for 16 h. After such time, PPh_3_ (4 mg) was added as an internal standard and yields were determined spectroscopically implementing a 30 s relaxation delay.

### Synthesis of [(BDI)­Ca­(H)­P*t*-Bu_2_Ca­(BDI)] (**6**)

[(BDI)­CaH] (30 mg, 0.033 mmol) and Hg­(P*t*-Bu_2_)_2_ (8 mg, 0.016 mmol) were introduced into a J-Young’s NMR tube and dissolved in *d*_8_-toluene (ca. 0.6 cm^3^). Gradual effervescence was observed alongside a gray coloration. The solution was left for 16 h, after which a a gray precipitate was observed at the bottom of the NMR tube, presumed to be Hg metal. The solution was filtered into an ampule and concentrated, before being redissolved in benzene and transferred to a vial. The solvent was slowly evaporated until the formation of colorless crystals was observed, which were isolated and dried *in vacuo*. Yield: 22.3 mg, 64%. Anal. Calc. for C_66_H_101_N_4_P_1_Ca_2_: C, 74.67; H, 9.59; N, 5.28. Found: C, 82.9; H, 9.30; N, 4.46. ^1^H NMR (C_6_D_6_): δ = 7.10 (m, ^Dipp^Ar–H, 12H), 4.92 (d, Ca-μ_2_-H-Ca, *J*_*HP*_ = 48.1 Hz, 1H), 4.78 (s, HCCN, 2H), 3.22 (br hept., HC­(CH_3_)_2_, ^3^ *J*_HH_ = 6.67 Hz, 8H), 1.62 (s, NCCH_3_, 12H), 1.24 (d, HC­(CH_3_)_2_, ^3^ *J*_HH_ = 7.17 Hz, 48H), 0.76 (d, PC­(CH_3_)_3_, ^3^ *J*_HP_ = 12.49 Hz, 18H). ^13^C­{^1^H} NMR (C_6_D_6_): δ = 166.0 (NCCH_3_), 146.1 (*i-* ^Dipp^Ar–C), 141.8 (^Dipp^Ar–C), 124.5 (^Dipp^Ar–C), 123.9 (^Dipp^Ar–C), 94.5 (HCCN), 35.6 (d, PC­(CH_3_)_2_, ^2^ *J*_CP_ = 10.2 Hz), 31.7 (d, PC­(CH_3_)_2_, ^1^ *J*_CP_ = 15.1 Hz), 28.5 (HC­(CH_3_)_2_), 25.4 (HC­(CH_3_)_2_), 24.5 (NCCH_3_), 23.9 (HC­(CH_3_)_2_). ^31^P­{^1^H} NMR (C_6_D_6_): δ = 63.47 (s). **Note:** Attempts to prepare **6** from *t*-Bu_2_PH were unsuccessful and when heated resulted in redistribution and BDI-H formation. Furthermore, reaction of **6** with another equivalent of Hg­(P*t*-Bu_2_)_2_ did not proceed. Similarly, reactions with protic sources e.g., alkynes, resulted in displacement of both bridging moieties.

### Synthesis of [(BDI)­Ca­(H)­P­(SiMe_3_) _2_Ca­(BDI)] (**7**)

[(BDI)­CaH] (25.3 mg, 0.028 mmol) and Hg­{P­(SiMe_3_)_2_}_2_ (7.65 mg, 0.014 mmol) were introduced into a J-Young’s NMR tube and dissolved in *d*_8_-toluene (ca. 0.6 cm^3^). Gradual effervescence was observed alongside a gray coloration. The solution was left for 16 h, after which a gray precipitate was observed at the bottom of the NMR tube, presumed to be Hg metal. The solution was filtered into an ampule and concentrated, before being recrystallized from hexane at −35 °C. The colorless crystals were isolated, washed with cold hexane and dried under reduced pressure. Yield: 21.3 mg, 69%. Anal. Calc. for C_64_H_1007_N_4_P_1_Si_2_Ca_2_: C, 69.89; H, 9.81; N, 5.09. Found: C, 69.65; H, 9.64; N, 4.83. ^1^H NMR (C_7_D_8_): δ = 4.77 (d, Ca-μ_2_-H-Ca, *J*_HP_ = 46.43 Hz, 1H), 4.76 (s, HCCN, 2H), 3.23 (hept., HC­(CH_3_)_2_, ^3^ *J*_HH_ = 6.85 Hz, 8H), 1.60 (s, NCCH_3_, 12H), 1.24 (d, HC­(CH_3_)_2_, ^3^ *J*_HH_ = 6.85 Hz, 48H), 0.53 (d, Si­(CH_3_)_2_CH_3_, *J* = 4.08 Hz, 6H), −0.22 (d, Si­(CH_3_)_2_CH_3_, *J* = 4.21 Hz, 12H). ^13^C­{^1^H} NMR (C_7_D_8_): δ = 166.0 (NCCH_3_), 145.7 (*i-* ^Dipp^Ar–C), 141.5 (^Dipp^Ar–C), 123.8 (^Dipp^Ar–C), 94.5 (HCCN), 28.3 (HC­(CH_3_)_2_), 23.9 (HC­(CH_3_)_2_), 22.7 (HC­(CH_3_)_2_), 7.5 (d, Si­(CH_3_)_2_ CH_3_, *J* = 10.4 Hz), 6.29 (d, Si­(CH_3_)_2_CH_3_, *J* = 9.6 Hz). ^31^P­{^1^H} NMR (C_7_D_8_): δ = −250.05. **Note:** Adding Hg­{P­(SiMe_3_)_2_}_2_ to [(BDI)­Ca­(H)­P*t*-Bu_2_Ca­(BDI)] (**6**) also generates **7**. Alongside the generation of [(BDI)­Ca­(H)­P­(SiMe_3_)_2_Ca­(BDI)] (**7**), a minor resonance was observed at δ_P_ −265 ppm, thought to be [(BDI)­CaP­(SiMe_3_)_2_]_2_ (**9**). The quantity of this species, however, could not be increased through the addition of more Hg­{P­(SiMe_3_)_2_}_2_.

### Synthesis of [(BDI)­Ca­(μ-CH_3_)­(μ-P*t-*Bu_2_)­Zn­(μ-H)]_2_ (**8**)

ZnMe_2_ (16.3 μL, 0.016 mmol) was added to a C_6_D_6_ solution of [(BDI)­Ca­(H)­P*t*-Bu_2_Ca­(BDI)] (**6**, 0.033 mmol), and monitored by NMR spectroscopy. The formation of (BDI)­CaMe and ^*t*^Bu_2_PH as well as minor unidentified species were observed spectroscopically (Figures S34 and S35).^78^ Repeating this reaction in a vial within the glovebox and placing it upon the glovebox freezer, resulted in a few crystals confirmed by X-ray diffraction to be [(BDI)­Ca­(μ-CH_3_)­(μ-P*t*-Bu_2_)­Zn­(μ-H)]_2_ (**8**). This compound could not be isolated exclusively for spectroscopic analysis.

### Synthesis of [(BDI)­CaP­(SiMe_3_)_2_]_2_ (**9**)

P­(SiMe_3_)_3_ (18.94 μL, 0.065 mmol) was added to a J Young’s tube containing [(BDI)­CaH]_2_ (30 mg, 0.033 mmol) dissolved in C_6_D_6_ (0.6 cm^3^) and heated at 40 °C for 16 h. The formation of **9** and Me_3_SiH^79^ were clearly visible by ^31^P and ^1^H NMR spectroscopy (Figures S41 and S42). Volatiles were removed under reduced pressure and **9** was crystallized from hexane, affording colorless crystals. Yield: 32.9 mg, 78%. Anal. Calc. for C_70_H_124_N_4_P_2_Si_4_Ca_2_: C, 65.88; H, 9.79; N, 4.39. Found: C, 66.35; H, 9.31; N, 4.86. ^1^H NMR (C_6_D_6_): δ = 7.17 (M, Ar–H, 6H), 4.76 (s, HCCN, 1H), 3.22 (hept., HC­(CH_3_)_2_, ^3^ *J*_HH_ = 6.71 Hz, 4H), 1.65 (s, NCCH_3_, 6H), 1.38 (d, HC­(CH_3_)_2_, ^3^ *J*_HH_ = 6.71 Hz, 12H), 1.21 (d, HC­(CH_3_)_2_, ^3^ *J*_HH_ = 6.71 Hz, 12H), 0.59 (d, SiMe_3_, *J* = 4.06 Hz, 18H). ^13^C­{^1^H} NMR (C_6_D_6_): δ = 165.4 (NCCH_3_), 146.7 (*i-* ^Dipp^Ar–C), 141.4 (^Dipp^Ar–C), 124.5 (^Dipp^Ar–C), 123.8 (^Dipp^Ar–C), 92.3 (HCCN), 27.9 (HC­(CH_3_)_2_), 25.4 (HC­(CH_3_)_2_), 24.7 (HC­(CH_3_)_2_), 7.6 (d, SiCH_3_, *J* = 10.6 Hz). ^31^P­{^1^H} NMR (C_6_D_6_): δ = −265.2.

## Supplementary Material



## Notes

NMR spectra and a summary of the X-ray diffraction experiments (PDF)
