# Supplementary material for: Molecular Calcium Phosphides and Mixed Phosphide Hydrides
Source: Inorg Chem. 2025 Dec 24;65(1):920–9. doi: 10.1021/acs.inorgchem.5c05335 (PMC12801296; doi:10.1021/acs.inorgchem.5c05335)
Supplement: Supplementary file 1 [file ic5c05335_si_001.pdf]

Supplementary Information

Molecular Calcium Phosphides and Mixed Phosphide Hydrides

Kyle G. Pearce,* Luke Teh, Andrew S. S. Wilson and Michael S. Hill*

Department of Chemistry, University of Bath, Claverton Down, Bath, BA2 7AY, UK

Email: kgp29@bath.ac.uk; msh27@bath.ac.uk

Contents

NMR Spectra

Figure S1-S6: NMR Spectra for **[(BDI)Ca(PPh$_2$)]$_2$ (4)** **S2**

Figure S7-S12: NMR Spectra for **[(BDI)Ca{NCy}$_2$C-PPh$_2$)] (5)** **S5**

Figure S13-S24: NMR Spectra for **Hydrophosphination Reactions** **S8**

Figure S25-S33: NMR Spectra for **[(BDI)Ca(H)P^{t}Bu$_2$Ca(BDI)] (6)** **S14**

Figure S34-S35: NMR Spectra for **[(BDI)Ca(μ-CH$_3$)(μ-P^{t}Bu$_2$)Zn(μ-H)]$_2$ (8)** **S18**

Figure S36-S40: NMR Spectra for **[(BDI)Ca(H)P(SiMe$_3$)$_2$Ca(BDI)] (7)** **S19**

Figure S41-S48: NMR Spectra for **[(BDI)CaP(SiMe$_3$)$_2$]$_2$ (9)** **S22**

Crystallographic Data **S26**

References **S30**

NMR Spectra

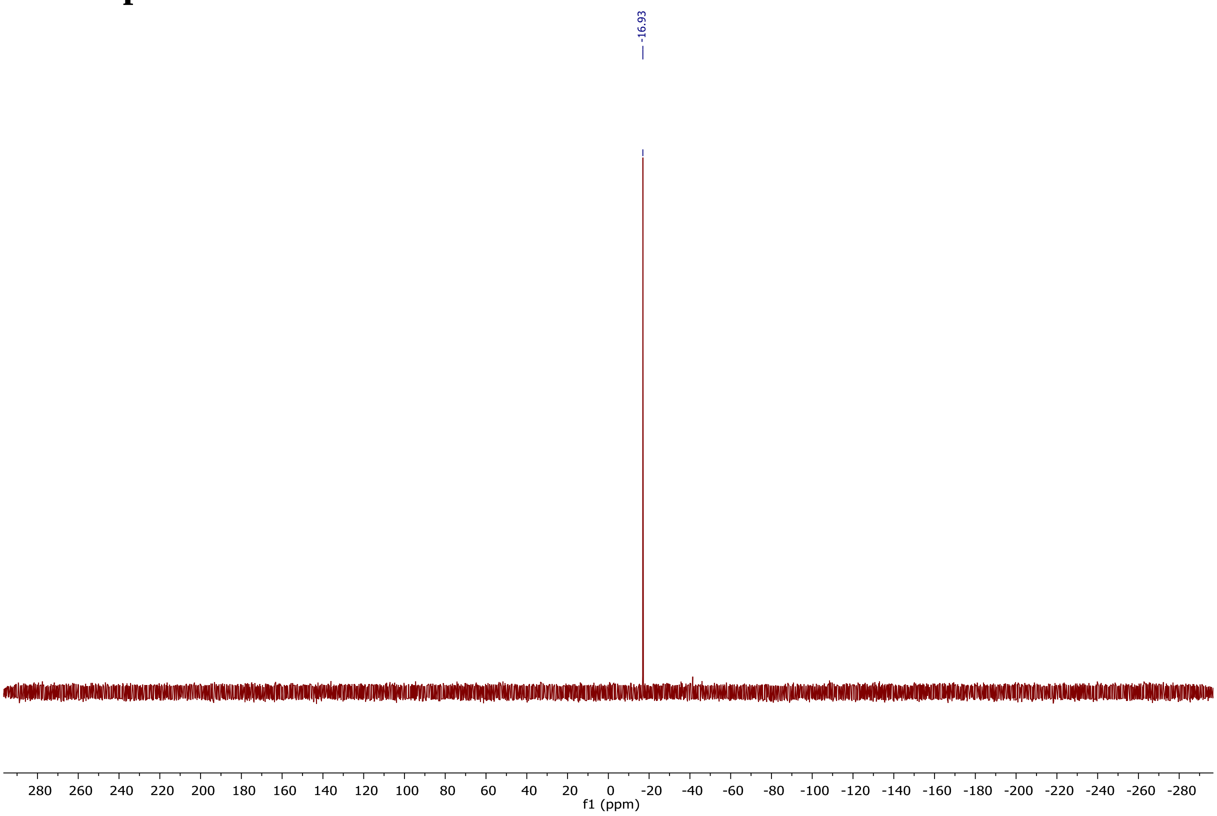

Figure S1. *^{31}P NMR Spectrum (C_6D_6, 298 K, 161.98 MHz) for* ***[(BDI)Ca(PPh₂)]₂ (4).***

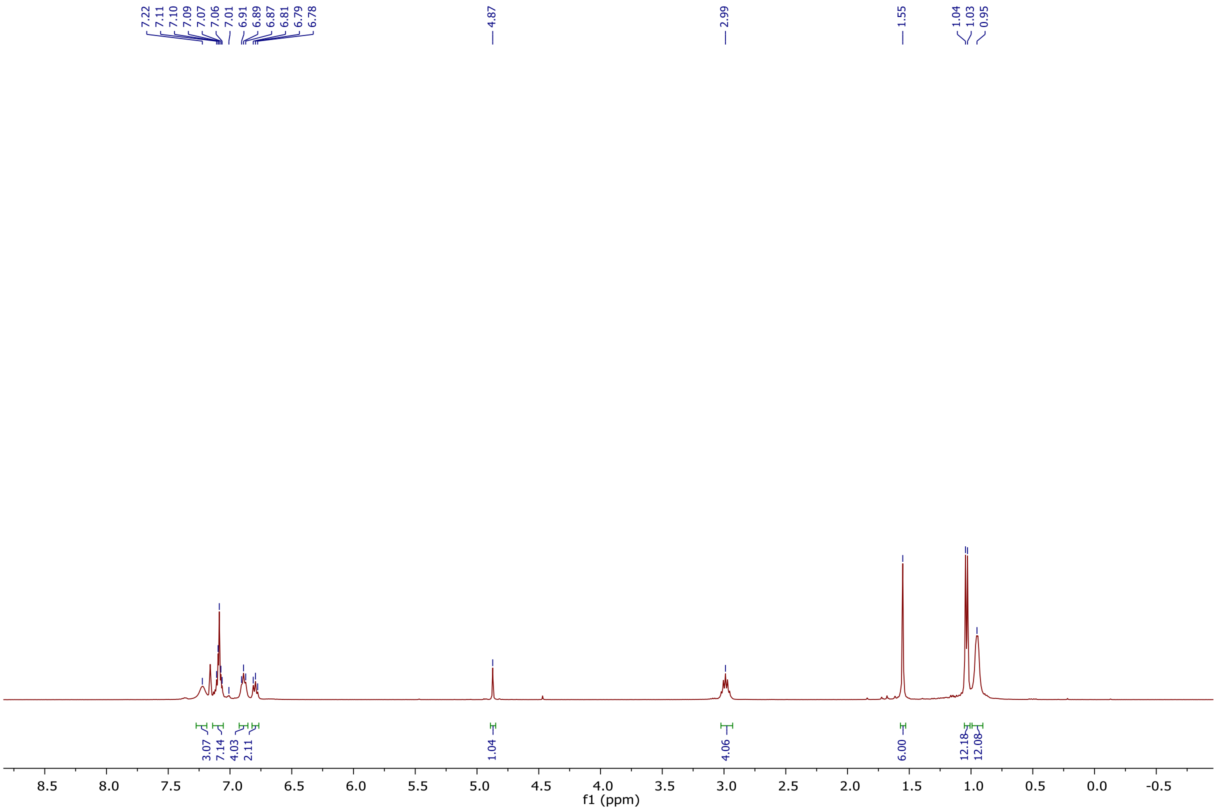

Figure S2. *^{1}H NMR Spectrum (C_6D_6, 298 K, 400.13 MHz) for* ***[(BDI)Ca(PPh_2)]$_2$ (4).***

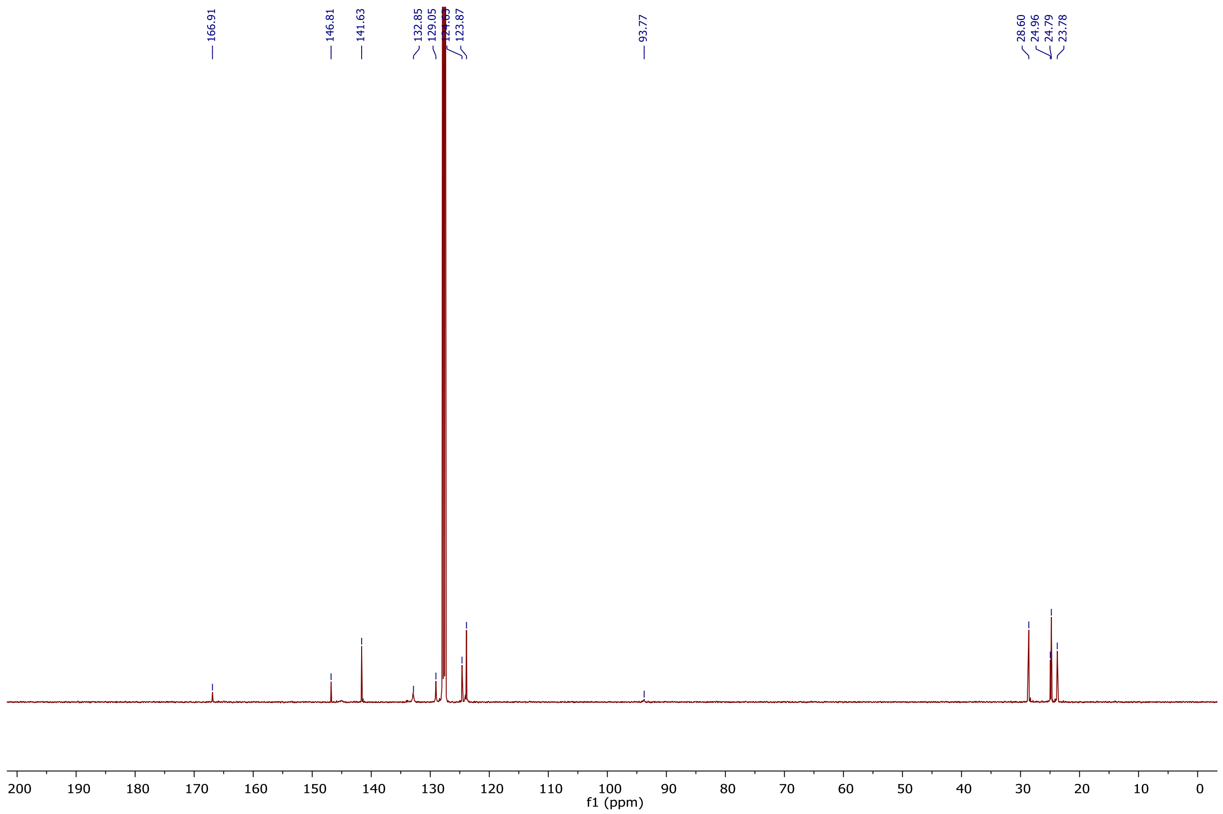

Figure S3. *^{13}C{^{1}H} NMR Spectrum (C_6D_6, 298 K, 100.62 MHz) for* ***[(BDI)Ca(PPh₂)]₂ (4).***

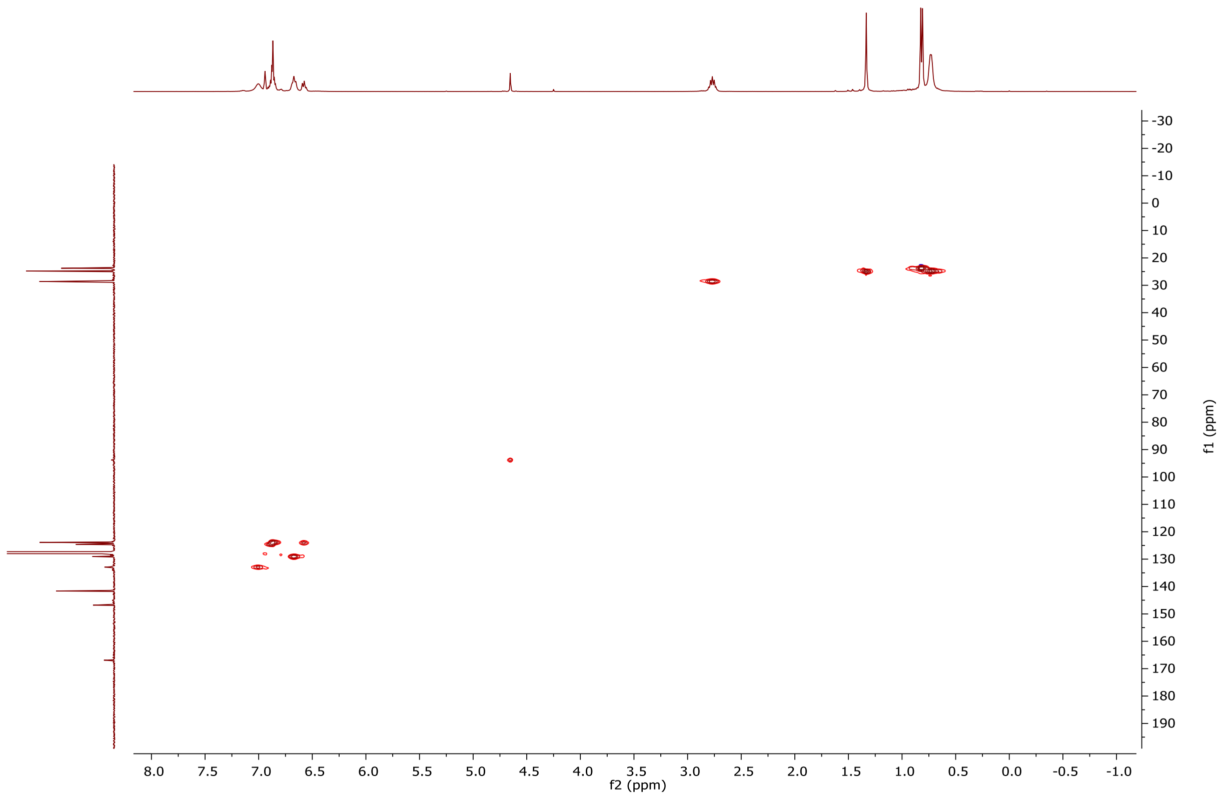

Figure S4*. ^{1}H-^{13}C HSQC trace (C_6D_6, 298 K, 400.13, 100.62 MHz) for* ***$[(BDI)Ca(PPh_2)]_2$ (4).***

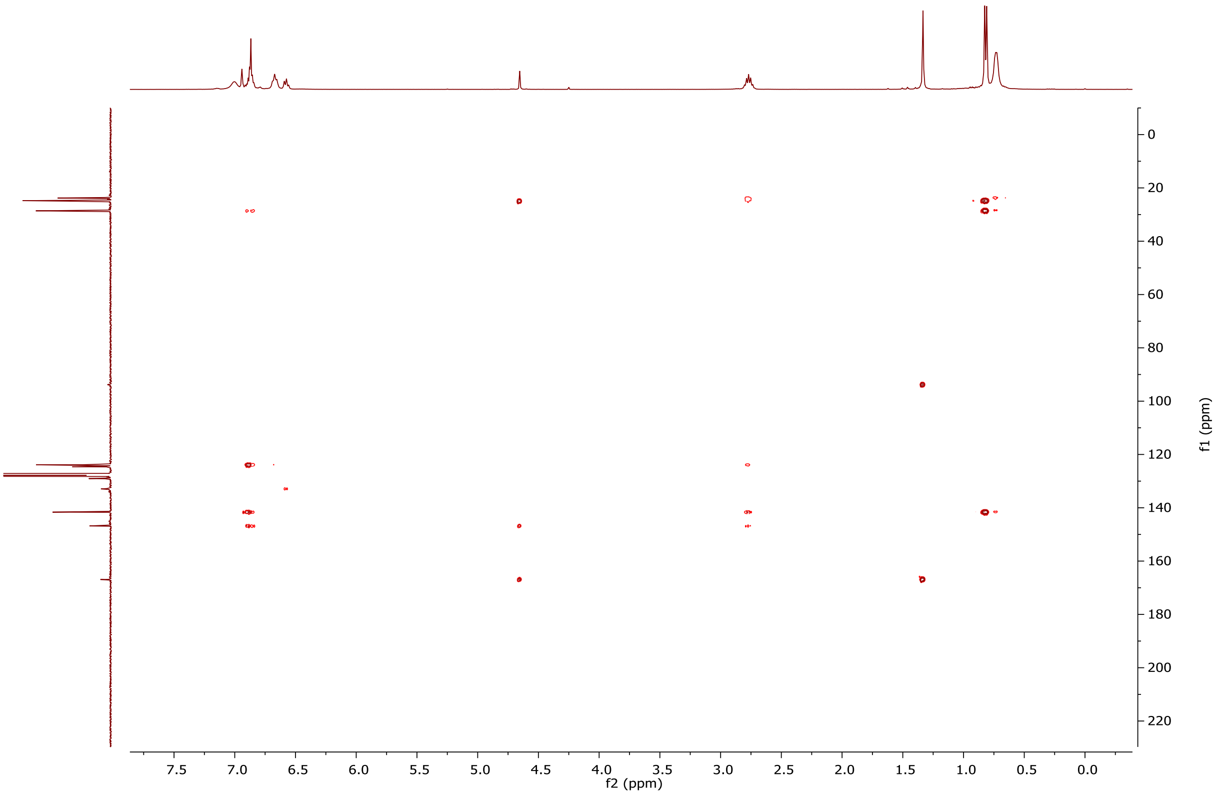

Figure S5. *^{1}H-^{13}C HMBC trace (C_6D_6, 298 K, 400.13, 100.62 MHz) for* ***[(BDI)Ca(PPh₂)]₂ (4).***

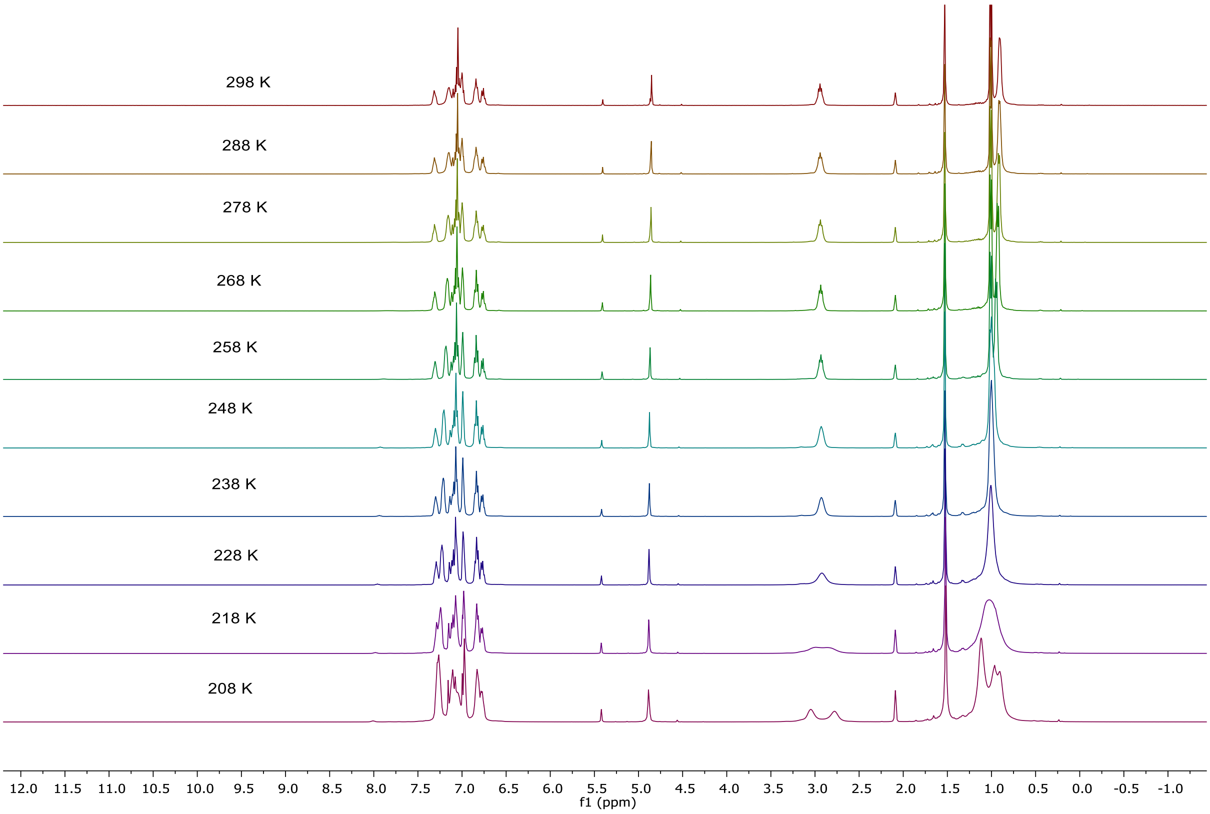

Figure S6. *Variable temperature ^{1}H NMR spectra (d_8-toluene, 400.13 MHz) of* ***$[(BDI)Ca(PPh_2)]_2$ (4).***

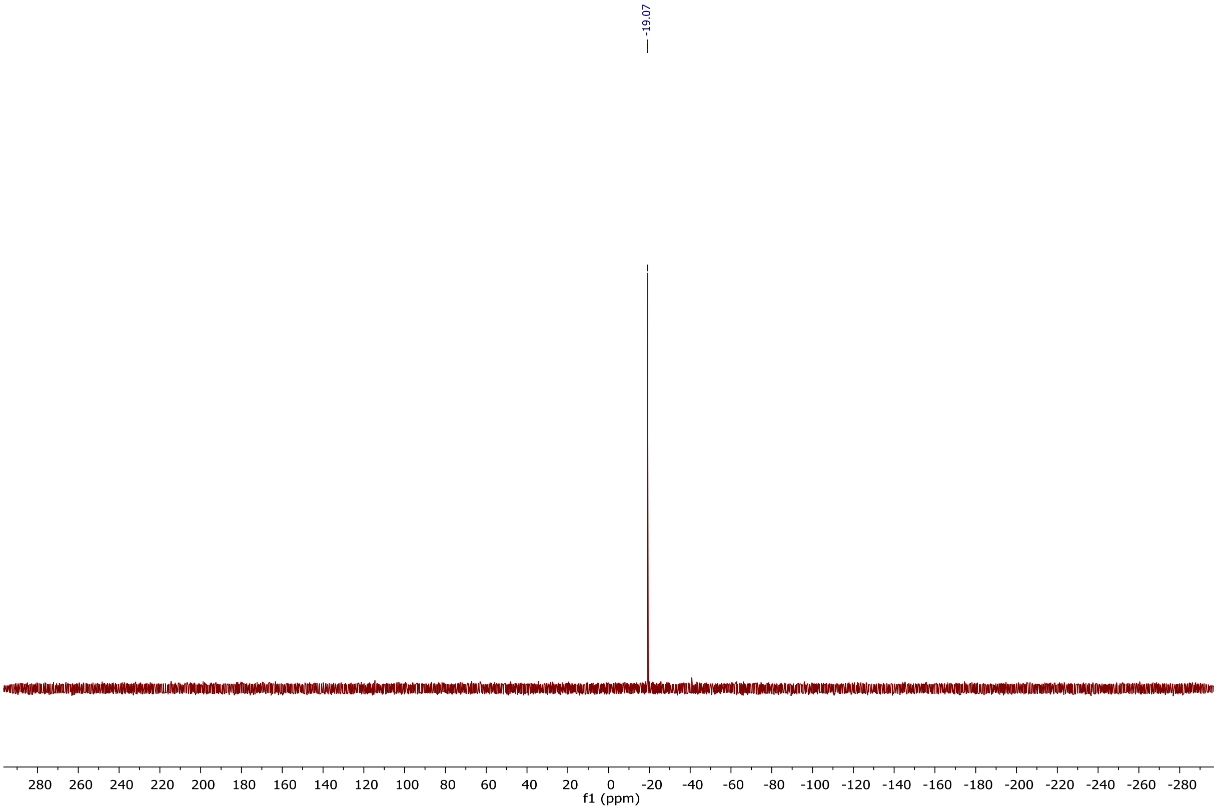

Figure S7. *$^{31}P\{^1H\}$ NMR Spectrum (C_6D_6, 298 K, 161.98 MHz) for* ***[(BDI)Ca{NCy}$_2$C-PPh$_2$)] (5).***

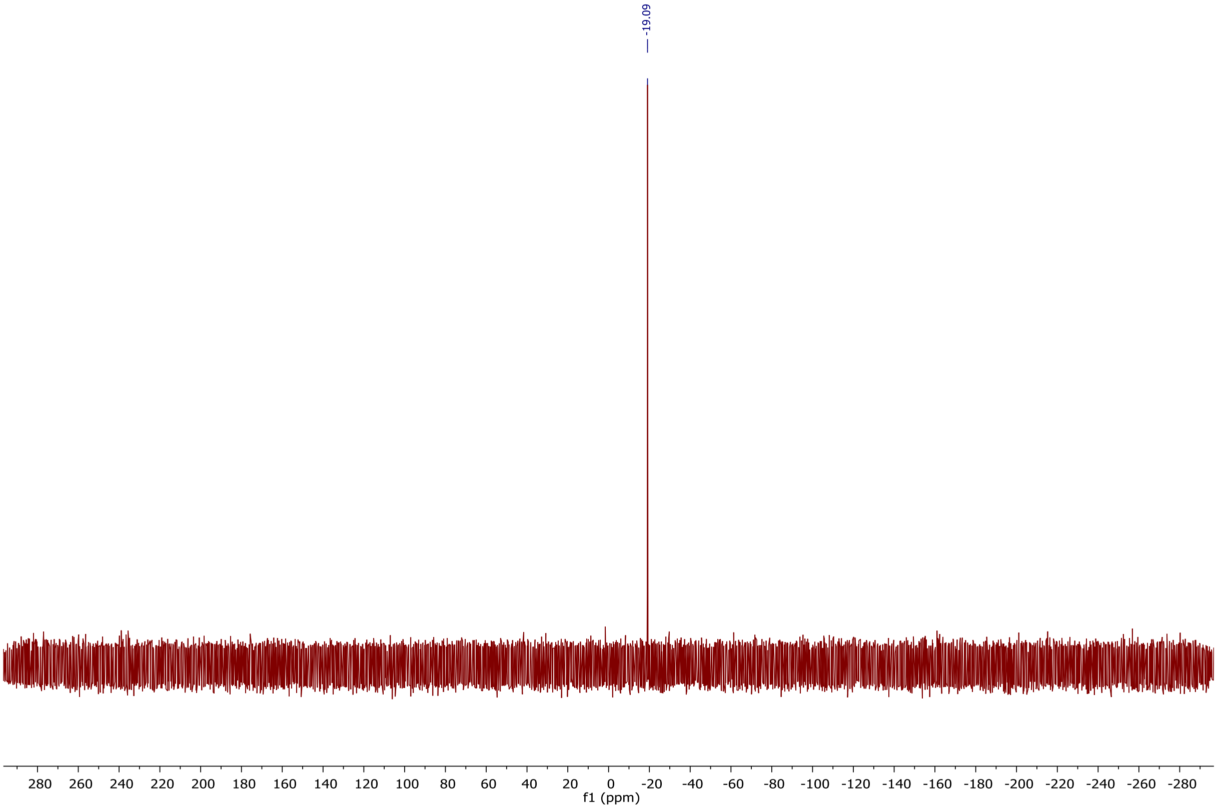

Figure S8. *^{31}P NMR Spectrum (C_6D_6, 298 K, 161.98 MHz) for* ***[(BDI)Ca{NCy}$_2$C-PPh$_2$)] (5).***

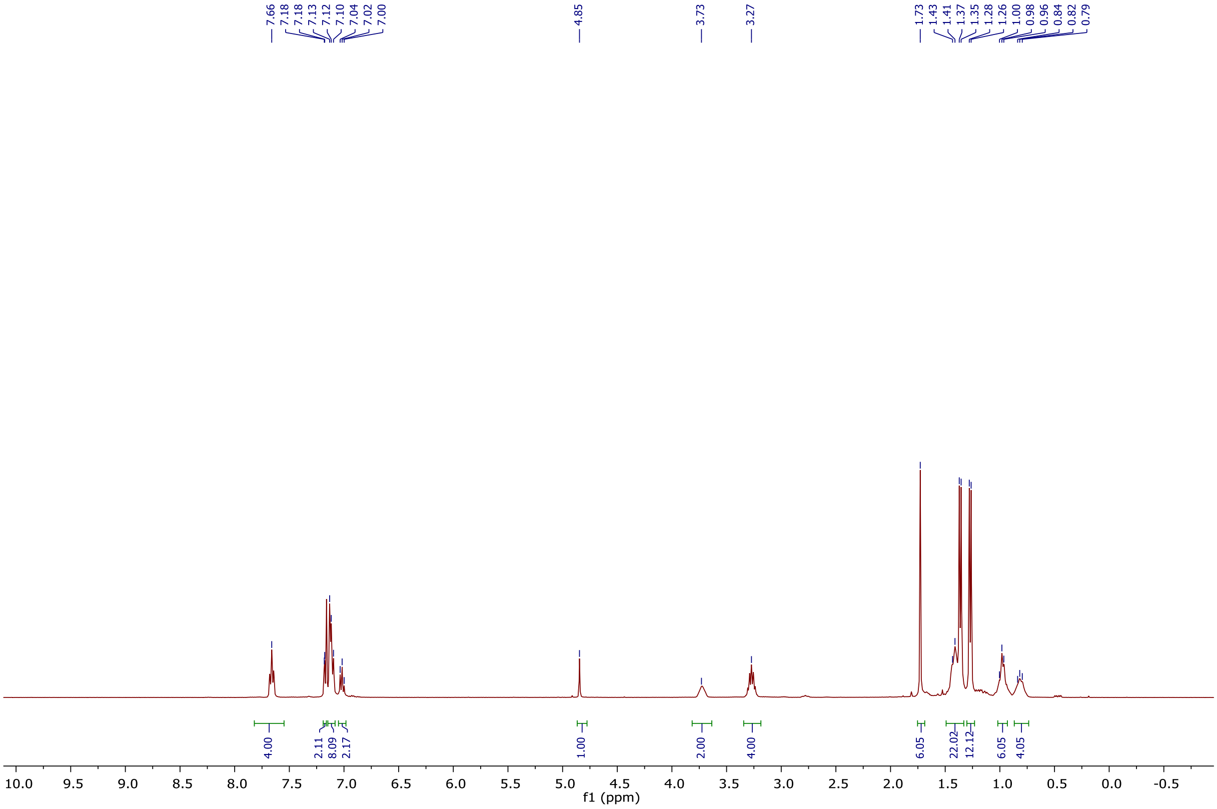

Figure S9. *^{1}H NMR Spectrum (C_6D_6, 298 K, 400.13 MHz) for* ***[(BDI)Ca{NCy}$_2$C-PPh$_2$)] (5).***

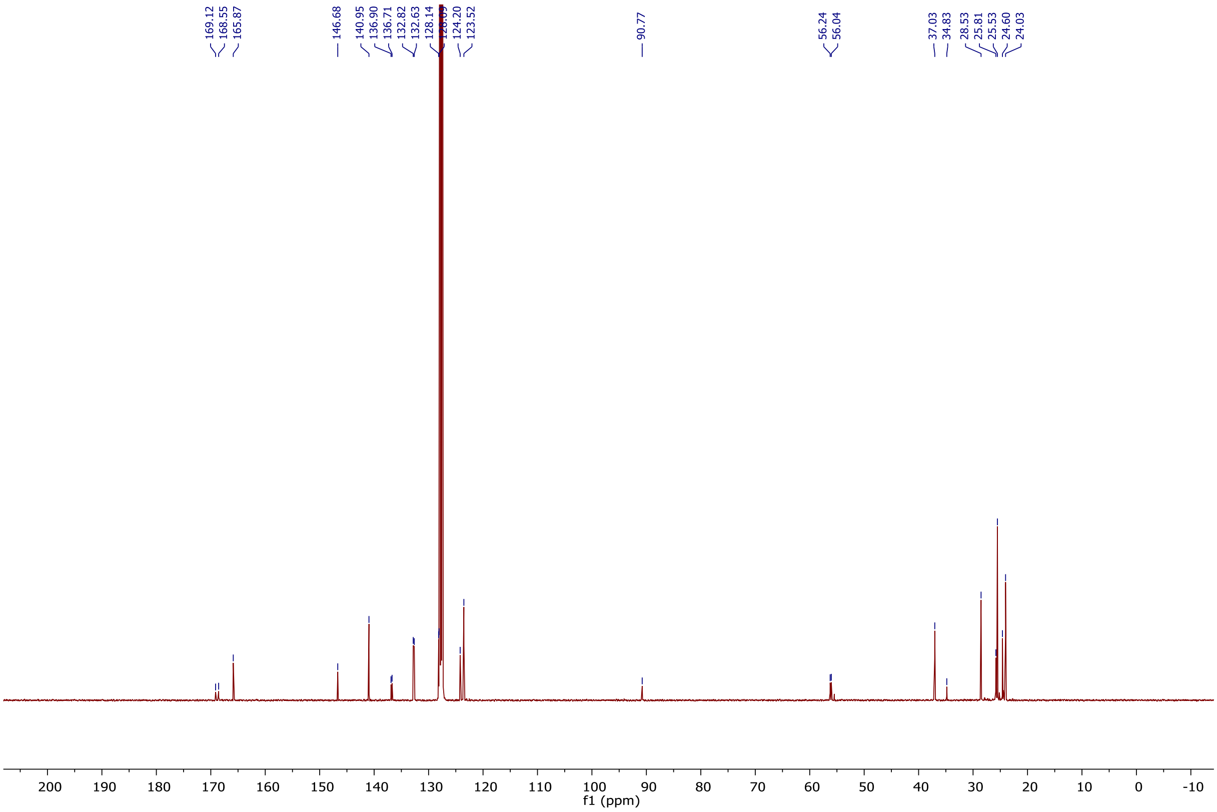

Figure S10. *^{13}C{^{1}H} NMR Spectrum (C_6D_6, 298 K, 100.62 MHz) for* ***[(BDI)Ca{NCy}$_2$C-PPh$_2$)] (5).***

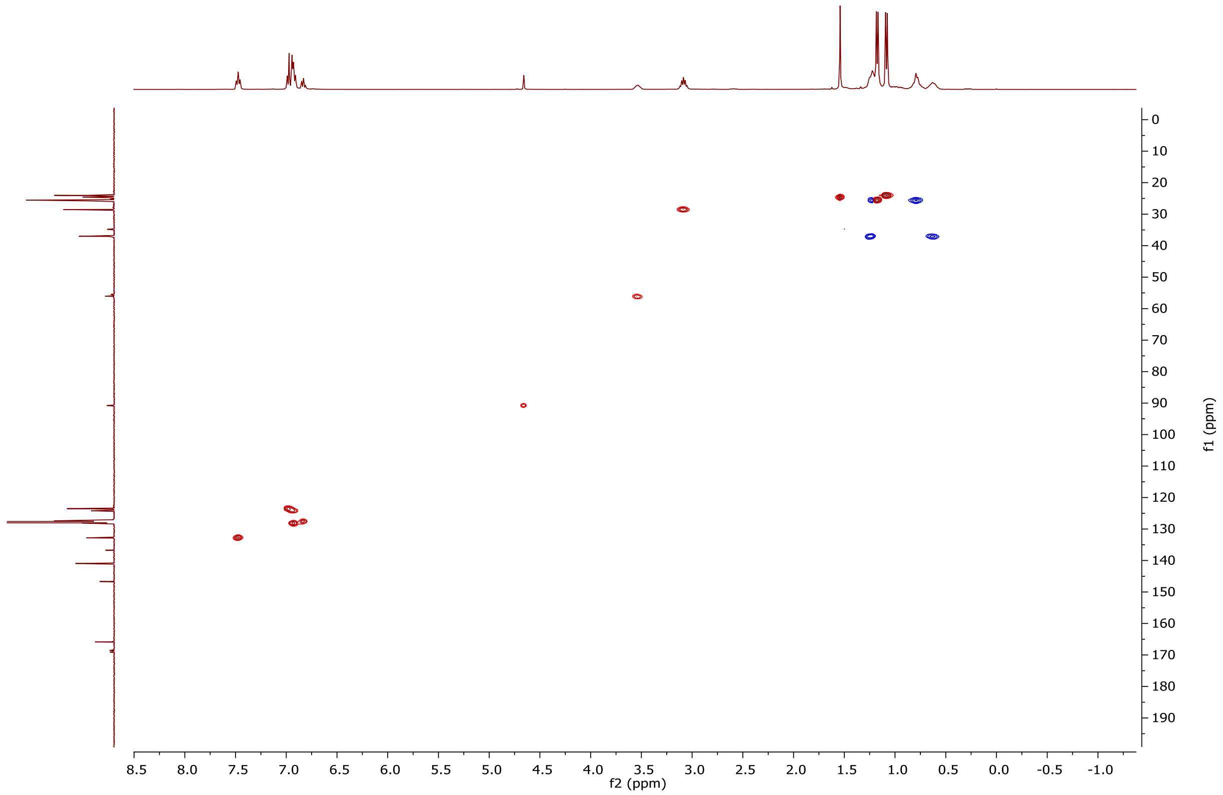

Figure S11. *^{1}H-^{13}C HSQC trace (C_6D_6, 298 K, 400.13, 100.62 MHz) for* ***[(BDI)Ca{NCy}$_2$C-PPh$_2$)] (5).***

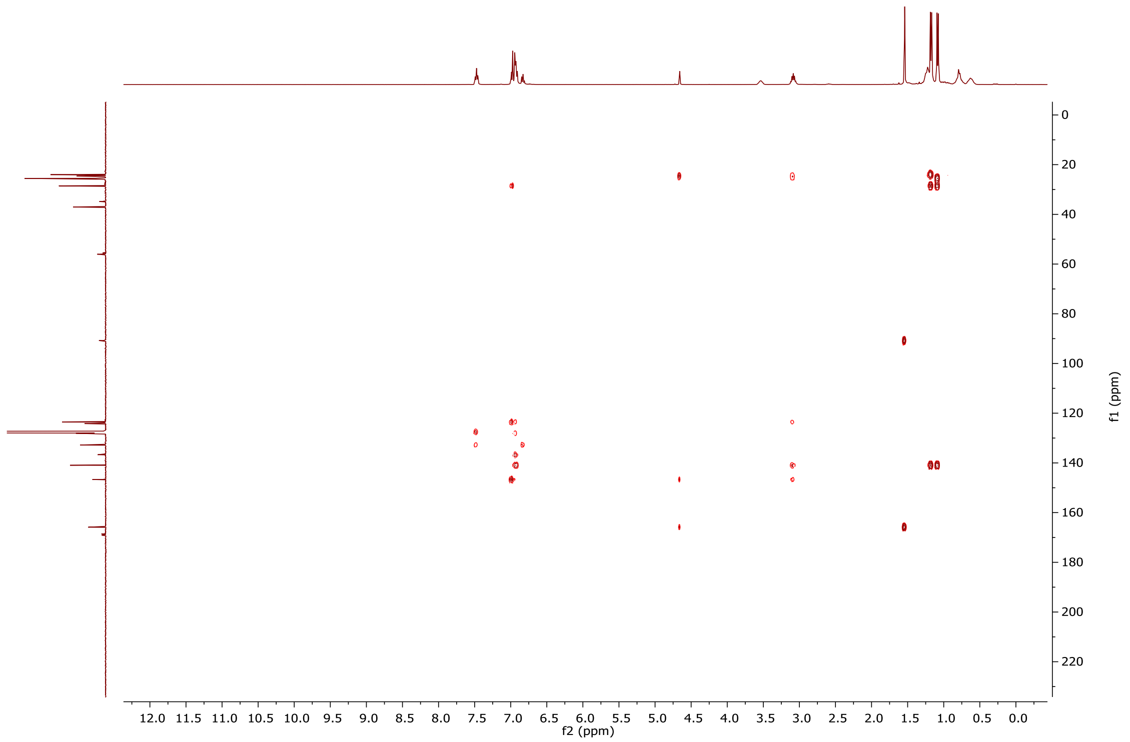

Figure S12. *^{1}H-^{13}C HMBC trace (C_6D_6, 298 K, 400.13, 100.62 MHz) for* ***[(BDI)Ca{NCy}$_2$C-PPh$_2$)] (5).***

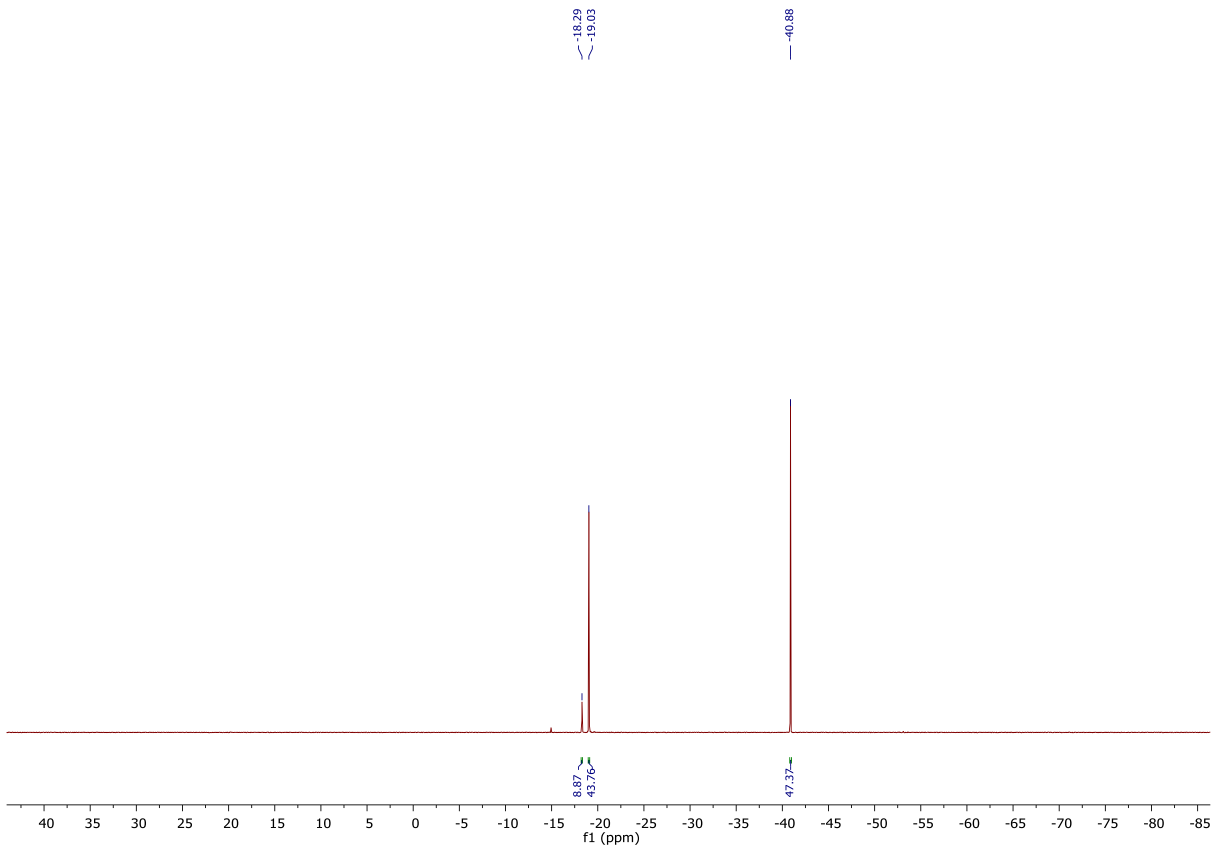

Figure S13: *$^{31}P\{^{1}H\}$ NMR Spectrum (C_6D_6, 298 K, 161.98 MHz, D1 = 30 sec) for the reaction between **5** and Ph_2PH, after heating at 40 °C for 16 hours.*

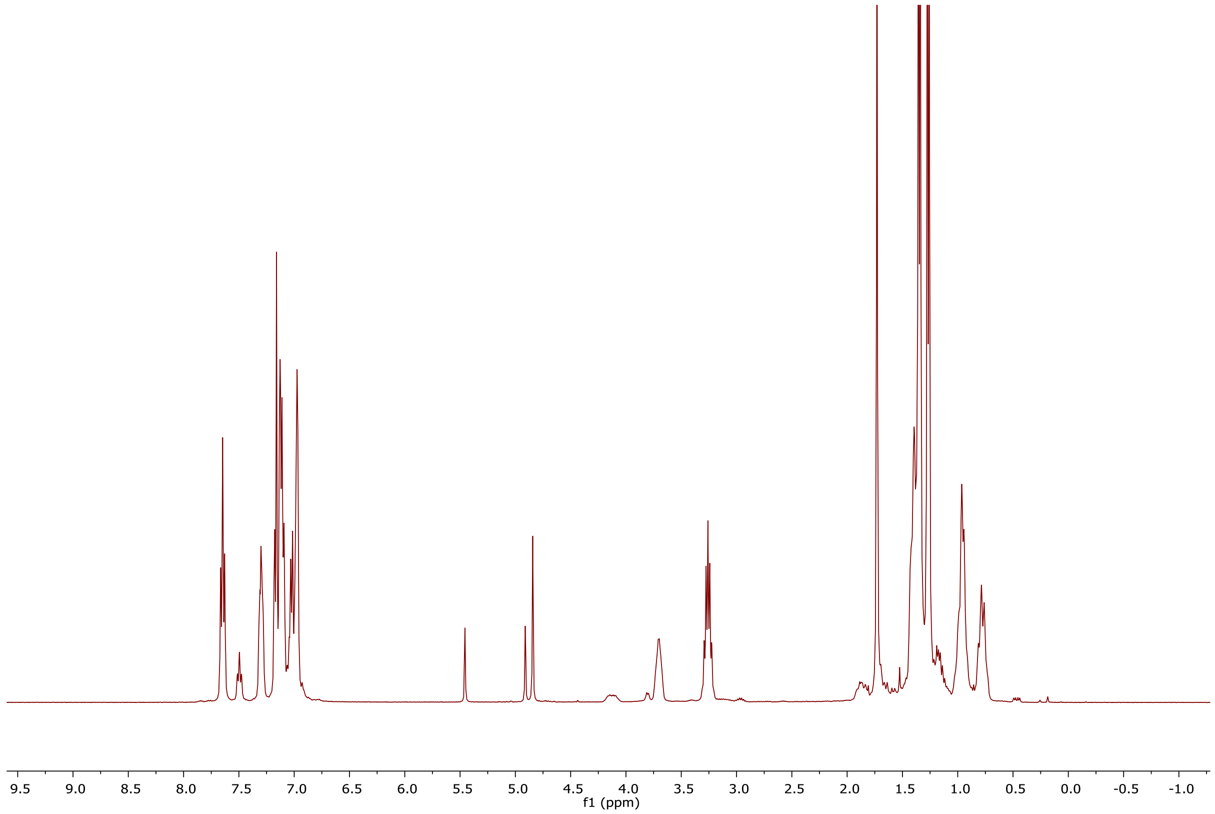

Figure S14. *^{1}H NMR Spectrum (C_6D_6, 298 K, 400.13 MHz) for the reaction between **5** and Ph_2PH, after heating at 40 °C for 16 hours.*

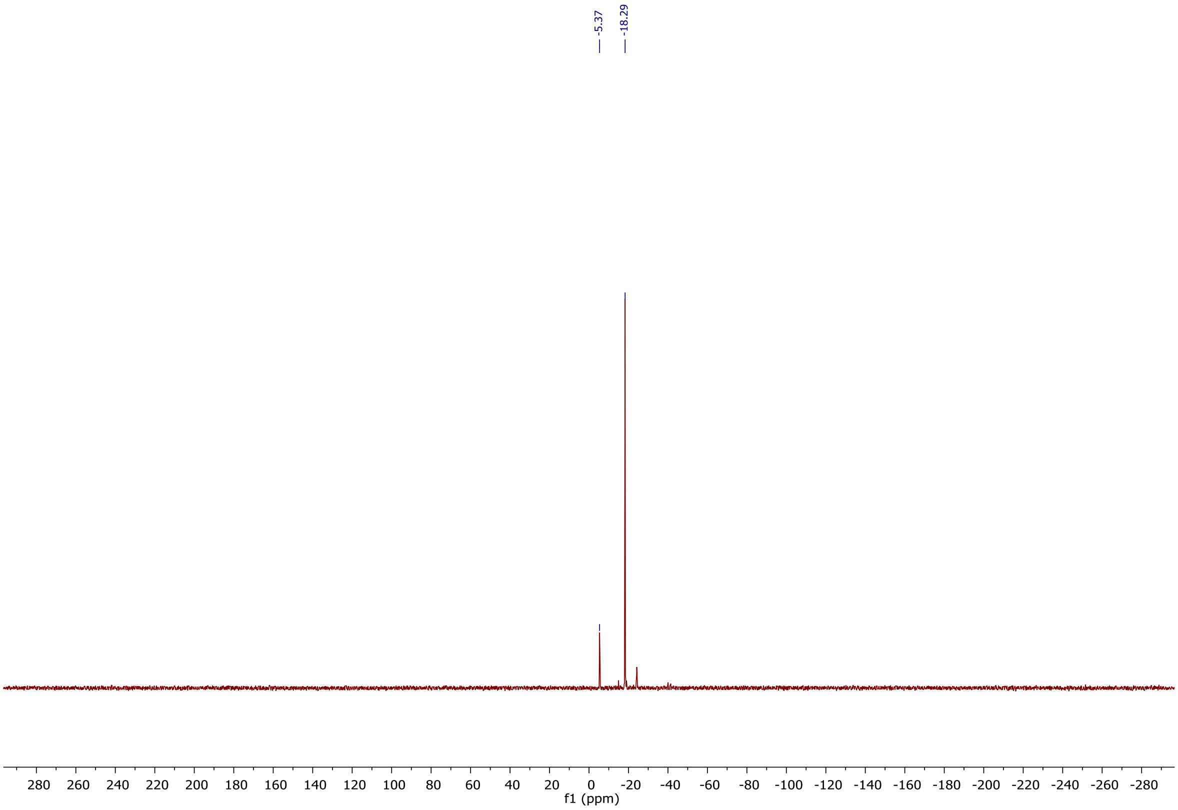

Figure S15. *^{31}P NMR Spectrum (C_6D_6, 298 K, 161.98 MHz) for hydrophosphination of dicyclohexylcarbodiimide with internal standard.*

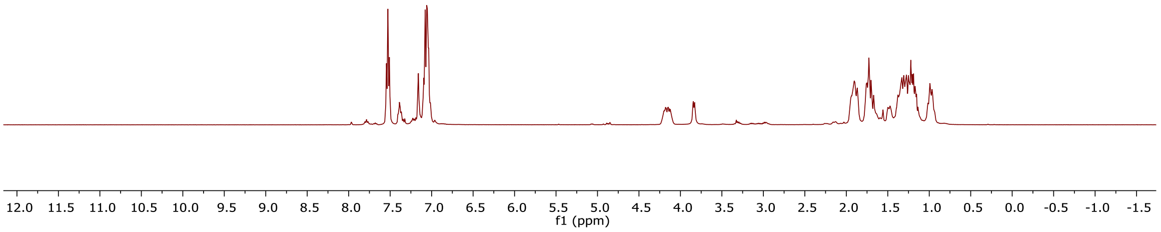

Figure S16. *^{1}H NMR Spectrum (C_6D_6, 298 K, 400.13 MHz) for hydrophosphination of dicyclohexylcarbodiimide with internal standard.*

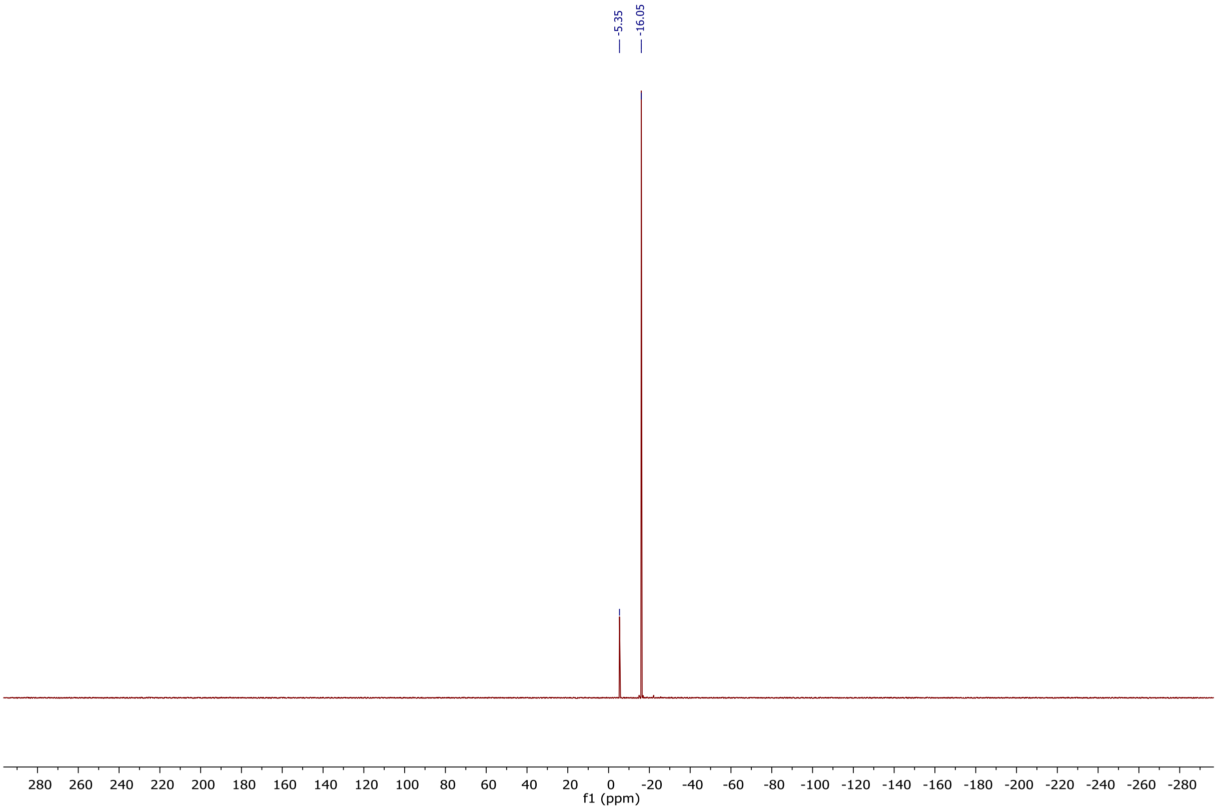

Figure S17. *^{31}P NMR Spectrum (C_6D_6, 298 K, 161.98 MHz) for hydrophosphination of styrene with internal standard.*

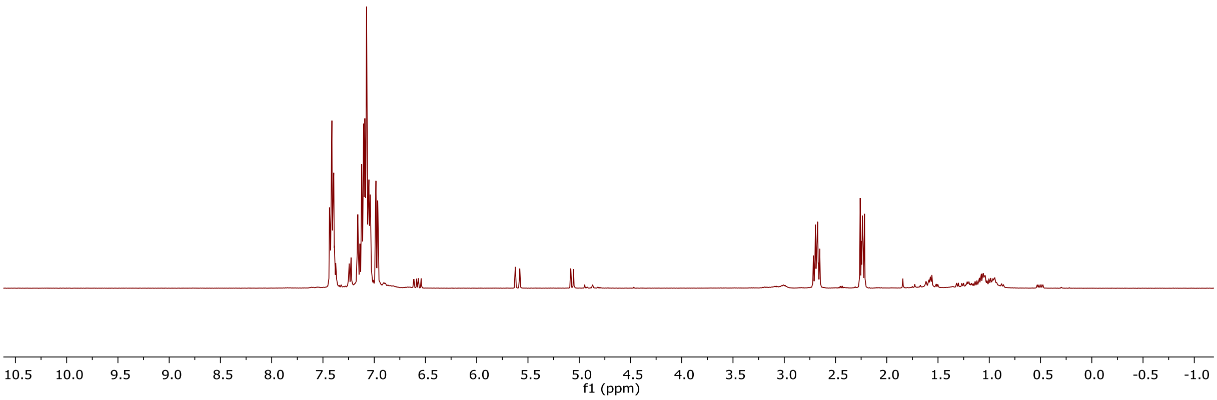

Figure S18*. ^{1}H NMR Spectrum (C_6D_6, 298 K, 400.13 MHz) for hydrophosphination styrene with internal standard.*

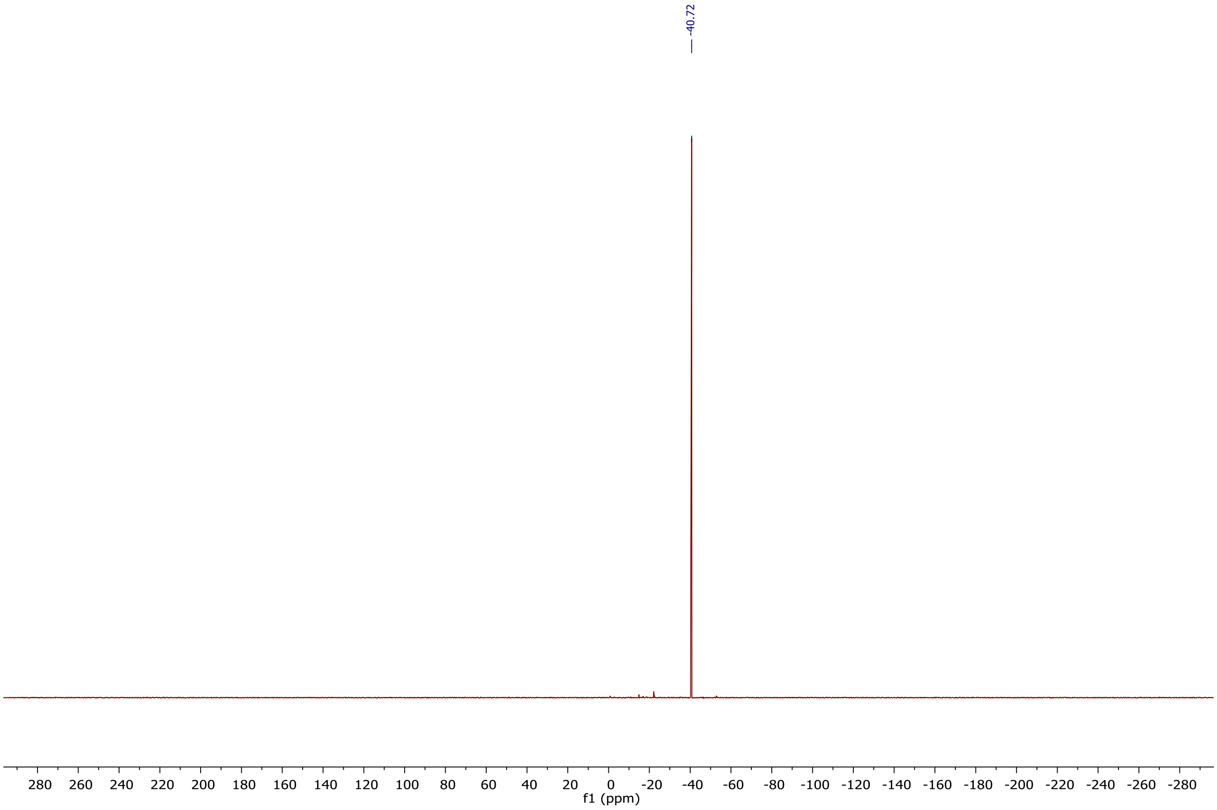

Figure S19. *^{31}P NMR Spectrum (C_6D_6, 298 K, 161.98 MHz) for attempted hydrophosphination of trans-stilbene.*

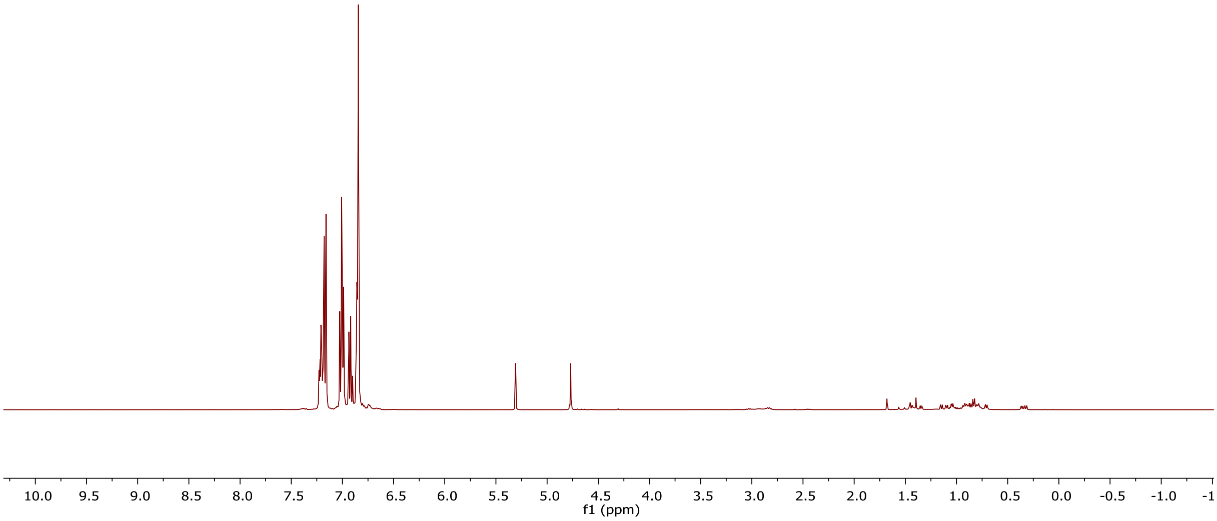

Figure S20. *^{1}H NMR Spectrum (C_6D_6, 298 K, 400.13 MHz) for attempted hydrophosphination of trans-stilbene.*

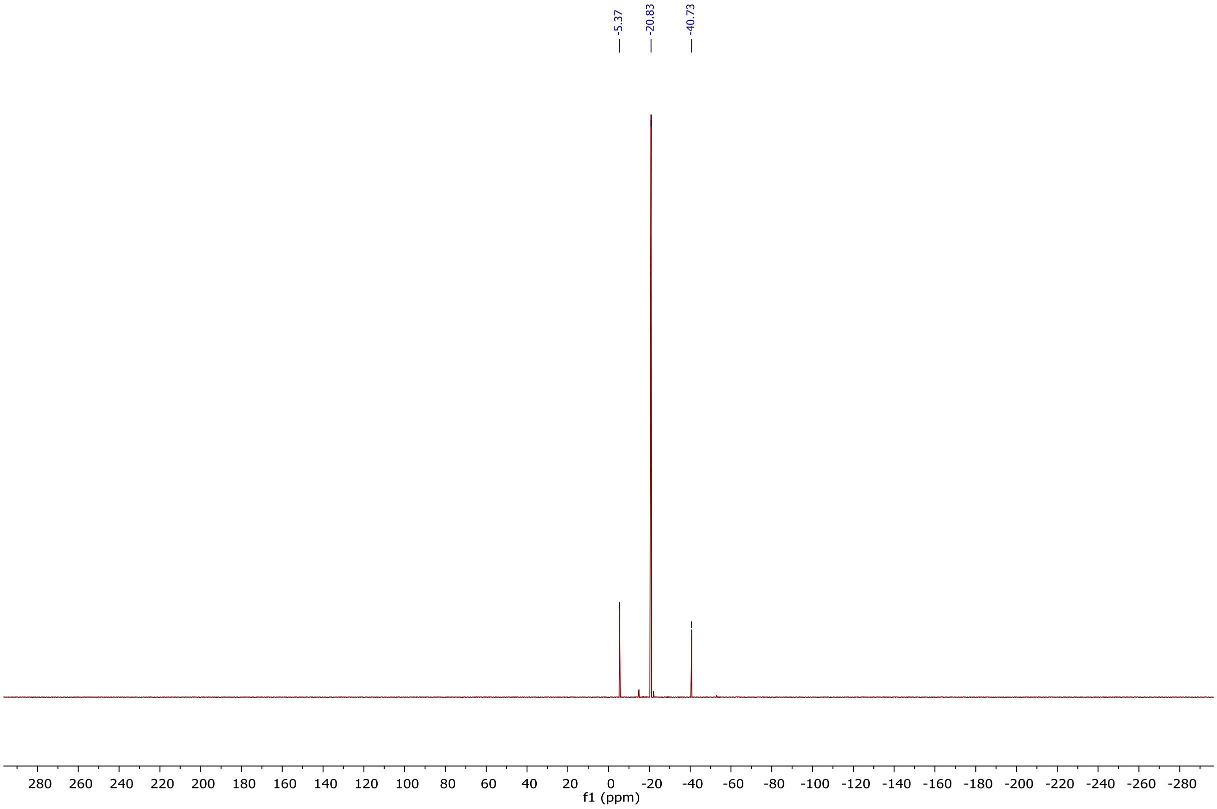

Figure S21. *^{31}P NMR Spectrum (C_6D_6, 298 K, 161.98 MHz) for hydrophosphination of diphenylethene with internal standard.*

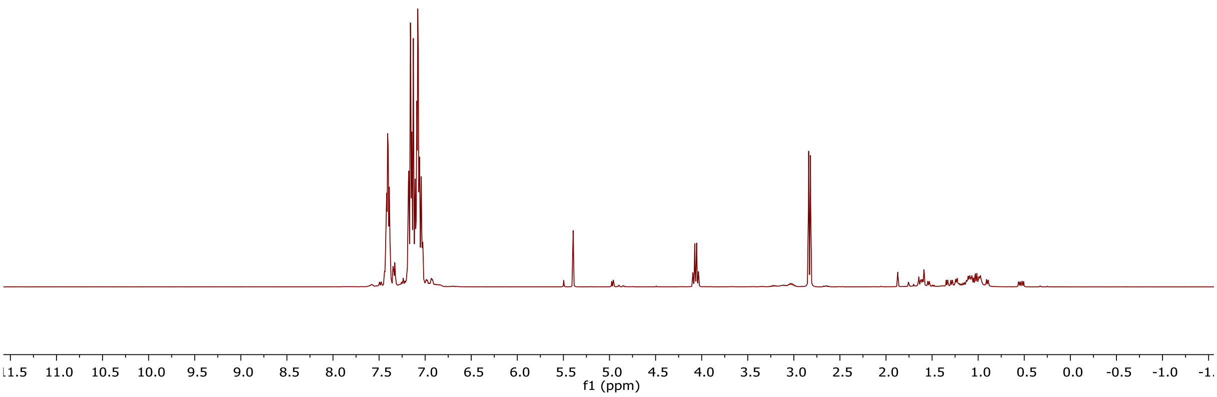

Figure S22. *^{1}H NMR Spectrum (C_6D_6, 298 K, 400.13 MHz) for hydrophosphination of diphenylethene with internal standard.*

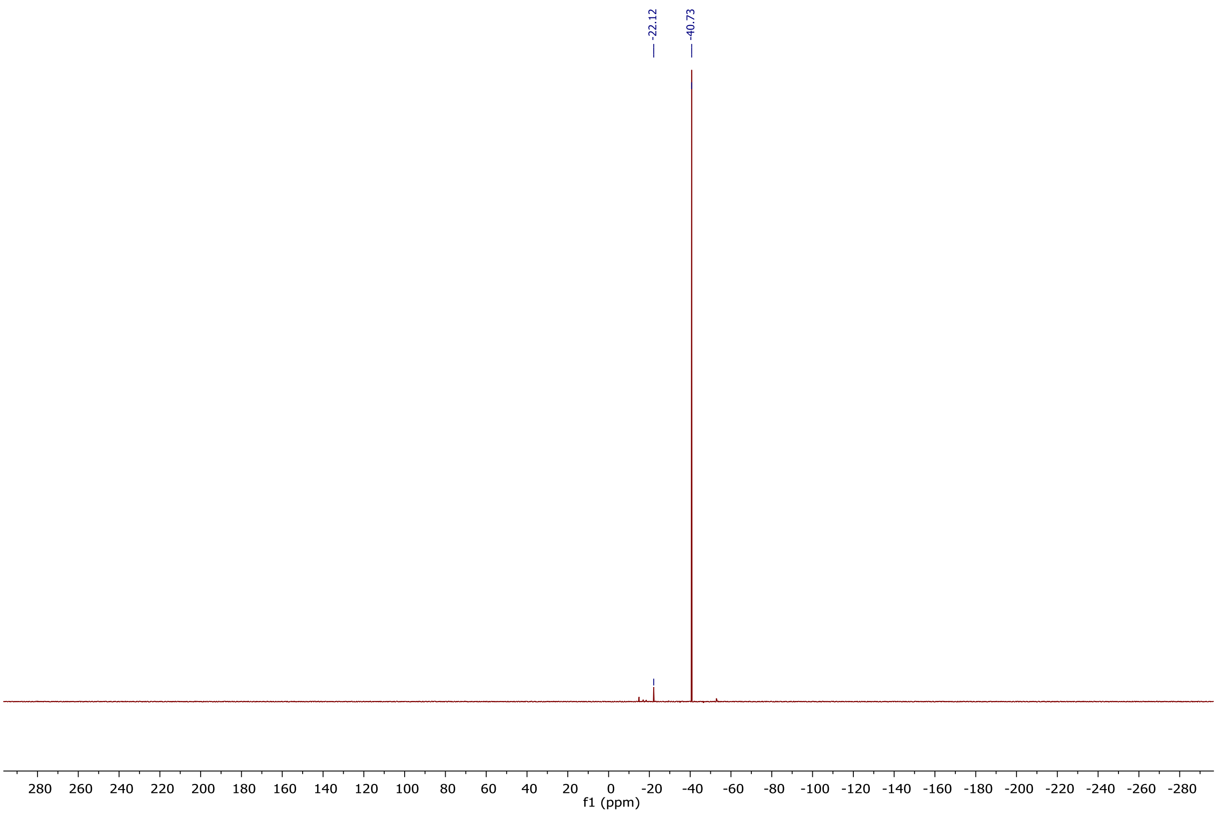

Figure S23. *^{31}P NMR Spectrum (C_6D_6, 298 K, 161.98 MHz) for attempted hydrophosphination of 1-hexene.*

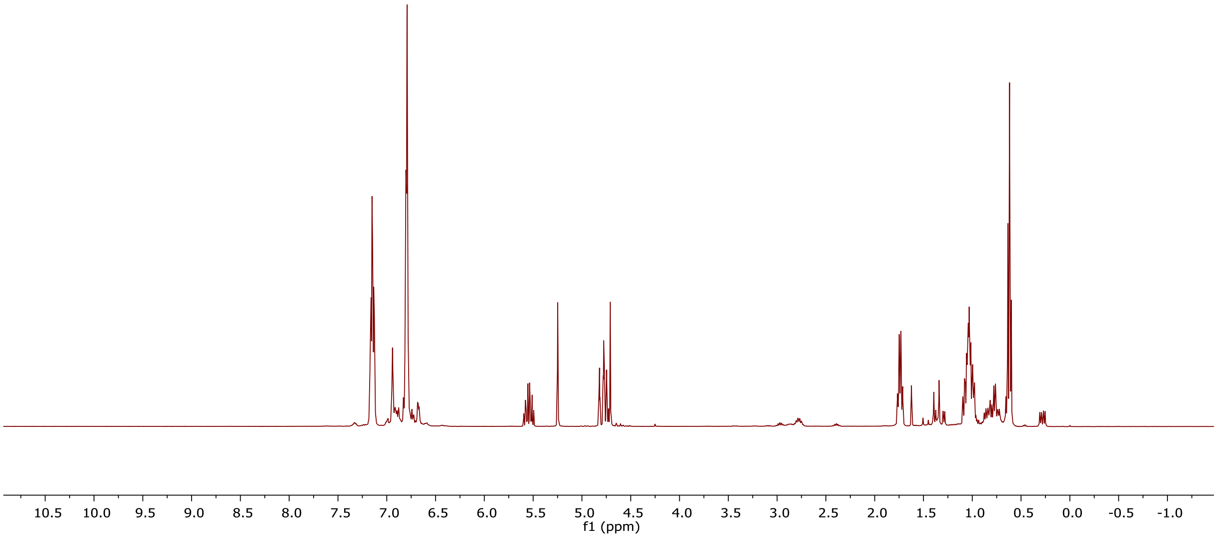

Figure S24. *^{1}H NMR Spectrum (C_6D_6, 298 K, 400.13 MHz) for attempted hydrophosphination of 1-hexene.*

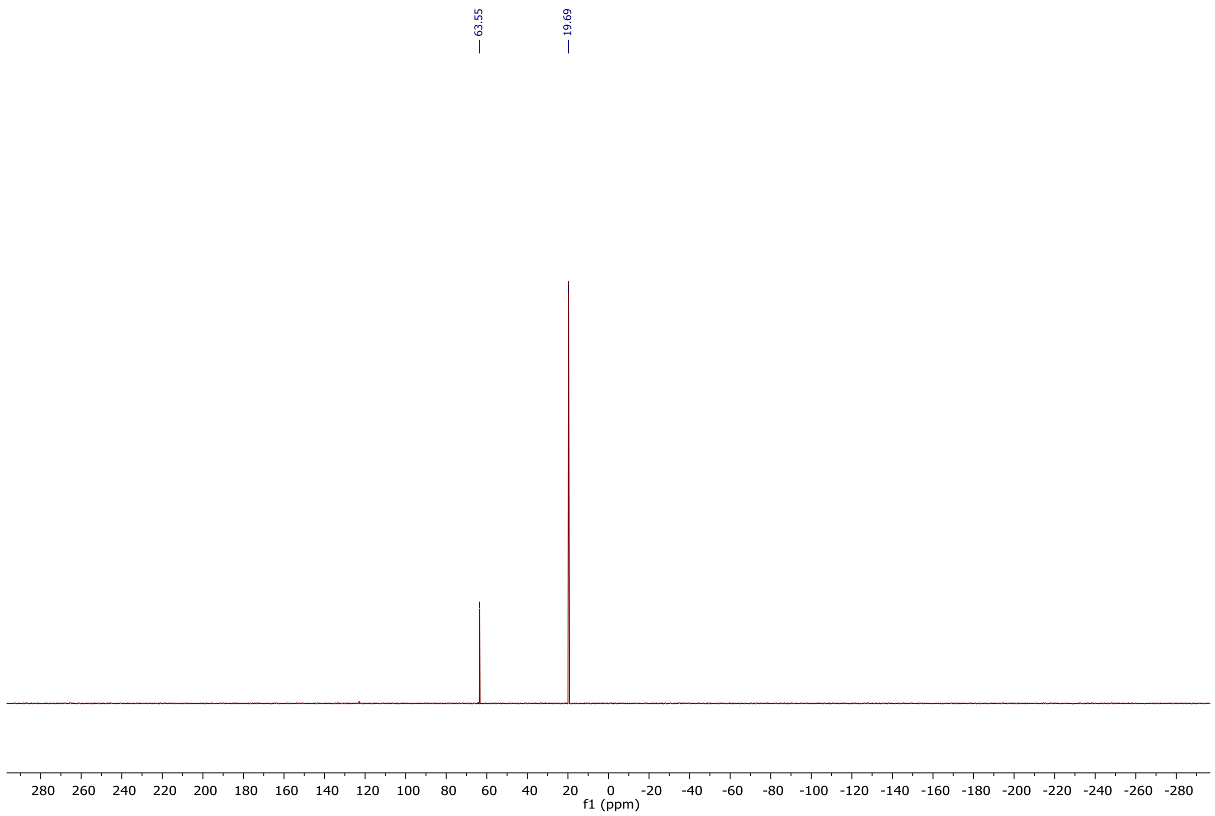

Figure S25. *$^{31}P\{^{1}H\}$ NMR Spectrum (C_6D_6, 298 K, 161.98 MHz) of an equimolar reaction of* ***3*** *and t-Bu_2PH, evidencing the conversion to ca. 25% compound* ***6*** *after 2 weeks.*

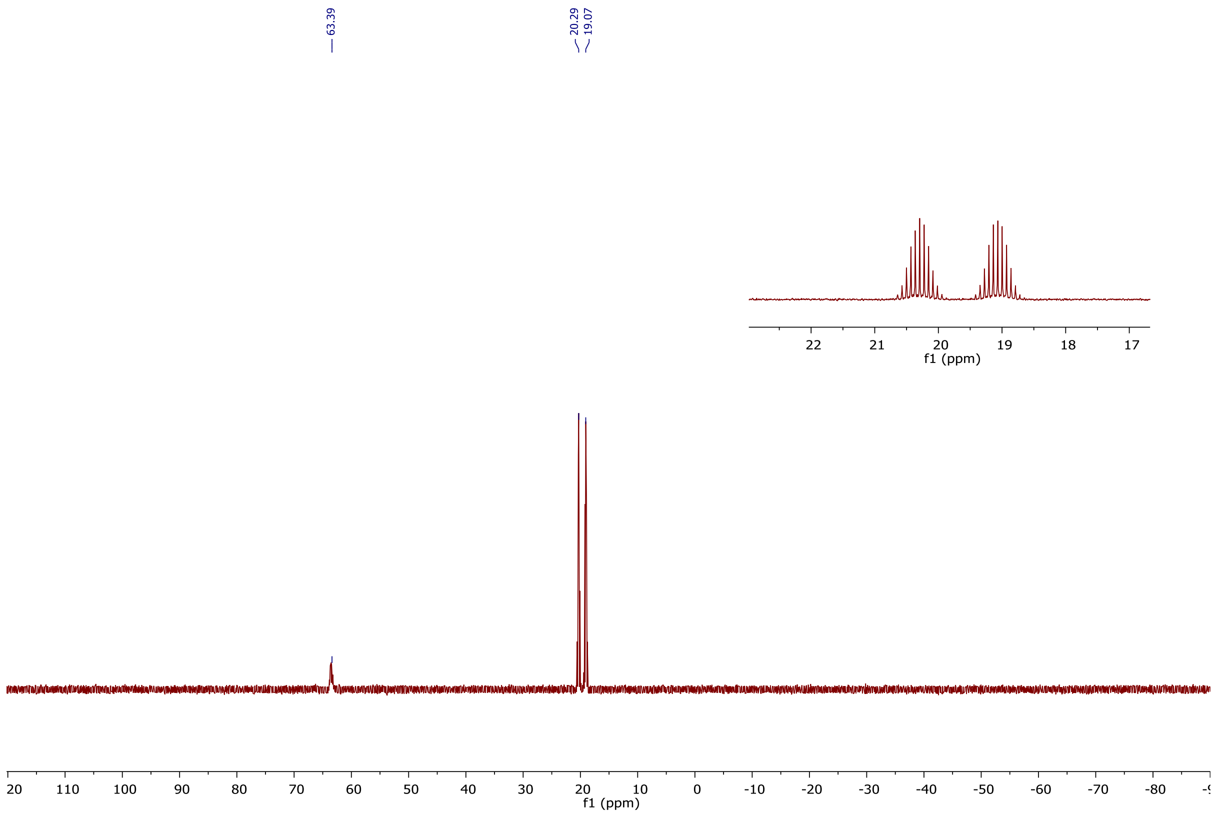

Figure S26. *^{31}P NMR Spectrum (C_6D_6, 298 K, 161.98 MHz) of an equimolar reaction of* ***3*** *and t-Bu_2PH, evidencing the conversion to ca. 25% compound* ***6*** *after 2 weeks.*

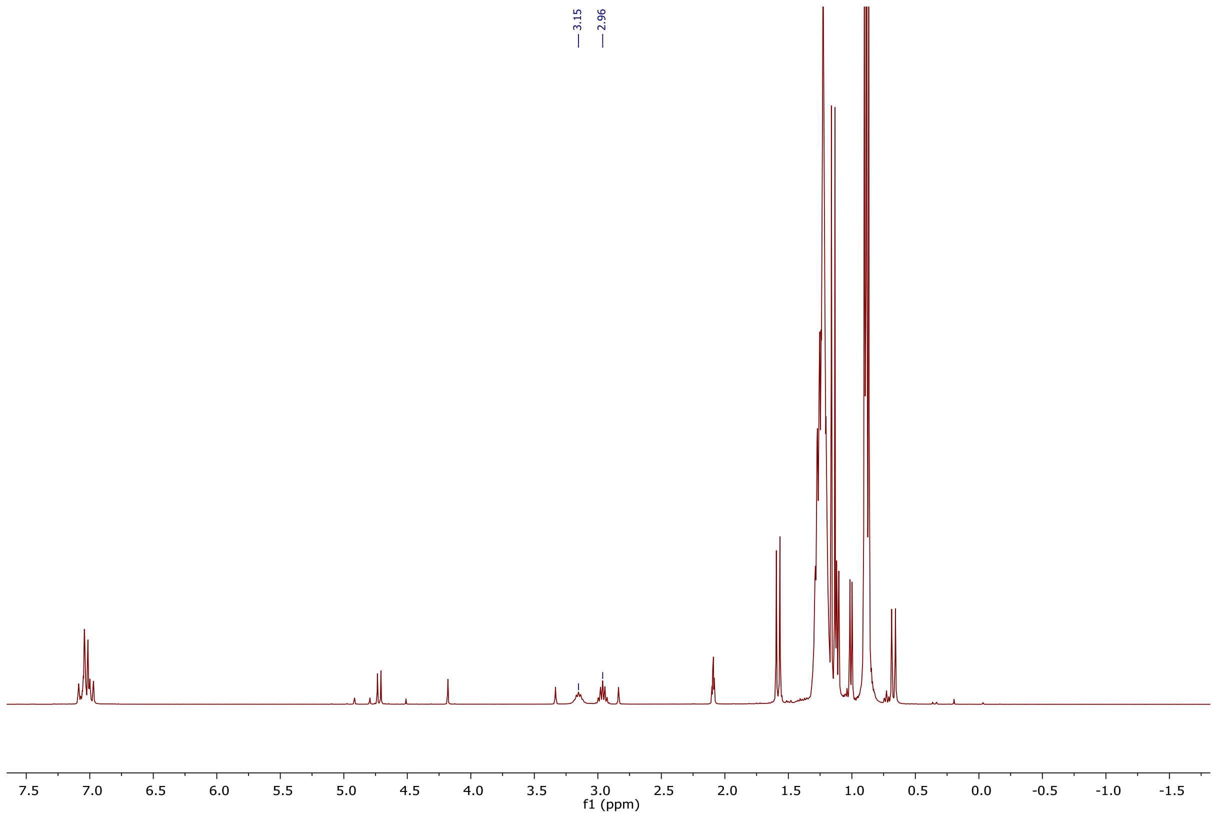

Figure S27. *^{1}H NMR Spectrum (C_6D_6, 298 K, 400.13 MHz) of an equimolar reaction of **3** and t-Bu_2PH, evidencing the conversion to ca. 25% compound **6** after 2 weeks*

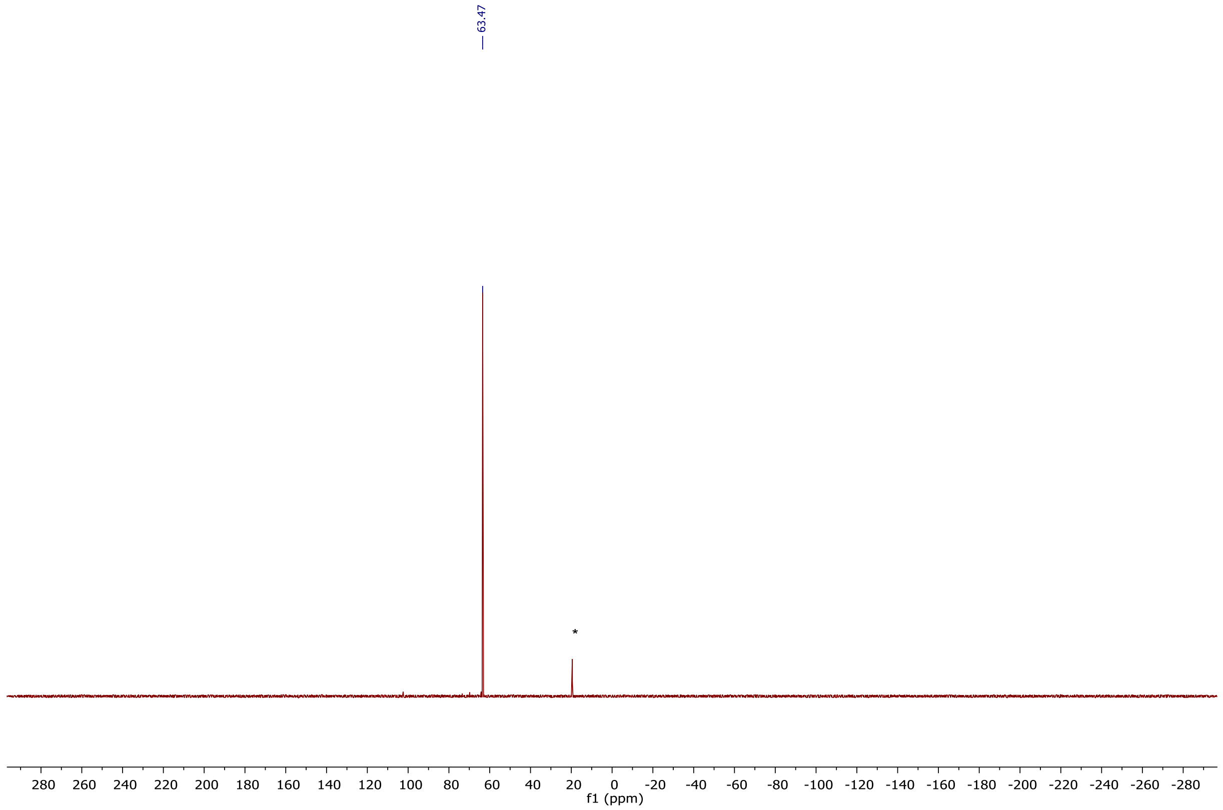

Figure S28. *$^{31}P\{^1H\}$ NMR Spectrum (C_6D_6, 298 K, 161.98 MHz) for **[(BDI)Ca(H)P^{t}Bu$_2$Ca(BDI)] (6).*** = ^{t}Bu$_2$PH*

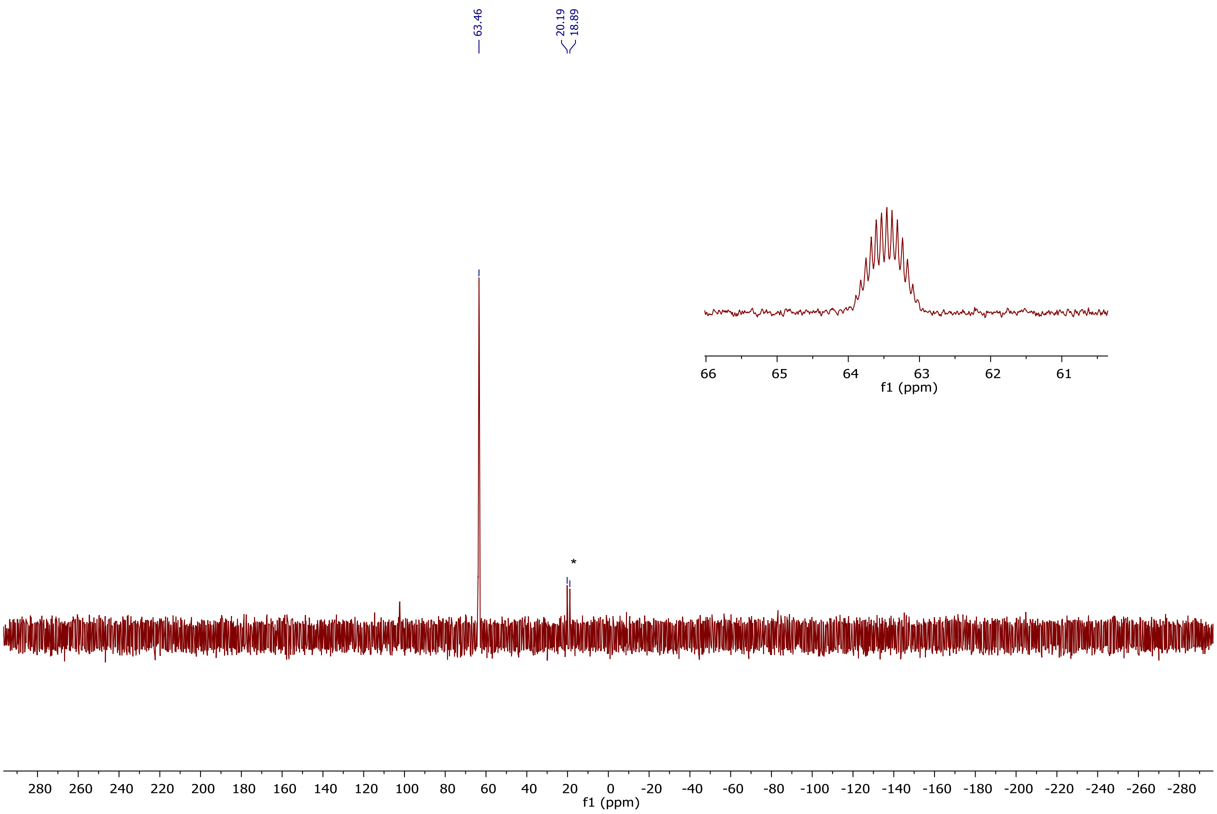

Figure S29. *^{31}P NMR Spectrum (C_6D_6, 298 K, 161.98 MHz) for* ***[(BDI)Ca(H)P^{t}Bu$_2$Ca(BDI)] (6).*** ** = ^{t}Bu$_2$PH*

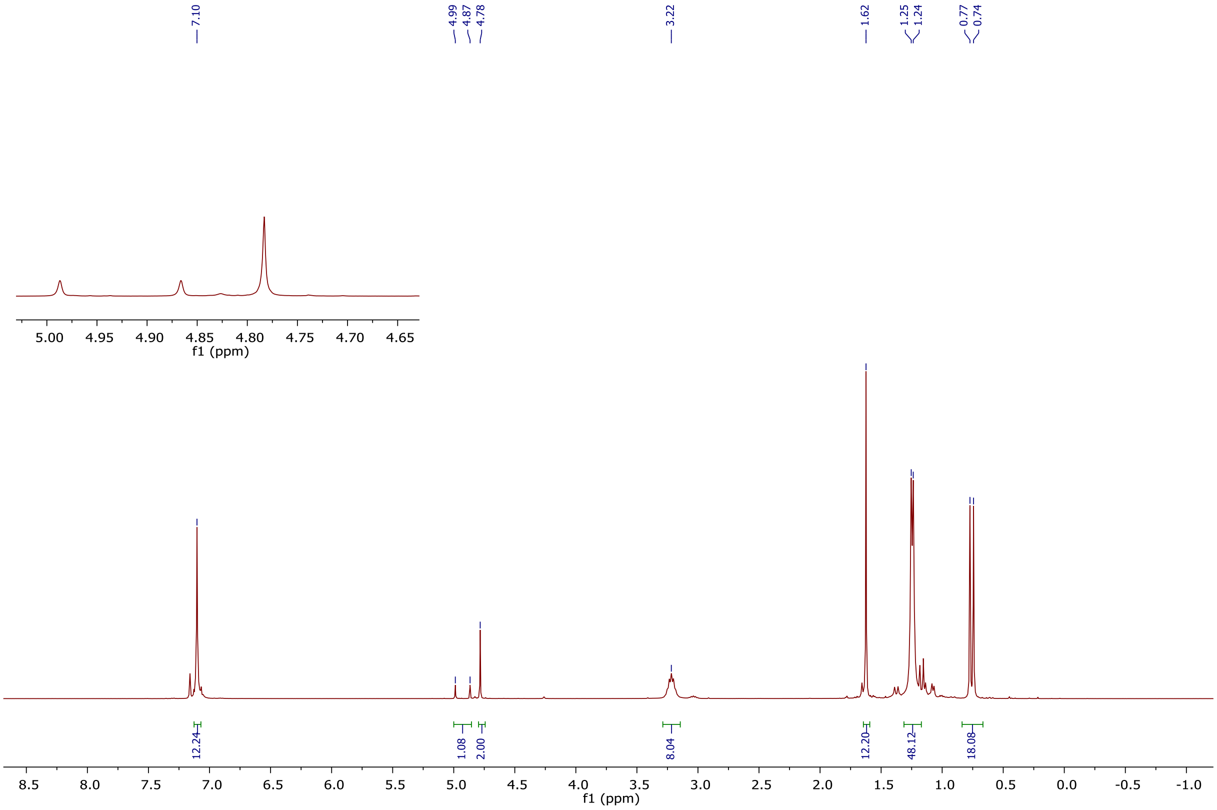

Figure S30**. ^{1}H NMR Spectrum (C_6D_6, 298 K, 400.13 MHz) for* ***[(BDI)Ca(H)P^{t}Bu$_2$Ca(BDI)] (6).

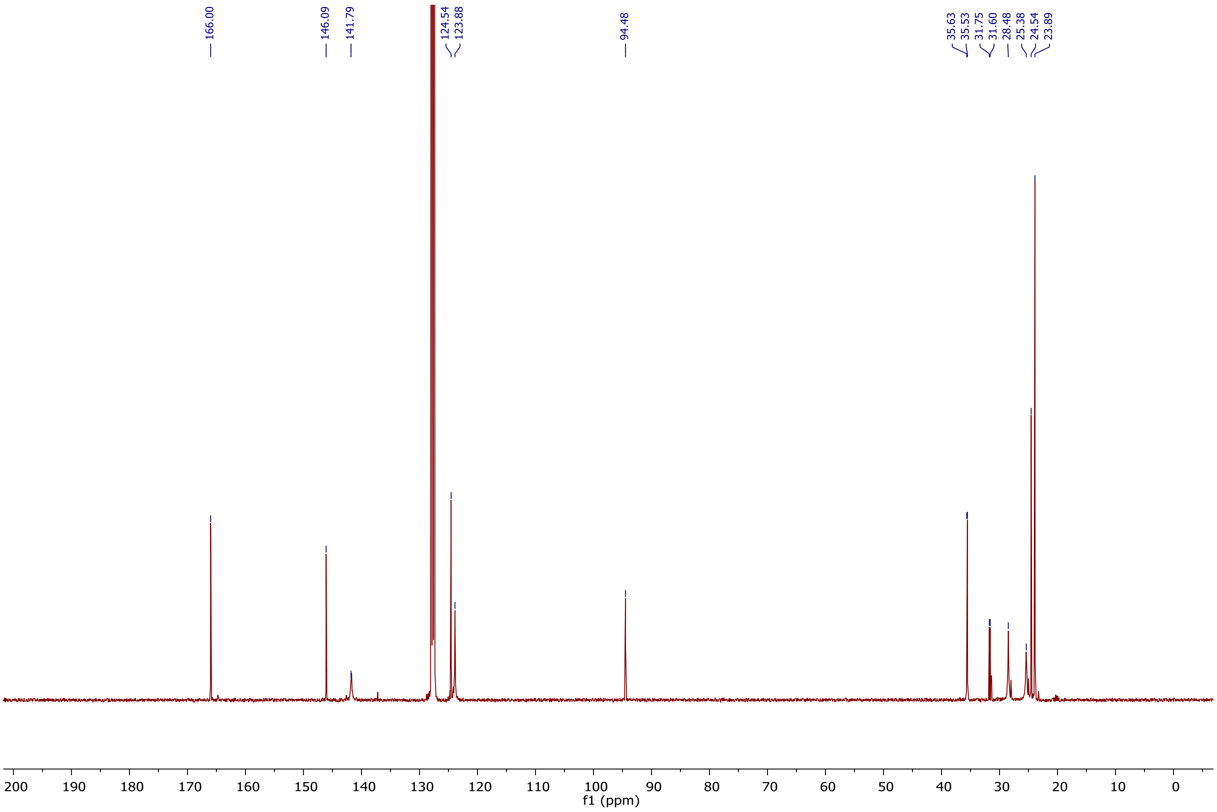

Figure S31. *$^{13}C\{^{1}H\}$ NMR Spectrum (C_6D_6, 298 K, 100.62 MHz) for* ***[(BDI)Ca(H)P^{t}Bu$_2$Ca(BDI)] (6).***

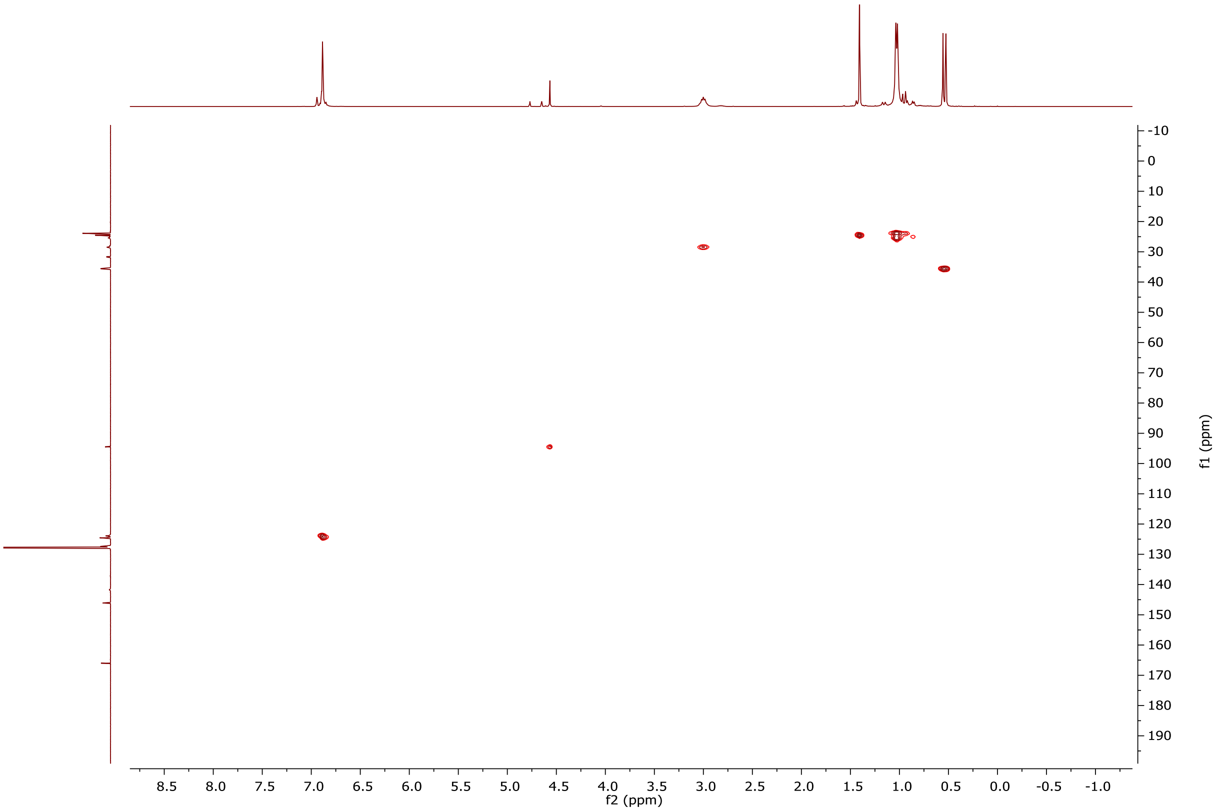

Figure S32. *^{1}H-^{13}C HSQC trace (C_6D_6, 298 K, 400.13, 100.62 MHz) for* ***[(BDI)Ca(H)P^{t}Bu$_2$Ca(BDI)] (6).***

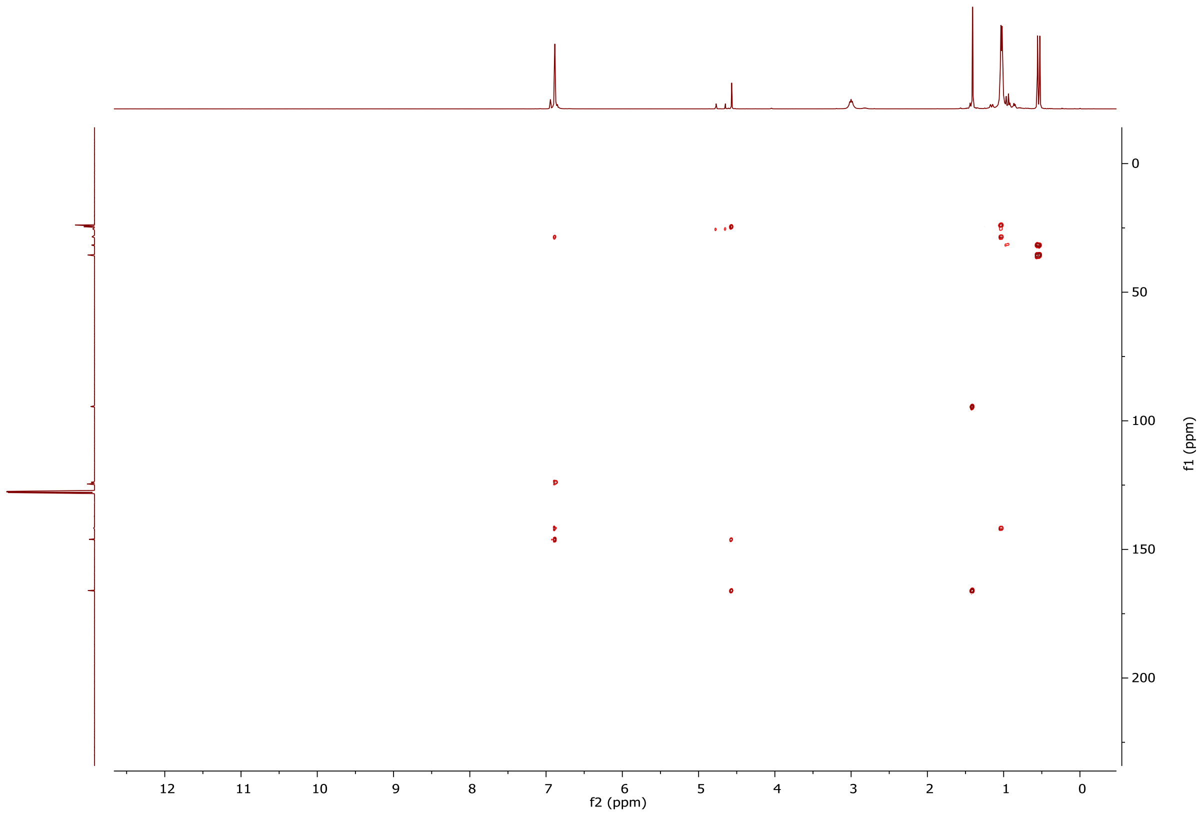

Figure S33. *^{1}H-^{13}C HMBC trace (C_6D_6, 298 K, 400.13, 100.62 MHz) for* ***[(BDI)Ca(H)P^{t}Bu$_2$Ca(BDI)] (6).***

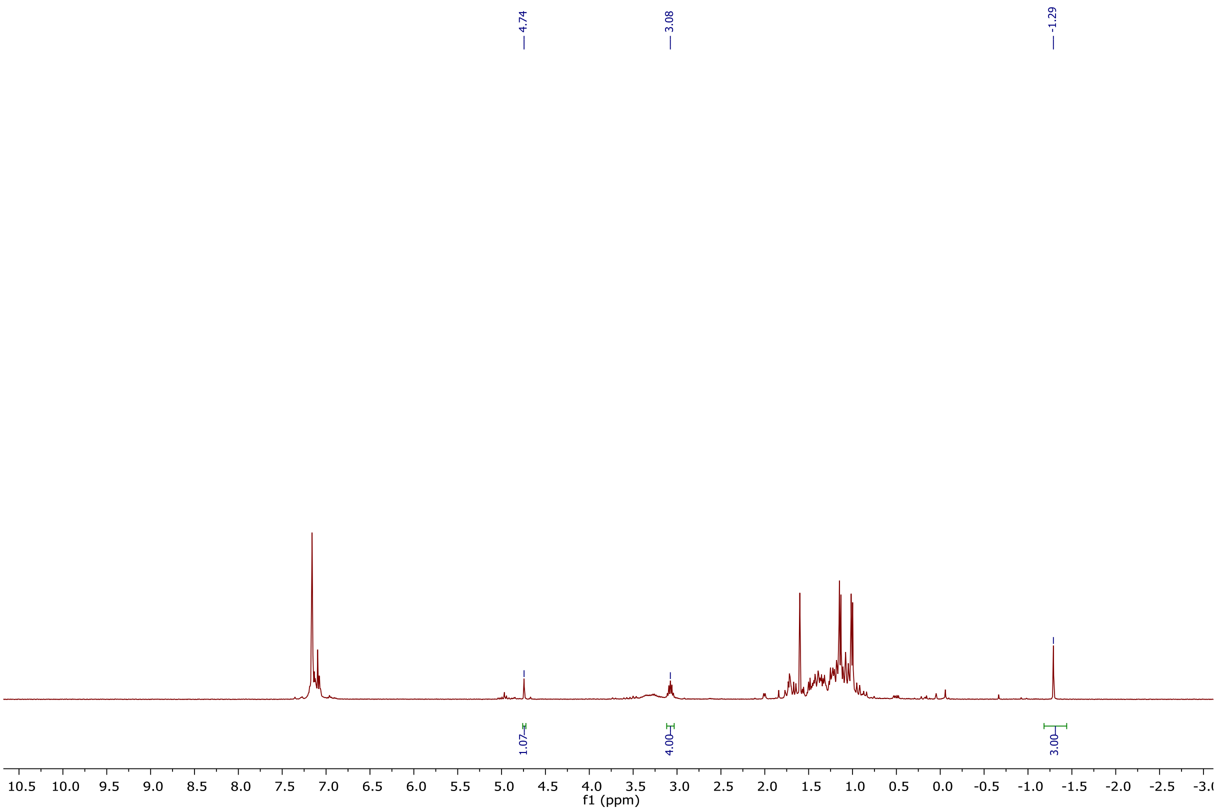

Figure S34. *^{1}H NMR Spectrum (C_6D_6, 298 K, 400.13 MHz) from reacting 0.5 equiv. $ZnMe_2$ with* ***[(BDI)Ca(H)P^{t}Bu$_2$Ca(BDI)] (6)****. Demonstrating [(BDI)CaMe]$_2$ formation.*

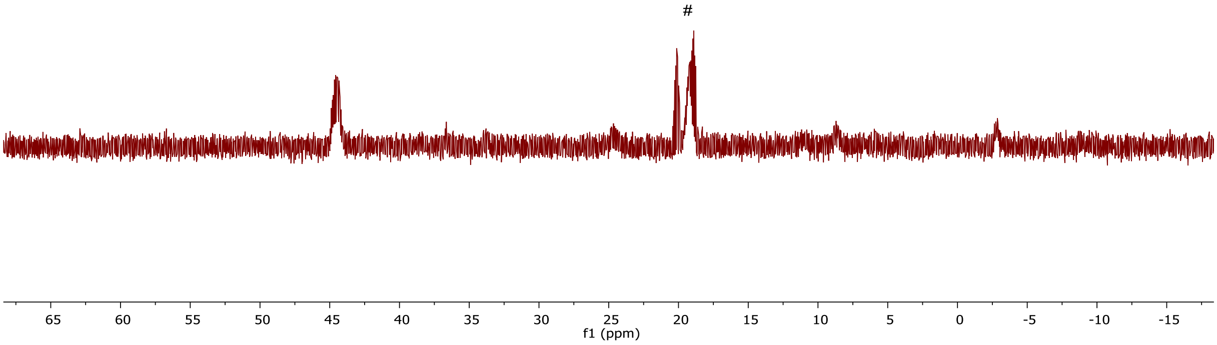

Figure S35. *^{31}P NMR Spectrum (C_6D_6, 298 K, 161.98 MHz) from reacting 0.5 equiv. $ZnMe_2$ with* ***[(BDI)Ca(H)P^{t}Bu$_2$Ca(BDI)] (6).*** *# = ^{t}Bu$_2$PH*

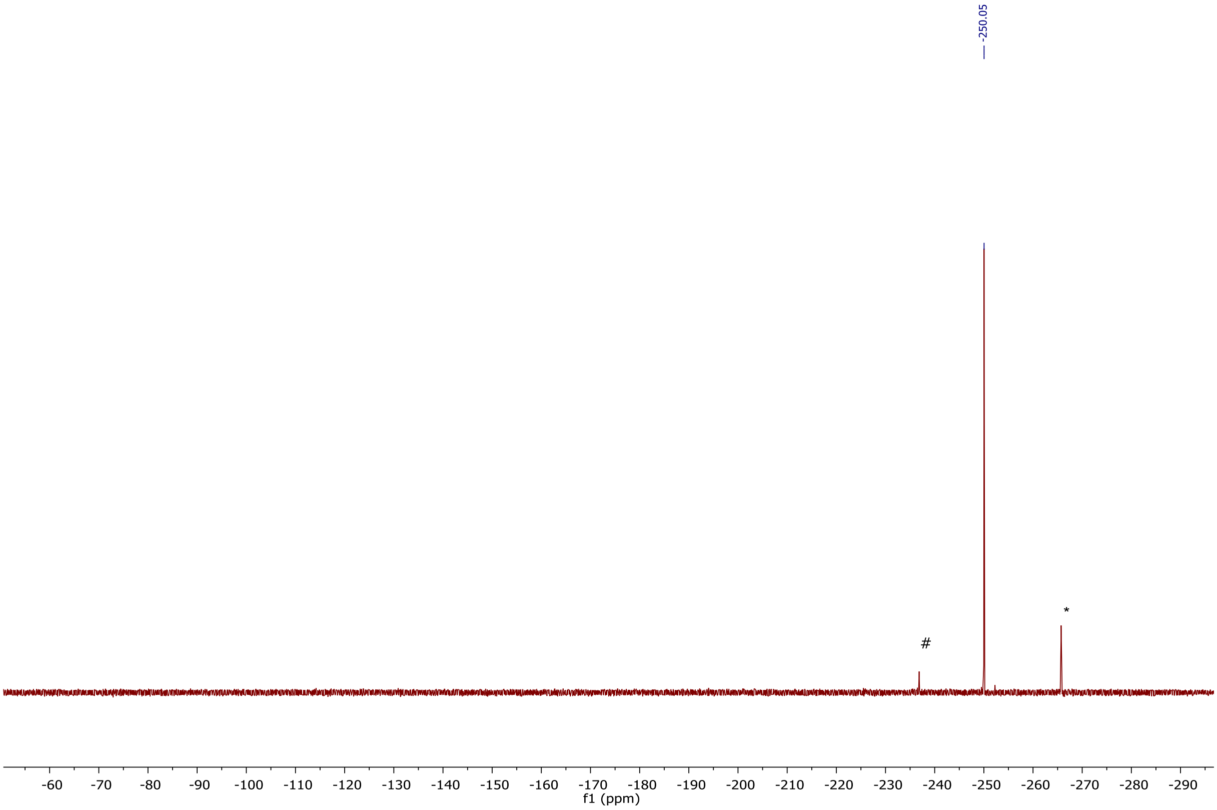

Figure S36. *^{31}P{^{1}H} NMR Spectrum (C_7D_8, 298 K, 161.98 MHz) for* ***[(BDI)Ca(H)P(SiMe$_3$)$_2$Ca(BDI)] (7). # = HP(SiMe$_3$)$_2$.***

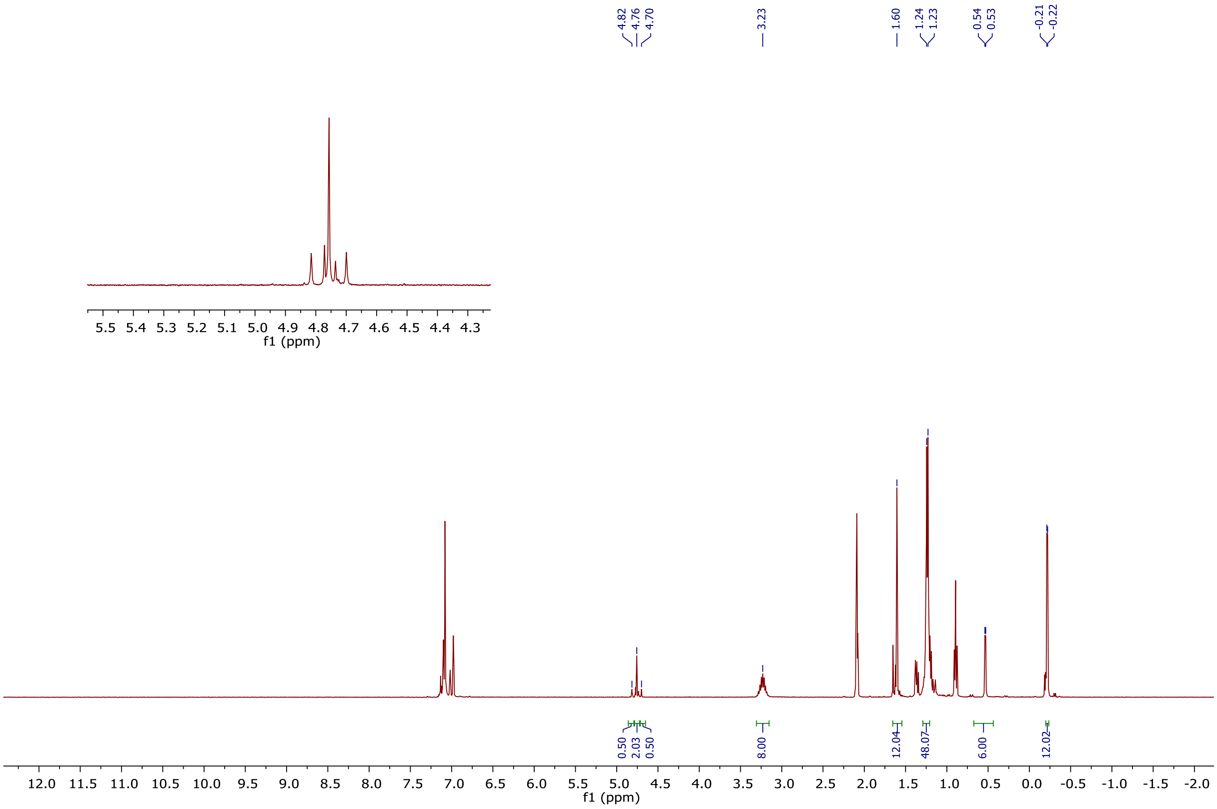

Figure S37. *^{1}H NMR Spectrum (C_7D_8, 298 K, 400.13 MHz) for* ***[(BDI)Ca(H)P(SiMe$_3$)$_2$Ca(BDI)] (7).***

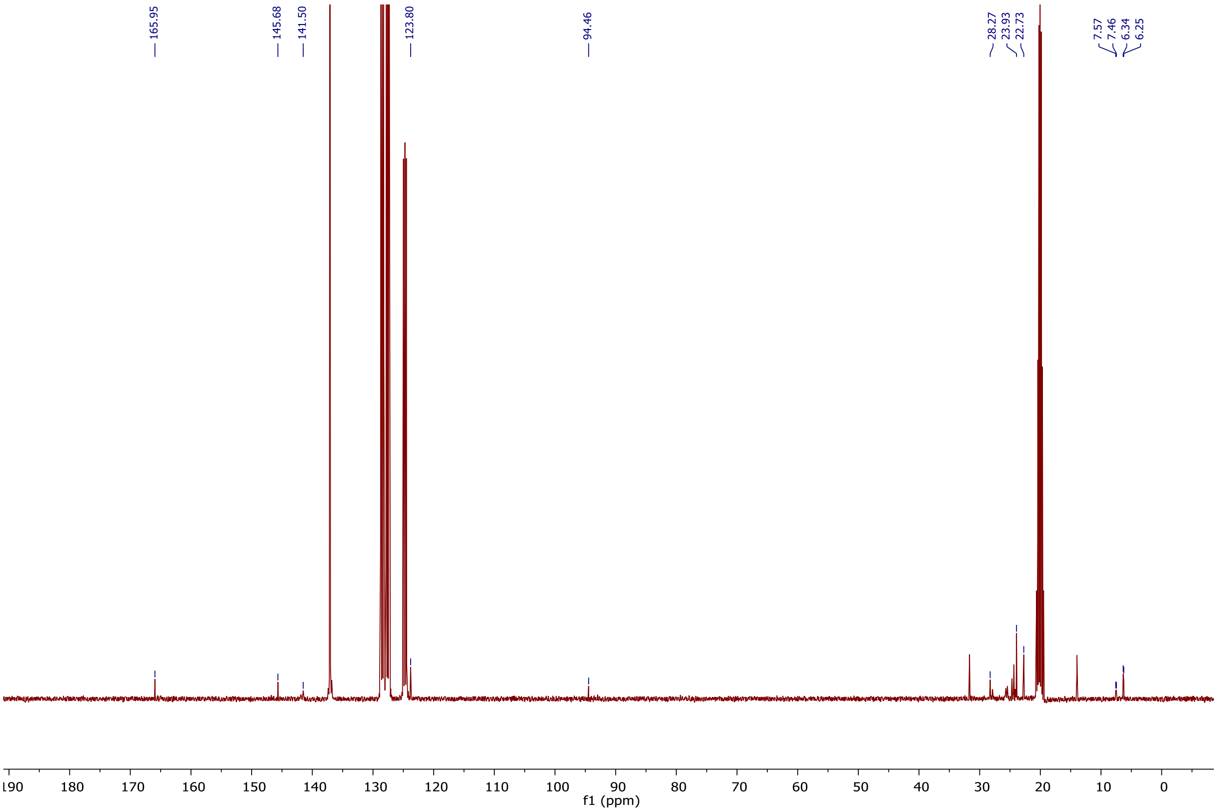

Figure S38. *^{13}C{^{1}H} NMR Spectrum (C_7D_8, 298 K, 100.62 MHz) for* ***[(BDI)Ca(H)P(SiMe$_3$)$_2$Ca(BDI)] (7).***

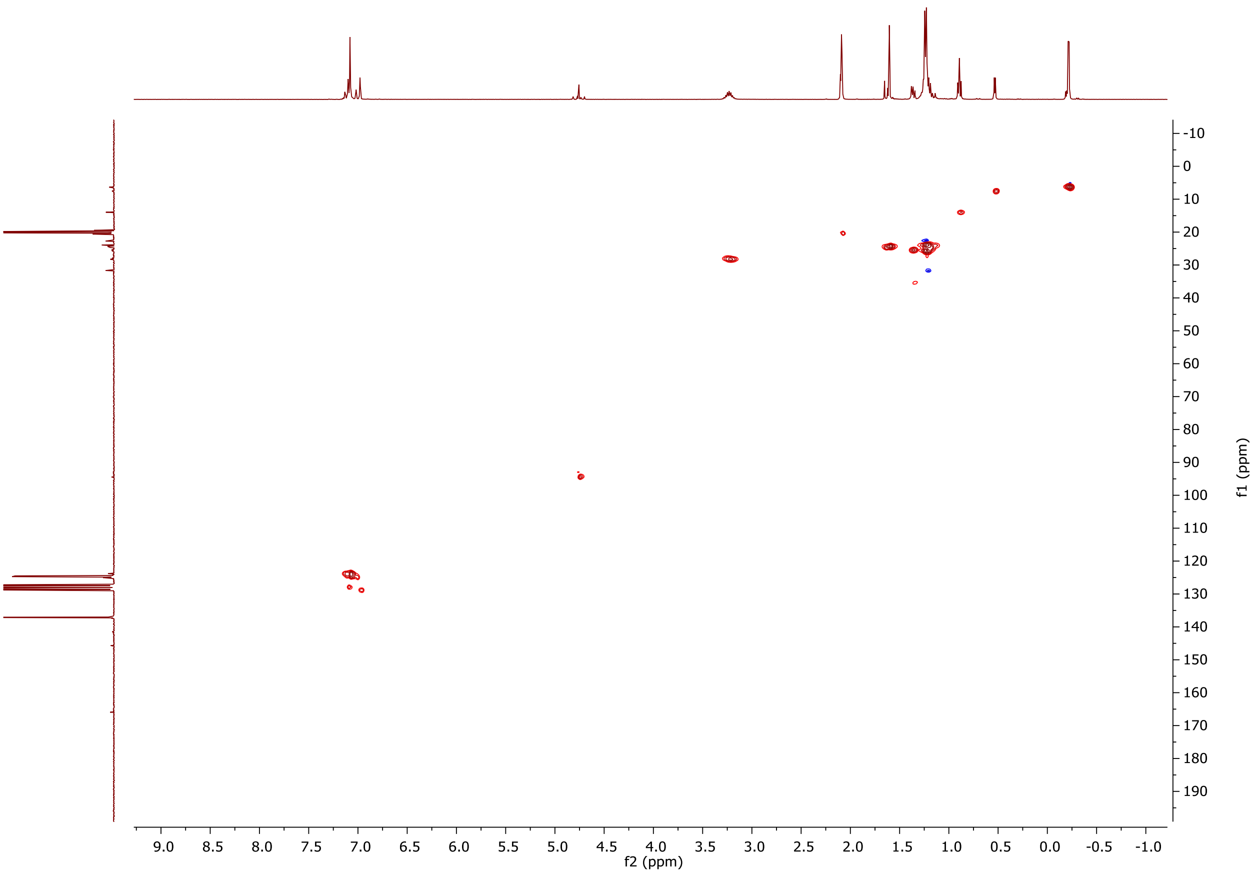

Figure S39. *^{1}H-^{13}C HSQC trace (C_7D_8, 298 K, 400.13, 100.62 MHz) for* ***[(BDI)Ca(H)P(SiMe$_3$)$_2$Ca(BDI)] (7).***

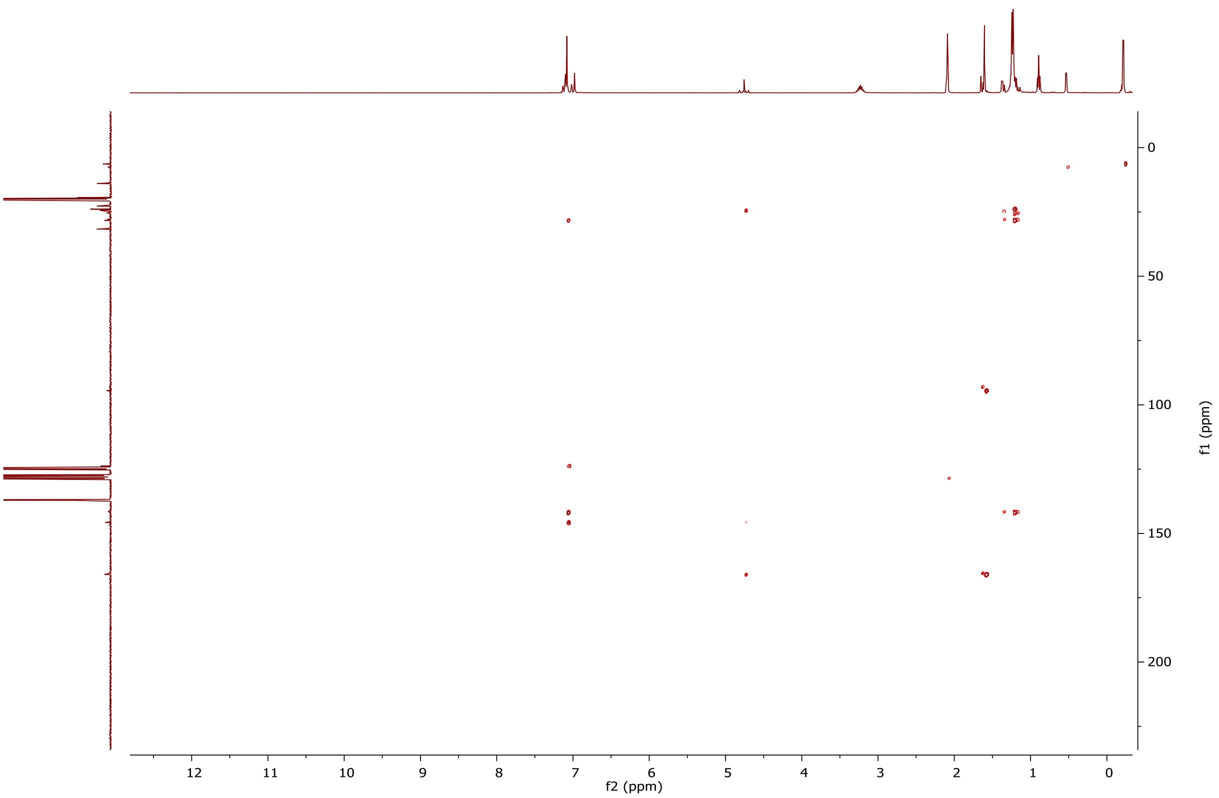

Figure S40. *^{1}H-^{13}C HMBC trace (C_7D_8, 298 K, 400.13, 100.62 MHz) for* ***[(BDI)Ca(H)P(SiMe$_3$)$_2$Ca(BDI)] (7).***

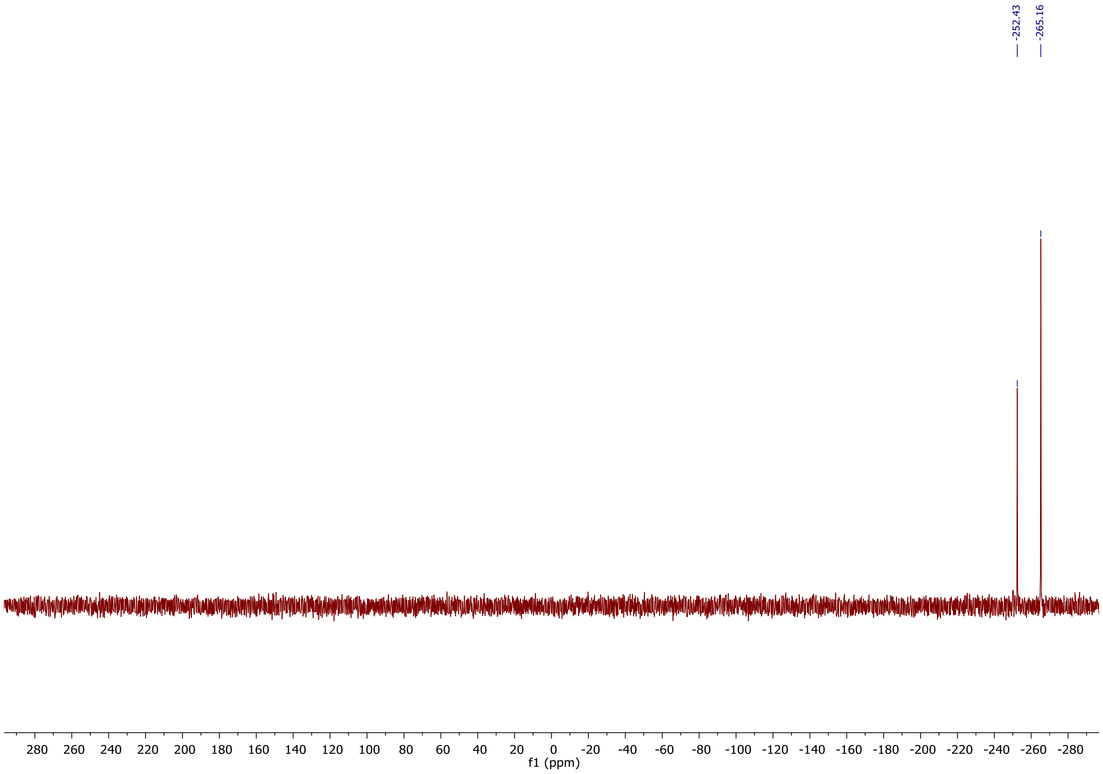

Figure S41. *$^{31}P\{^1H\}$ NMR Spectrum (C_6D_6, 298 K, 161.98 MHz) from reacting $P(SiMe_3)_3$ with $[(BDI)CaH]_2$, demonstrating residual $P(SiMe_3)_3$ and* ***9*** *formation.*

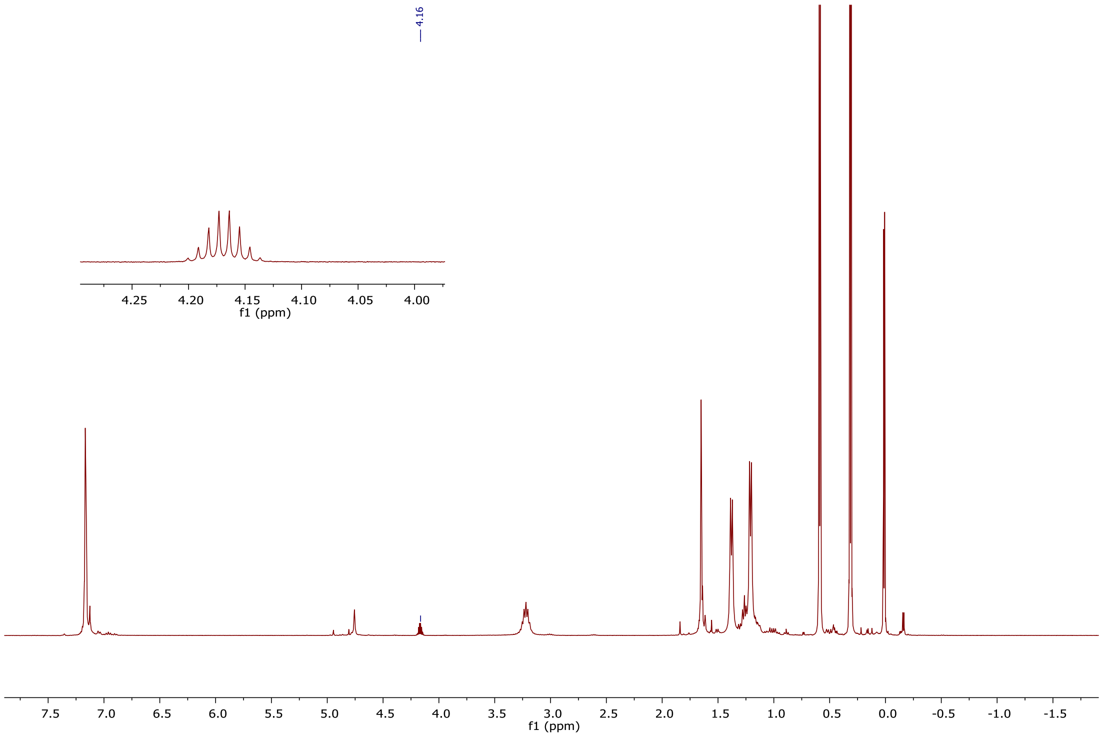

Figure S42. *^1H NMR Spectrum (C_6D_6, 298 K, 400.13 MHz) from reacting $P(SiMe_3)_3$ with $[(BDI)CaH]_2$ (**3**), demonstrating Me_3SiH formation.*

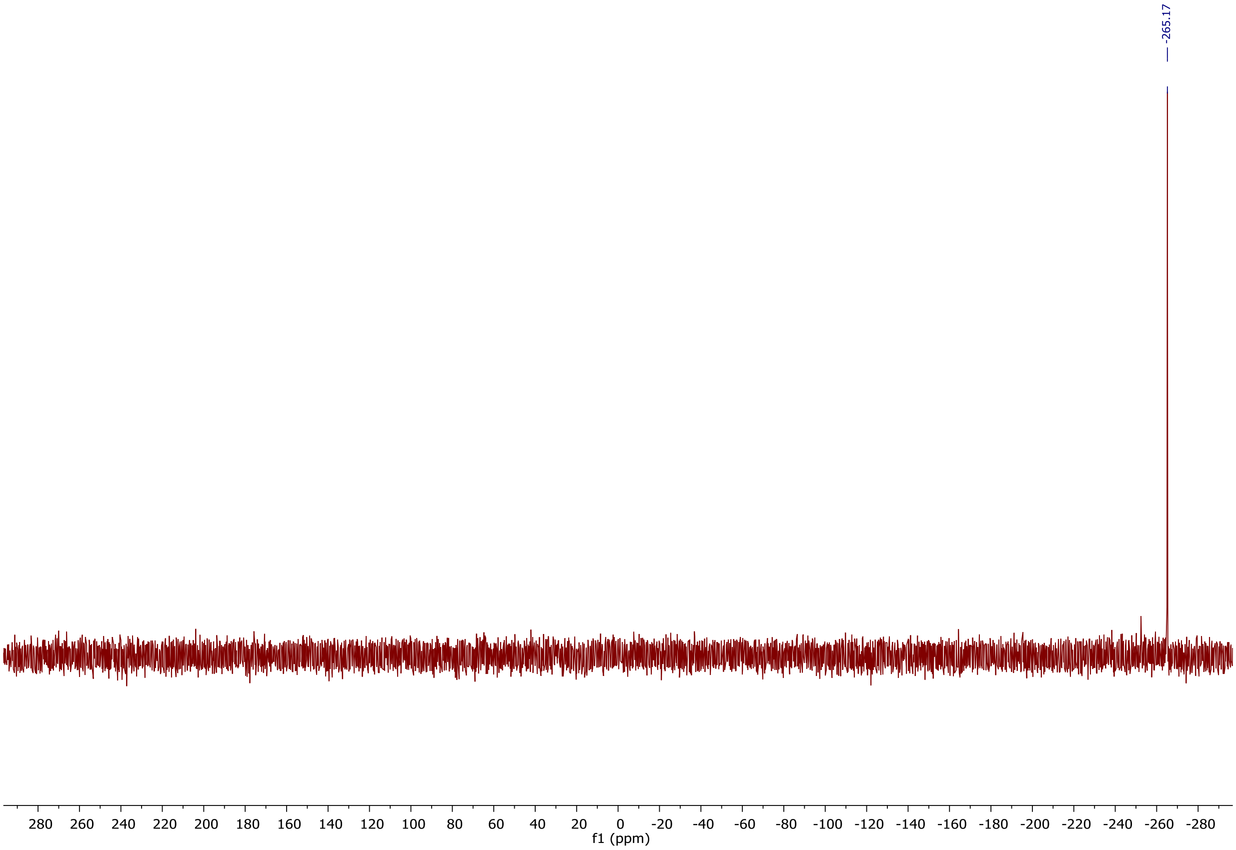

Figure S43. *^{31}P NMR Spectrum (C_6D_6, 298 K, 161.98 MHz) for* ***[(BDI)Ca(P(SiMe$_3$)$_2$]$_2$ (9)***.

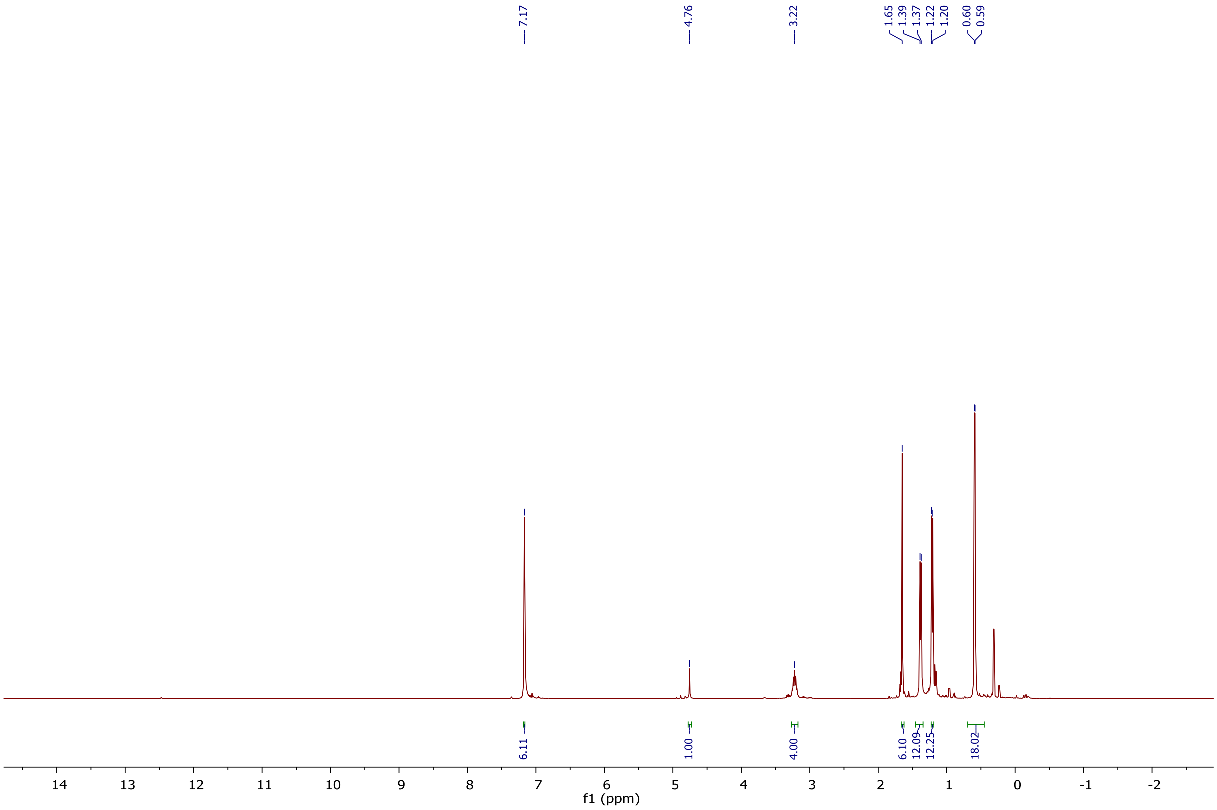

Figure S44. *^{1}H NMR Spectrum (C_6D_6, 298 K, 400.13 MHz) for* ***[(BDI)Ca(P(SiMe$_3$)$_2$]$_2$ (9)***.

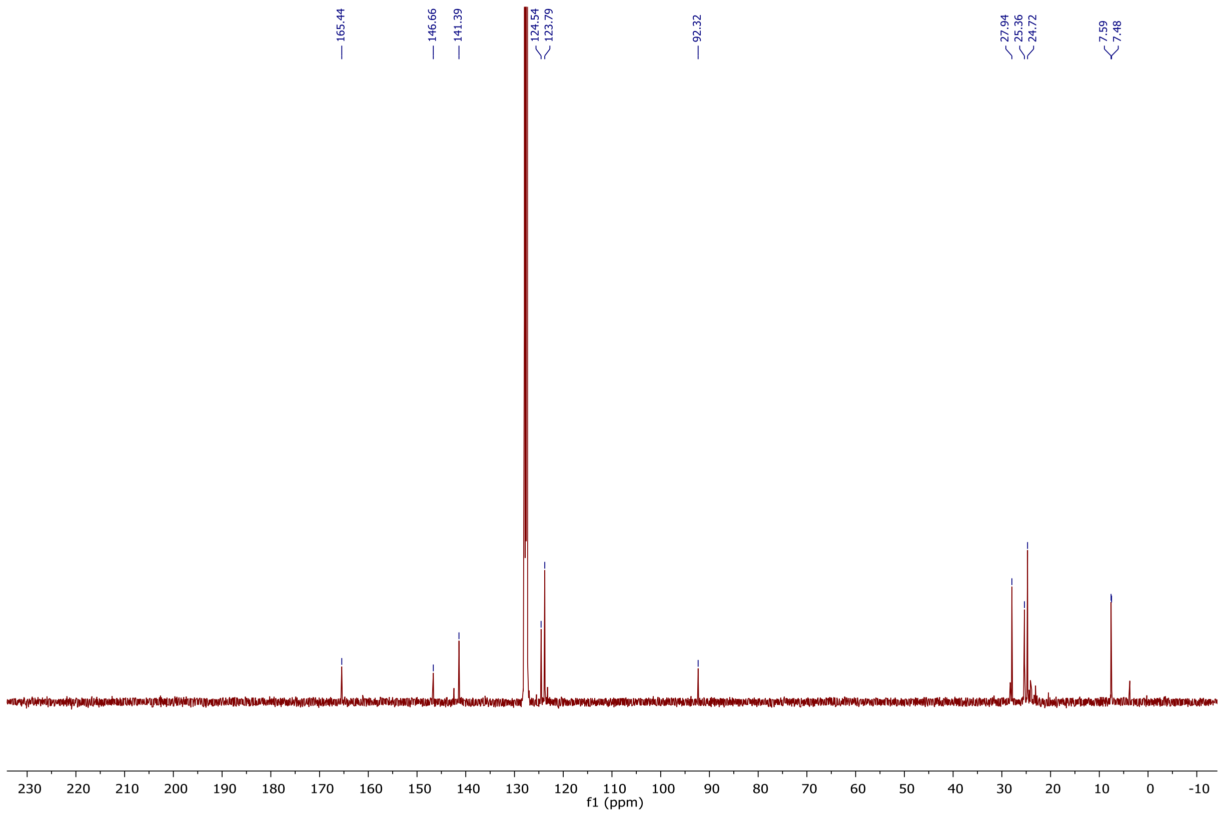

Figure S45. *$^{13}C\{^1H\}$ NMR Spectrum (C_6D_6, 298 K, 100.62 MHz) for* ***[(BDI)Ca(P(SiMe$_3$)$_2$]$_2$ (9)***.

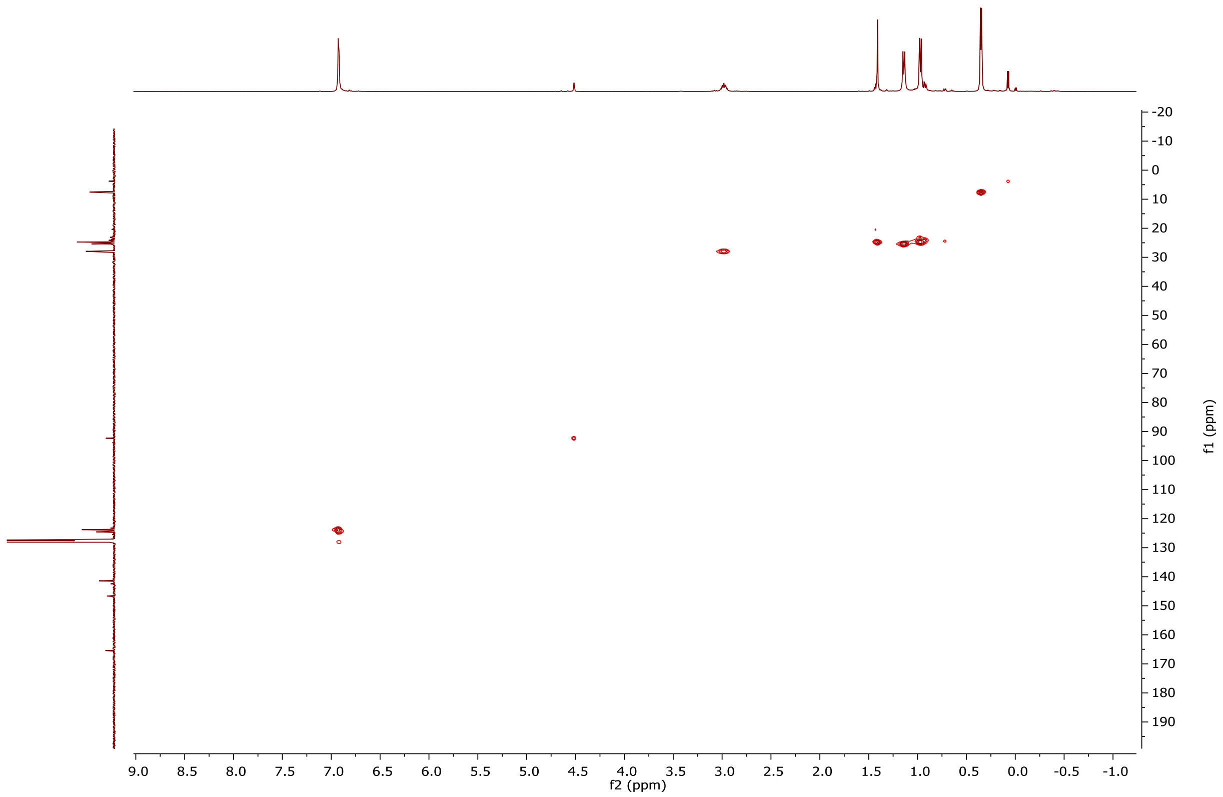

Figure S46. *^1H-^{13}C HSQC trace (C_6D_6, 298 K, 400.13, 100.62 MHz) for* ***[(BDI)Ca(P(SiMe$_3$)$_2$]$_2$ (9)***.

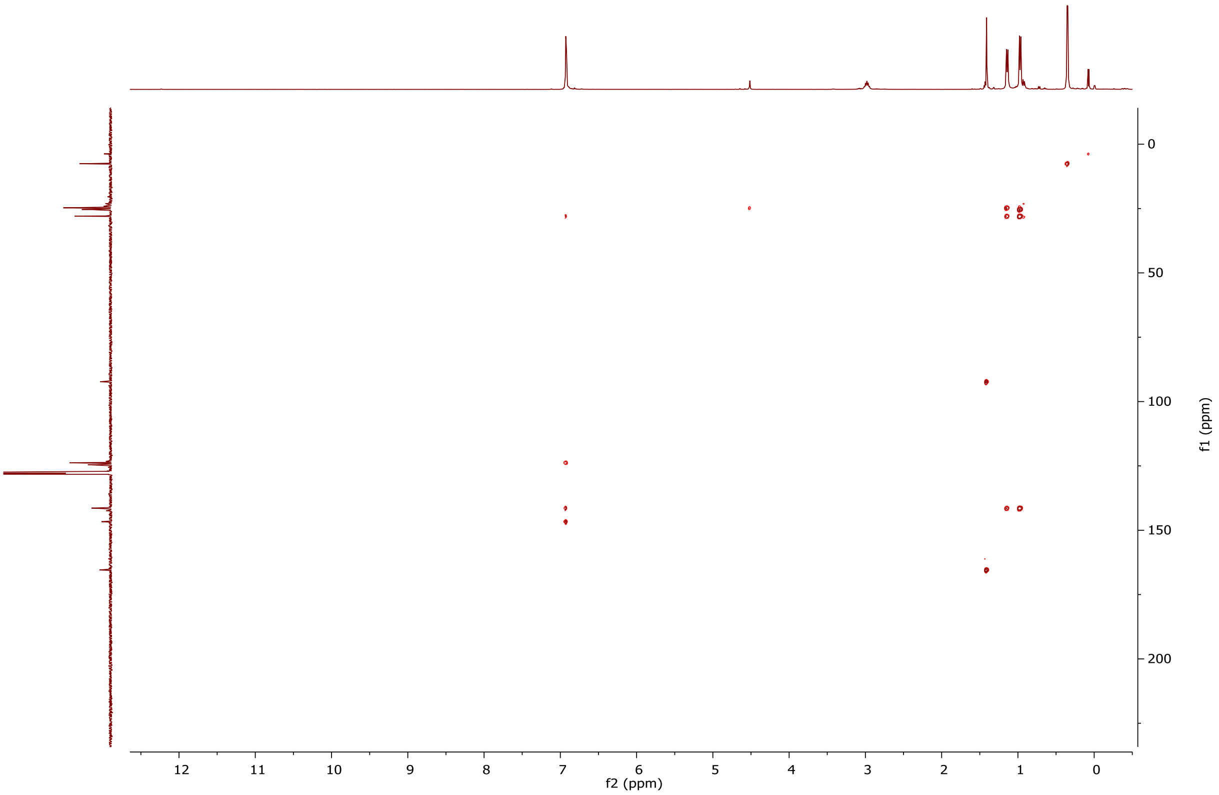

Figure S47. *^{1}H-^{13}C HMBC trace (C_6D_6, 298 K, 400.13, 100.62 MHz) for* ***[(BDI)Ca(P(SiMe$_3$)$_2$]$_2$ (9)***.

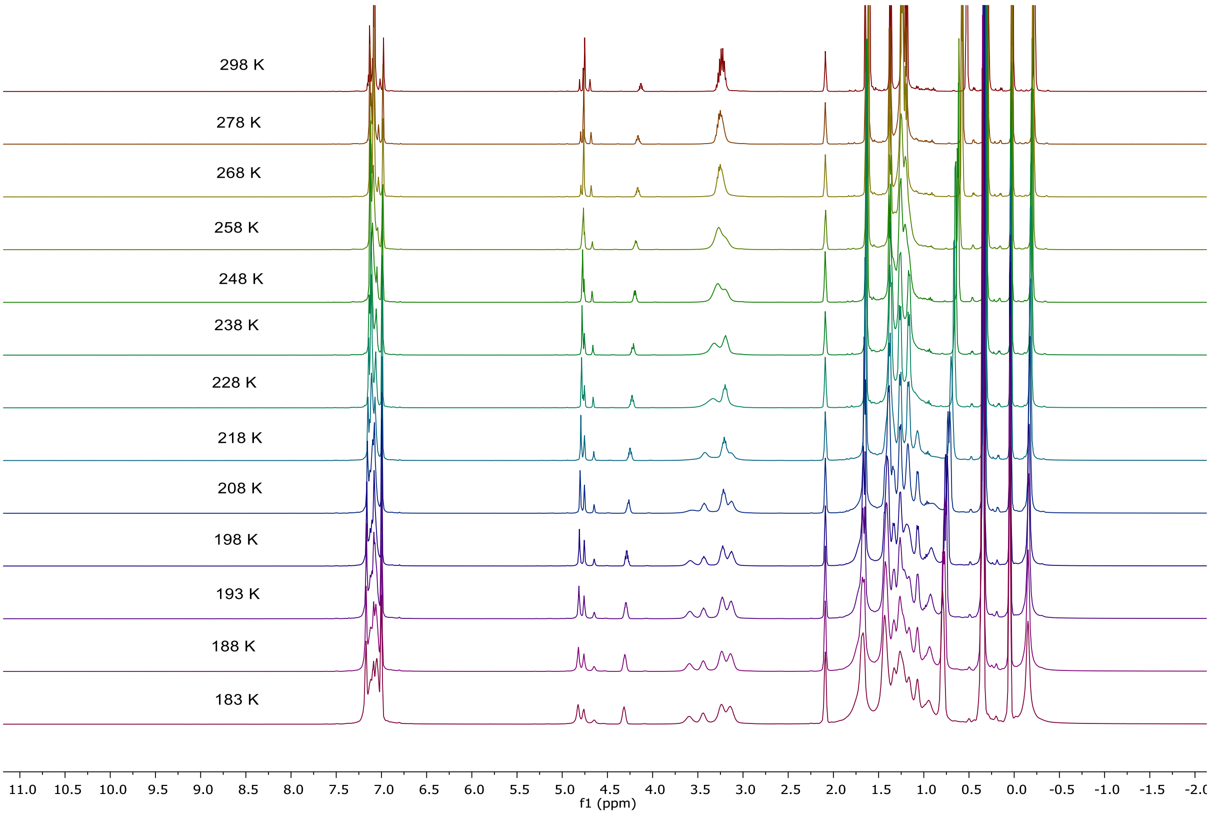

Figure S48. *Variable temperature ^{1}H NMR spectra (d_8-toluene, 400.13 MHz) of* ***[(BDI)Ca(P(SiMe$_3$)$_2$]$_2$ (9)***.

Crystallographic Data

Single crystal X-ray diffraction data for compounds **4** and **5** were collected on an Agilent SuperNova EosS2 diffractometer using Cu-Kα (1.54184 Å) radiation, while compounds **6**-**9** were collected on an XtaLAB Synergy, Dualflex, HyPix-Arc 100 diffractometer using Cu-Kα (1.54184 Å) radiation. In each case, the crystals were maintained at 150 K during data collection. Using Olex2,[1] the structures were solved with the olex2.solve[2] structure solution program or ShelXT and refined with the ShelXL[3] refinement package using Least−Squares minimisation.

The asymmetric unit in **4** comprises one molecule of the dimer and a region of solvent. The latter (due to intrinsic disorder) was treated using the solvent mask algorithm in Olex-2. An allowance of 1 molecule of hexane, per asymmetric unit, has been made in the model as presented here, in order to take account if the solvent present in the lattice.

The asymmetric unit in **5** comprises 2 crystallographically independent molecules.

The asymmetric unit in **6** comprises a calcium mixed phosphide-hydride dimer and a benzene solvent molecule. Disorder was observed on one of the Dipp isopropyl units and the benzene molecule (C67-C72), split over two disordered sites in a 40:60 and 45:55 ratio, respectively. Appropriate distance and ADP restraints were implemented to assist convergence.

The asymmetric unit for **7** is comprised of an unsymmetrical dimeric molecule, plus one molecule of hexane. Disorder was identified on one of the Dipp iso-propyl units (C50-C52; 60:40 ratio) and one of the $SiMe_3$ motifs (Si2; C33-C35; 50:50 ratio) which was readily modelled. Appropriate distance and ADP restraints were employed in these disordered regions, to assist convergence. The solvent resides within structural channels and is heavily disordered and hence was treated using a solvent mask. The latter was readily implemented, consistent with the presence of 1[C_6H_{14}] per formula unit, accounting for 200 electrons per unit cell.

The asymmetric unit in **8** is comprised of two half molecules containing calcium, zinc and phosphorus, alongside a molecule and a half of benzene. All molecules can be completed by virtue of inversion symmetry: ^{1}1-X,1-Y,1-Z; ^{2}2-X,1-Y,2-Z; ^{3}1-X,1-Y,2-Z. Disorder was identified on one of the phosphorus tert-butyl groups on each half molecule (C35-C38; C70-C73), as well as on one of the isopropyl-Dipp units on the calcium 2 containing half molecule (C62-C64), Zn2 was also disordered over two sites of occupancy, all of which were readily modelled in a 50:50 ratio. The complete molecule of benzene included within the asymmetric unit was disordered in a 60:40 ratio. Distance and ADP restraints were employed, on merit, in the disordered solvent regions to assist convergence.

The asymmetric unit for **9** is comprised of one full dimeric molecule alongside half a molecule of hexane. Disorder was identified on the Dipp-isopropyl unit (C53-C55) which was readily modelled in a ratio of 70:30. A solvent mask was implemented, consistent with the presence of half a molecule of

hexane per asymmetric unit, accounting for 100 electrons per unit cell. Distance and ADP restraints were employed on the disordered unit to assist convergence.

Crystallographic data for all compounds have been deposited with the Cambridge Crystallographic Data Centre as supplementary publications CCDC 2500991-2500996 for **4 - 9**, respectively. Copies of these data can be obtained free of charge on application to CCDC, 12 Union Road, Cambridge CB2 1EZ, UK [fax(+44) 1223 336033], e-mail: deposit@ccdc.cam.ac.uk.

Table S1. *Crystal Data and Structure Refinement for Compounds **4 - 6**.*

Identification code	s18msh25 (**4**)	s18msh28 (**5**)	y24msh11 (**6**)
Empirical formula	$C_{88}H_{116}Ca_2N_4P_2$	$C_{54}H_{73}CaN_4P$	$C_{72}H_{107}Ca_2N_4P$
Formula weight	1371.94	849.21	1139.74
Crystal system	monoclinic	triclinic	triclinic
Space group	$P2_1/c$	*P*-1	*P*-1
a/ Å	25.5636(3)	14.0584(2)	12.7963(3)
b/ Å	15.3604(2)	17.0335(2)	13.5629(3)
c/ Å	23.3496(3)	22.3711(3)	23.3060(6)
α/ °	90	95.704(1)	73.764(2)
β/ °	113.182(2)	107.107(1)	74.661(2)
γ/ °	90	101.788(1)	67.055(2)
U/ $Å^3$	8428.3(2)	4938.72(12)	3520.52(16)
Z	4	4	2
ρ_{calc}/ g cm^{-3}	1.081	1.142	1.075
μ/ mm^{-1}	1.853	1.683	1.915
F(000)	2968.0	1840.0	1244.0
Crystal size/ mm^3	0.406 × 0.316 × 0.158	0.361 × 0.255 × 0.126	0.13 × 0.1 × 0.06
2θ range for data collection/°	6.874 to 146.116	5.378 to 146.482	7.222 to 160.666
Index ranges	-31 ≤ h ≤ 30, -19 ≤ k ≤ 19, -27 ≤ l ≤ 28	-17 ≤ h ≤ 17, -19 ≤ k ≤ 21, -27 ≤ l ≤ 23	-16 ≤ h ≤ 16, -17 ≤ k ≤ 17, -29 ≤ l ≤ 26
Reflections collected	120197	70663	47933
Independent reflections, R_{int}	16804, 0.0528	19719, 0.0504	14956, 0.0509
Data/restraints/parameters	16804/0/831	19719/0/1109	14956/72/784
Goodness–of–fit on F^2	1.029	1.040	1.100
Final *R*1, *wR*2 [*I*>=2σ(*I*)]	0.0506, 0.1295	0.0410, 0.1043	0.0642, 0.1683
Final *R*1, *wR*2 [all data]	0.0569, 0.1344	0.0444, 0.1083	0.0915, 0.1948
Largest diff. peak/hole/ e $Å^{-3}$	0.65/-0.52	0.33/-0.43	0.36/-0.66

Table S2. *Crystal Data and Structure Refinement for Compounds **7 - 9**.*

Identification code	y24msh23 (**7**)	y24msh18 (**8**)	y24msh22 (**9**)
Empirical formula	$C_{70}H_{115}Ca_2N_4PSi_2$	$C_{85}H_{134}Ca_2N_4P_2Zn_2$	$C_{73}H_{125}Ca_2N_4P_2Si_4$
Formula weight	1179.96	1484.79	1313.22
Crystal system	monoclinic	monoclinic	monoclinic
Space group	$P2_1/c$	$P2_1/c$	$P2_1/c$
a/ Å	24.7464(6)	21.5633(2)	21.5180(8)
b/ Å	14.3159(3)	17.57890(10)	16.2392(4)
c/ Å	21.3007(4)	22.9668(2)	23.3637(6)
α/ °	90	90	90
β/ °	102.920(2)	103.9980(10)	100.809(3)
γ/ °	90	90	90
U/ Å^3	7355.1(3)	8447.25(12)	8019.2(4)
Z	4	4	4
ρ_{calc}/ g cm^{-3}	1.066	1.168	1.088
μ/ mm^{-1}	2.147	2.432	2.473
F(000)	2584.0	3208.0	2868.0
Crystal size/ mm^3	0.16 × 0.1 × 0.03	0.16 × 0.13 × 0.07	0.06 × 0.05 × 0.02
2θ range for data collection/°	7.18 to 140.126	6.404 to 161.272	6.668 to 133.18
Index ranges	$-30 \le h \le 30$, $-17 \le k \le 17$, $-25 \le l \le 18$	$-26 \le h \le 27$, $-22 \le k \le 20$, $-29 \le l \le 27$	$-20 \le h \le 25$, $-19 \le k \le 17$, $-27 \le l \le 21$
Reflections collected	85566	112672	69514
Independent reflections, R_{int}	13707, 0.0954	18394, 0.0399	13569, 0.1297
Data/restraints/parameters	13707/65/757	18394/284/1065	13569/27/800
Goodness–of–fit on F^2	1.062	1.031	1.058
Final R1, wR2 [I>=2σ(I)]	0.0566, 0.1458	0.0506, 0.1397	0.0696, 0.1686
Final R1, wR2 [all data]	0.0854, 0.1597	0.0550, 0.1433	0.1190, 0.1886
Largest diff. peak/hole/ e Å^{-3}	0.47/-0.72	0.71/-1.00	0.43/-0.30

References

1. O. V. Dolomanov, L. J. Bourhis, R. J. Gildea, J. A. K. Howard and H. Puschmann, *J. Appl. Cryst.* 2009, **42**, 339-341.
2. G. M. Sheldrick, *Acta Cryst*. 2015, **A71**, 3-8.
3. G. M. Sheldrick, *Acta Cryst*. 2015, **C71**, 3-8.
